# Fibroblast activation protein identifies Consensus Molecular Subtype 4 in colorectal cancer and allows its detection by ^68^Ga-FAPI-PET imaging

**DOI:** 10.1038/s41416-022-01748-z

**Published:** 2022-03-16

**Authors:** Esther Strating, Emma Wassenaar, Mathijs Verhagen, Paulien Rauwerdink, Susanne van Schelven, Ignace de Hingh, Inne Borel Rinkes, Djamila Boerma, Arjen Witkamp, Miangela Lacle, Riccardo Fodde, Richard Volckmann, Jan Koster, Kris Stedingk, Frederik Giesel, Remmert de Roos, Alex Poot, Guus Bol, Marnix Lam, Sjoerd Elias, Onno Kranenburg

**Affiliations:** 1 Department of Surgical Oncology, Lab Translational Oncology, University Medical Center Utrecht, Utrecht University, Utrecht, The Netherlands; 2 Department of Surgery, St. Antonius Hospital, Nieuwegein, The Netherlands; 3 Department of Pathology, Erasmus MC, Rotterdam, Netherlands; 4 Department of Surgery, Catharina Hospital, Eindhoven, The Netherlands; 5 Department of Pathology, University Medical Center Utrecht, Utrecht University, Utrecht, The Netherlands; 6 Department of Oncogenomics, Amsterdam University Medical Center, Amsterdam, The Netherlands; 7 Department of Nuclear Medicine, University Hospital Heidelberg, Heidelberg, Germany; 8 Department of Nuclear Medicine, Medical Faculty, Heinrich-Heine-University, University Hospital Dusseldorf, Dusseldorf, Germany; 9 Department of Radiology, University Medical Center Utrecht, Utrecht University, Utrecht, The Netherlands; 10 Department of Medical Oncology, University Medical Center Utrecht, Utrecht University, Utrecht, The Netherlands; 11 Department of Epidemiology, Julius Center for Health Sciences and Primary Care, University Medical Center Utrecht, Utrecht University, Utrecht, The Netherlands; 12 Utrecht Platform for Organoid Technology, Utrecht University, Utrecht, The Netherlands

**Keywords:** Colorectal cancer, Cancer imaging

## Abstract

**Background:**

In colorectal cancer (CRC), the consensus molecular subtype 4 (CMS4) is associated with therapy resistance and poor prognosis. Clinical diagnosis of CMS4 is hampered by locoregional and temporal variables influencing CMS classification. Diagnostic tools that comprehensively detect CMS4 are therefore urgently needed.

**Methods:**

To identify targets for molecular CMS4 imaging, RNA sequencing data of 3232 primary CRC patients were explored. Heterogeneity of marker expression in relation to CMS4 status was assessed by analysing 3–5 tumour regions and 91.103 single-tumour cells (7 and 29 tumours, respectively). Candidate marker expression was validated in CMS4 peritoneal metastases (PM; *n* = 59). Molecular imaging was performed using the ^68^Ga-DOTA-FAPI-46 PET tracer.

**Results:**

Fibroblast activation protein (FAP) mRNA identified CMS4 with very high sensitivity and specificity (AUROC > 0.91), and was associated with significantly shorter relapse-free survival (*P* = 0.0038). Heterogeneous expression of FAP among and within tumour lesions correlated with CMS4 heterogeneity (AUROC = 1.00). FAP expression was homogeneously high in PM, a near-homogeneous CMS4 entity. FAPI-PET identified focal and diffuse PM that were missed using conventional imaging. Extra-peritoneal metastases displayed extensive heterogeneity of tracer uptake.

**Conclusion:**

FAP expression identifies CMS4 CRC. FAPI-PET may have value in the comprehensive detection of CMS4 tumours in CRC. This is especially relevant in patients with PM, for whom effective imaging tools are currently lacking.

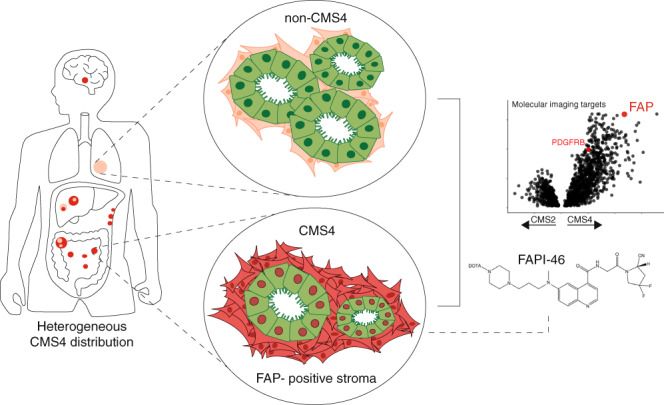

## Introduction

Transcriptional profiling of large cohorts of colorectal tumours has resulted in the generation of a consensus molecular classification (CMS) system for CRC, consisting of four distinct subtypes (CMS1-4) [1]. Patients with CMS4 tumours have the worst prognosis and benefit least from systemic therapy [1–5]. The CMS system, and the insight into the biological characteristics of the distinct subtypes that it offers, has created a novel framework for the design of subtype-targeted therapeutic strategies. For instance, the treatment benefit offered by immune checkpoint inhibitors is restricted to the subtype of metastatic CRC with a defective DNA mismatch repair system (dMMR), which is largely contained within CMS1 [6]. Key to the success of subtype-targeted therapy is the availability of robust diagnostic tools for upfront patient selection. In the case of patients with dMMR CRC, multiple diagnostic tools measuring microsatellite instability (MSI)—a direct consequence of dMMR—are available for patient selection, and these are routinely used in clinical practice worldwide. Importantly, a single biopsy from a primary tumour or a metastasis is sufficient to assess tumour MSI status of all tumour lesions in an individual patient [7]. This is presumably because dMMR is hard-wired into the DNA of all tumour lesions and not dependent on contextual or temporal variables. By contrast, CMS4 status can vary between primary and metastatic lesions in individual patients [5, 8, 9], and between different regions in a single lesion [10–12]. Moreover, CMS status may change over time as a result of chemotherapy [5, 13]. The currently available diagnostic tools for CMS4 identification involve the molecular, immunohistochemical or imaging-based analysis of one or more tumour biopsies and/or sections thereof [4, 11, 14, 15]. However, interpretation of the results generated by all these methods is complicated by the regional and temporal variability of tumour CMS4 status in individual patients. None of these methods is currently being used in a routine clinical setting.

We hypothesised that the problem of regional CMS4 variability could be resolved by developing a molecular imaging strategy that would allow the determination of CMS4 status of all possible tumour lesions in individual patients with a single scan. Repeat scanning would furthermore allow assessment of temporal variation induced by (for instance) chemotherapy. We therefore set out to identify cell surface markers on CMS4 CRC that could function as targets for molecular imaging. We identified fibroblast activation protein (FAP) as one such marker and provide proof-of-concept in cancer patients that molecular imaging of FAP with the ^68^Ga-DOTA-FAPI-46 PET tracer [16] is a promising strategy to quantify ‘CMS4 tumour load’ in CRC patients, and—possibly—to evaluate the response of these lesions to systemic therapy.

## Materials and methods

### Patient series

We made use of the following CRC cohorts with RNA sequencing data: First, a composite cohort of 3232 primary CRC tumours, which was used to generate the CMS classification system [1]. Second, a cohort of multiple biopsies taken from randomly selected non-adjacent tumour areas of primary CRC tumours [11]. Third, a composite cohort of more than 90 thousand single cells derived from 29 colorectal tumours and adjacent normal tissue with annotated cellular identities [17]. Fourth, a cohort of CRC primary tumour regions (*n* = 35) and paired peritoneal metastases (*n* = 59) [18]. Fifth, FFPE tissue from a cohort of colorectal liver metastasis patients (CRLM, *n* = 24) [5]. And lastly, a cohort of normal liver tissue derived single cells [19]. An overview of all patient cohorts and their application in this study can be found in Supplementary Fig. 1.

### CMS classification

Molecular classification of tumour samples was performed by using the random forest CMS classifier described in Guinney et al. [1]. The CMSclassifier R package v.1.0.0 was downloaded from Github (https://github.com/Sage-Bionetworks/CMSclassifier).

### Immunohistochemistry

Formalin-fixed paraffin-embedded sections were deparaffinized, rehydrated and incubated with 1.5% hydrogen peroxide solution for 20 min to block endogenous peroxidase activity. Antigen retrieval was obtained by boiling the sections in a citrate buffer (pH 6). Sections were incubated overnight at 4 C with one of the following primary antibodies: anti-FAP (ab207178 Abcam, dilution 1:400), anti-EpCAM (CS 14452 Cell Signaling, dilution 1:200), anti-ZEB1 (HPA027524 Sigma, 1:500) or anti-HTR2B (HPA012867 Sigma, 1:75). Bound antibodies were detected by a poly-HRP-labelled goat anti-rabbit secondary antibody (Immunologic, VWRKDPVR110HRP). The sections were developed with DAB chromogen and counterstained with hematoxylin.

FAP protein expression was quantified on whole IHC slides using QuPath v.0.3.0 [20]. Scanned sections were analysed using QuPath’s positive cell detection command. DAB staining intensity thresholds were selected manually and applied to all slides. Next, a classifier was trained to distinguish between tumour cells, stromal cells and all other cells. For all slides, the total number of FAP-positive cells, the percentage of FAP-positive stromal cells and the percentage of FAP-positive tumour cells were assessed. To be able to correct tumour size, sequential slides were stained for EpCAM and quantified using positive cell detection.

### ^68^Ga-FAPI-PET imaging

FAPI-46 precursor for labelling was supplied by SOFIE Biosciences (Totowa, NJ, USA), ascorbic acid was obtained from Polatom (Otwoch, Poland). Gallium-68 radiolabeling was performed using a clinically approved ^68^Ge/^68^Ga-generator (Galliapharm^®^) and a reagent kit on a Modular Eazy cassette system (Eckert & Ziegler, Berlin, Germany).

For quality control of the product, radiochemical purity was analysed by iTLC (MiniGita, Elysia-Raytest, Liege, Belgium) and a HPLC system of Thermo Scientific (Agilent, Santa Clara, CA, USA). pH was measured by a Quantofix Relax pH stripreader (Machery-Nagel, Düren, Germany), endotoxin content was determined on a Endosafe nexgen-PTS (Charles River, Den Bosch, The Netherlands) followed by sterility testing.

For clinical production of ^68^Ga-DOTA-FAPI-46, the procedure as described by Spreckelmeyer et al. was followed [21]. Radiochemical purity of ^68^Ga-DOTA-FAPI-46 was >99% both on iTLC and radioHPLC. The product was sterile and passed endotoxin testing. In the ammonium acetate buffer, ^68^Ga-DOTA-FAPI-46 was stable for at least 3 h after production based on radioHPLC analysis.

The patient was referred for a ^68^Ga-DOTA-FAPI-46 PET/CT by the oncologist who at that time faced a diagnostic challenge that could not be solved with standard diagnostic imaging. After obtaining the patient’s informed consent PET images were made 1 h after intra-venous injection of [^68^Ga]-DOTA-FAPI-46 at 1.5 MBq/kg injected dose. The FAPI-PET signal was quantified according to the EARL reconstruction standards.

### Intra-patient inter-lesion heterogeneity of FAPI uptake

FAPI-PET signals of 15 colorectal cancer patients with multiple extra-peritoneal metastases [22] were re-analysed to assess SUVmax values on a per-lesion basis to assess intra-patient inter-lesion heterogeneity of tracer uptake.

### FAP expression in single-cell RNAseq data

Count matrices from Lee et al. [17] were retrieved from the gene expression omnibus under accession numbers GSE144735 and GSE132465. The data was processed using sctransform as implemented in Seurat v4 [23]. The cells were embedded with umap dimension reduction using the first 30 components. For differential expression analysis, cells with a non-zero value of FAP counts were labelled as FAP positive. Subsets were made separating the epithelial and stromal compartments based on the authors annotations, before differential expression analysis was performed with the FindMarkers function using default parameters (wilcox test, logfc.threshold = 0.25). To correlate FAP expression with other stromal signatures, the indicated gene signatures from references [24, 25] were converted into scores using the AddModuleScore function. Next, Pearson correlations were computed between the stromal signatures and scaled FAP expression. To visualise the FAP expression patterns across CMS types, we labelled the patients according to their predicted CMS based on bulk RNAseq predictions as described in the paper. Then, we grouped the cells according to the CMS and annotated cell subtypes, and visualised the fraction of the cell subtypes across the CMS groups with the matching average scaled expression of FAP. To gain insight in the epithelial to the mesenchymal state of the sequenced epithelial cells we assigned epithelial scores to the cells based on the expression of a signature reflecting epithelial differentiation [26]. After imputation of the count matrix with MAGIC (knn = 5, *t* = 3), an epithelial score was computed by taking the average of the z-score expression values from the Epithelial signature. The imputed FAP expression level was compared to the Epithelial score using Pearson Correlation.

### Statistical analysis

All statistical analyses were conducted in R version 4.0.5. Pearsons Correlation was used to assess the correlation between FAP expression and the CMS4 identifying geneset. Survival curves were calculated using the Kaplan–Meier method and tested for significance with the log-rank test. FAP protein quantification values were compared using the Mann–Whitney *U* test.

## Results

### FAP mRNA expression identifies CMS4 CRC

**Fig. 1:**
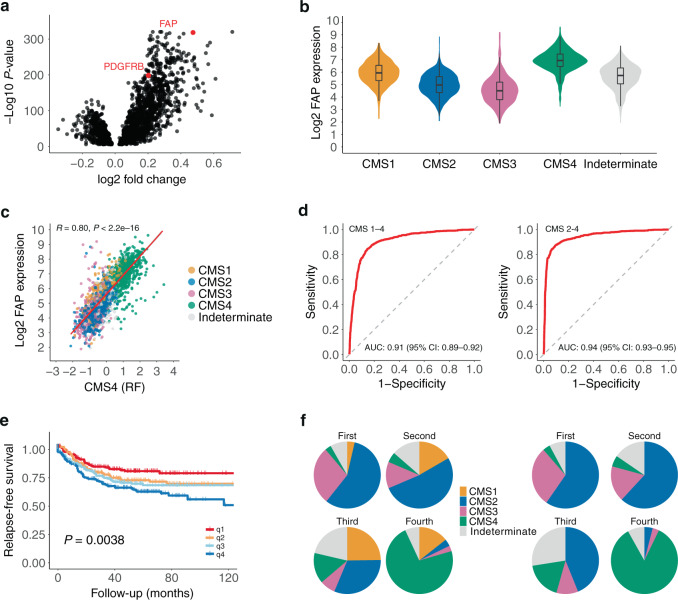
FAP mRNA expression identifies CMS4 CRC. **a** Volcano plot showing differentially expressed genes between CMS2 and CMS4 subgroups that encode plasma membrane proteins. All individual genes and accompanying *P* and fold-change values are presented in Supplementary Table S1. **b** Violin plot showing FAP mRNA expression in the CMS subgroups in a large composite cohort consisting of 3232 primary tumours [1]. **c** Scatter plot showing the correlation between FAP mRNA expression and the CMS4-identifying geneset in the random forest CMS classifier (*n* = 143 genes; CMS4(RF)) in the CMS-3232 cohort. **d** Receiver-operating characteristic (ROC) curves showing the sensitivity and specificity of using FAP mRNA levels to distinguish between CMS4 and either all other tumours in the cohort (left curve) or all tumours except CMS1 (right curve). In clinical practice, the vast majority of CMS1 tumours are routinely identified with MSI tests. The area under the curve (AUC) values are shown, as a measure of diagnostic accuracy of the FAP test. **e** Kaplan–Meier curves showing relapse-free survival probabilities in tumour subgroups defined by FAP expression quartiles, *n* = 805 patients. **f** Pie charts showing the CMS distribution of tumour subgroups defined by FAP expression quartiles in the same tumour groups as in (**e**) with (left panel) and without (right panel) patients with CMS1 tumours. The vast majority of patients with CMS1 tumours will be identified by MSI testing as part of routine diagnostic procedures.

To identify candidate biomarkers for imaging-based comprehensive CMS4 detection, we performed differential gene expression analysis, focusing only on genes encoding plasma membrane-localised proteins (gene ontology ID: GO:0005886). We identified 1009 genes that were expressed at significantly higher levels in ‘mesenchymal’ CMS4 tumours (*n* = 770) when compared to traditional ‘epithelial’ CMS2 tumours (*n* = 1110) (Supplementary Table S1). We found that fibroblast activation protein (FAP), a relatively new molecular imaging target [16], ranked fifth in this list (*P* = 5.9E–319) (Fig. 1a and Supplementary Table S1). PDGFRB was the only other candidate target for which molecular imaging tracers are currently available [27, 28] in the top 125 of this list (*P* = 1.10E-199; Fig. 1a and Supplementary Table S1).

FAP mRNA expression was significantly higher in CMS4 than in any of the other subtypes (CMS1-3) (Fig. 1b) and correlated extremely well with the expression of the CMS4-identifying genes within the original random forest classifier (Fig. 1c). FAP expression was an excellent predictor of CMS4 in a composite dataset of 3232 primary tumours [1], with an area under the receiver-operating characteristic curve (AUROC) of 0.91 (95% confidence interval [CI] = 0.89–0.92) (Fig. 1d). We noted that FAP expression was also high in a subset of CMS1 tumours, a subtype largely consisting of tumours with high levels of microsatellite instability (MSI-H). Molecular and immunohistochemistry tests for the identification of MSI-H tumours are commonly applied in most molecular pathology labs. Without CMS1 tumours, the discriminative power of FAP expression further increased to 0.94 (Fig. 1d).

FAP expression was associated with a significantly shorter relapse-free survival (Fig. 1e). Tumours expressing high FAP levels (top 25%, q1) consisted mostly of CMS4 tumours (*n* = 144; 72%) (Fig. 1f).

We conclude that FAP is an excellent single-gene identifier of CMS4 CRC.

### FAP mRNA identifies CMS4 regions in heterogeneous tumours

**Fig. 2:**
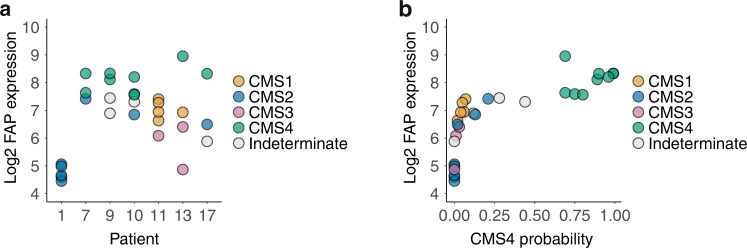
FAP identifies CMS4 regions in heterogeneous tumours. **a** Dot plot showing FAP expression levels in multiple [3–5] distinct regions of the primary tumours of seven individual CRC patients (BOSS1 cohort) analysed by RNA sequencing. CMS status, identified by applying the random forest classifier, is colour-coded. **b** Scatter plot showing the correlation of FAP mRNA levels with CMS4 probabilities of each of the tumour regions across the entire dataset. The ROC curve is not shown because the discriminative ability of FAP mRNA levels (CMS4 versus all other regions) is perfect in this dataset (AUC = 1).

The CMS classification system provides a novel paradigm for the development of personalised treatment. However, CMS status can vary between multiple regions within a single tumour [10, 11]. If FAP is to be used as a target for comprehensive CMS4 detection, its expression should follow such heterogeneity. To test this, we made use of the CRC ‘BOSS1 cohort’, in which 3–5 distinct regions of seven primary colorectal tumours were analysed by RNA sequencing. FAP expression was highly heterogeneous among and within individual tumours (Fig. 2a). CMS4 probabilities, calculated for each individual tumour region by applying the random forest CMS classifier, show a strong correlation with FAP expression in those same regions (Fig. 2b). FAP expression identified CMS4 regions in the BOSS cohort with an AUROC of 1.0 (perfect).

### Tumour-selective expression of FAP in myofibroblasts, endothelial cells and a subset of cancer cells

**Fig. 3:**
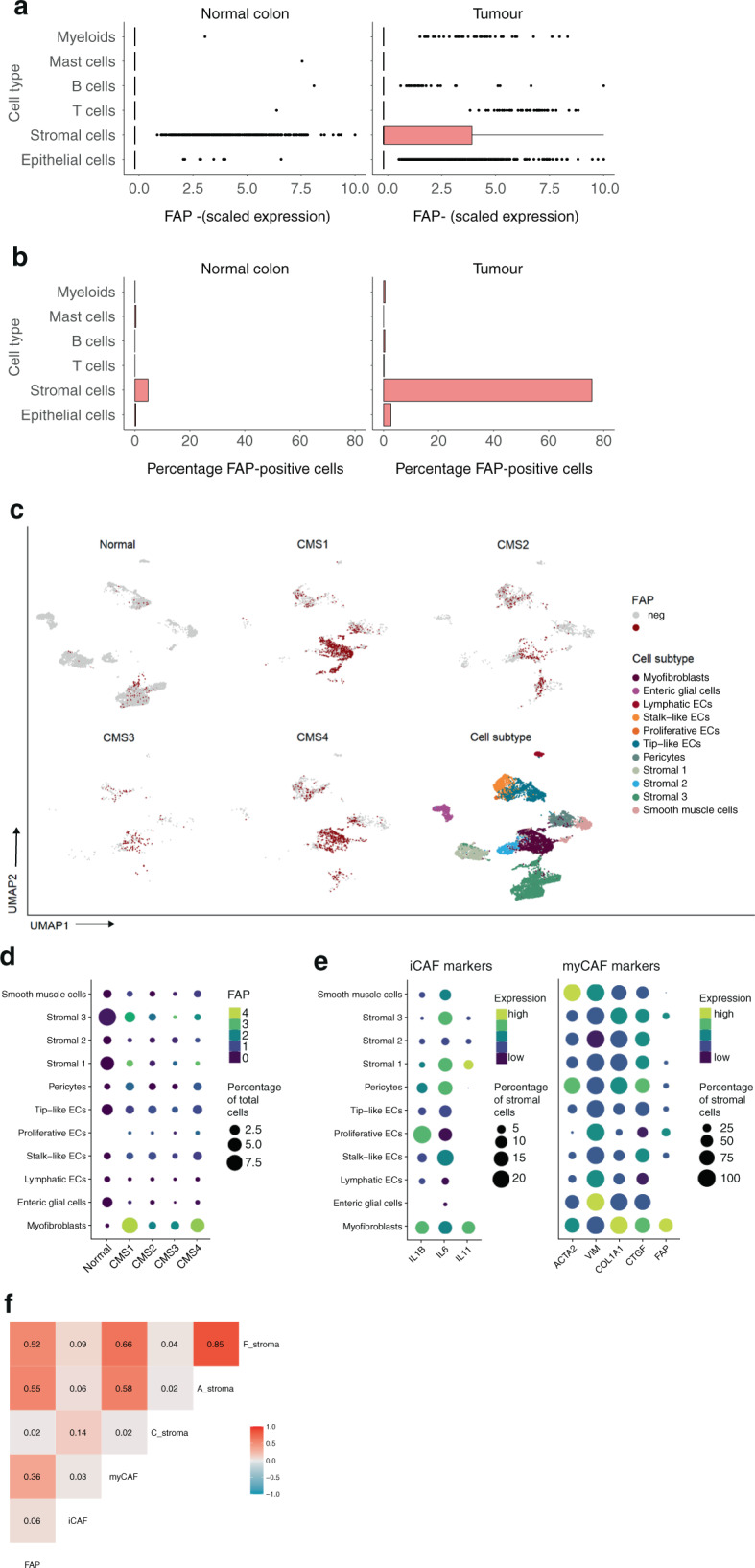
FAP is expressed in myofibroblasts, proliferating endothelial cells, and a subset of tumour cells. **a** Box plots showing FAP expression in single cells from colorectal tumours and adjacent normal colon tissue with annotated cellular identities [17]. **b** Distribution of FAP-expressing cells per cell type in normal and tumour tissue. **c** UMAP plot of all stromal cell types showing the distribution of FAP-positive cells for normal colon cells and each individual CMS. **d** Gene expression dot plot showing FAP expression distribution within the stromal subtypes for each CMS. Dot size indicates the number of stromal cells as a percentage of the total cells sequenced. Dot colour indicates the mean scaled FAP expression within the cell type. **e** Gene expression dot plot showing the gene expression of inflammatory CAF (iCAF) and myofibroblast CAF (myCAF) markers across different stromal cell types showing that FAP expression is highly selective for the myofibroblast cell type. Dot size indicates the number of stromal subtypes as a percentage of the total stromal cell fraction. Dot colour indicates the mean scaled expression within the cell type. **f** Correlation matrix showing the correlation between FAP expression, iCAF markers, myCAF markers and three distinct stromal subtypes in pancreatic cancer (C stroma, A stroma, F stroma) [24].

The high concordance of FAP expression with CMS4 status urged us to identify the cell types that contribute to FAP expression. In addition, if FAP is to be used as a tumour-specific target for molecular imaging, its expression in healthy normal tissue should be very low. Therefore, we analysed FAP expression in a composite cohort of more than 90 thousand single cells derived from 29 colorectal tumours and adjacent normal tissue with annotated cellular identities [17]. FAP expression in normal colon tissue was limited to a small subpopulation (5%) of stromal cells (Fig. 3a).

In tumour tissue, however, approximately three-quarters of all stromal cells (76%) expressed detectable levels of FAP mRNA (Fig. 3a, b). The vast majority of these cells were myofibroblasts (Fig. 3c). FAP-expressing stromal cells were detected in all CMS4 and all CMS1 tumours (Supplementary Fig. S3A). This is in line with the data presented in Fig. 1d showing that the group of CRC tumours expressing the highest levels of FAP mRNA (top 25%) largely consists of CMS4 and CMS1 tumours. However, bulk expression data show that FAP expression in CMS4 tumours is significantly higher than that in CMS1 tumours (Fig. 1a). This suggests that the average FAP expression per individual stromal cell may be similar in CMS1 and CMS4 tumours but that the number of FAP-expressing cells is significantly higher in CMS4 CRC.

We next analysed the distribution of FAP expression in stromal cell types for each individual CMS by generating UMAP plots (Fig. 3c) and dot plots (Fig. 3d) per CMS group with the colour-coded expression of FAP. These analyses show that FAP-expressing myofibroblasts are predominantly found in CMS1 and CMS4, while FAP-expressing endothelial cells, pericytes and smooth muscle cells were more evenly distributed across the CMS groups (Fig. 3c, d). Although the myofibroblasts in CMS1 and CMS4 expressed comparable levels of FAP, single-cell data cannot be used to infer total tumour levels of FAP expression, and hence the ability of FAP expression to discriminate between CMS1 and CMS4. This is because the ratios between cell types may change during tissue processing and because the number of tumours in each group is small. The bulk RNAseq data of thousands of tumours presented in Fig. 1 (and the ROC curves derived from this data) are more suitable for this. However, it is clear that some CMS1 tumours, like CMS4 tumours, contain a considerable proportion of FAP + myofibroblasts. Of note, in the entire cohort (CMS1-4), FAP expression was far more selective for myofibroblasts than commonly used markers such as ACTA2, VIM or COL1A1 (Fig. 3e).

Next, we analysed how FAP-expressing cells were related to previously identified CAF subtypes, including inflammatory and myofibroblast-like CAFs [25] and three distinct stroma subtypes in pancreatic cancer [24]. Signature scores for these distinct stromal cell subtypes were generated and correlated with FAP expression in the single-cell CRC cohort. These analyses revealed that FAP expression in CRC is most closely related to FAP- and ACTA2-expressing stromal cells in pancreatic cancer (*R*^2^ = 0.53 and 0.55, respectively) and least to inflammatory CAFs (*R*^2^ = 0.06) (Fig. 3f). To gain insight into a possible function of FAP-positive versus FAP-negative stromal cells in CRC we performed an unbiased analysis of differential gene expression. We identified 395 genes that were significantly higher expressed in FAP-expressing stromal cells (Supplementary Table S2). Functional analysis revealed high expression of genes encoding extracellular matrix (ECM) components, including 15 collagen chains (e.g. COL1A1; COL1A2; COL5A2), fibronectin (FN1), thrombospondin-2 (THBS2), 7 ECM-degrading metalloproteases (e.g. MMP1, MMP3) and TGFβ ligands and TGFβ-target genes (e.g. collagens and CTGF) (Supplementary Table S2 and Supplementary Fig. S2A). Conversely, genesets related to antigen presentation, oxidative phosphorylation, and transcription factors stimulating cell proliferation (e.g. Myc, Jun, Fos) were all downregulated in FAP-expressing stromal cells (Supplementary Table S2 and Supplementary Fig. S2B). Together, these analyses point to a classical fibrotic function for the majority of FAP-expressing stromal cells in CRC.

Of note, ~3% of (epithelial) tumour cells showed atypical expression of FAP (Fig. 3a, b and Supplementary Fig. S3B). This could reflect the presence of mesenchymal-like tumour cells, resulting from a (partial) EMT. Indeed, we found that an epithelial differentiation signature [26] was inversely correlated with FAP expression in individual tumour cells (Supplementary Fig. S3C). Interestingly, the vast majority of FAP-expressing tumour cells were derived from a tumour (SMC20) that was classified as CMS4 (Supplementary Fig. S3A, B). Differential gene expression analysis of FAP-positive and FAP-negative tumour cells in patient SMC20 identified 388 genes that were significantly upregulated in FAP-expressing tumour cells. Functional analysis of this geneset revealed an enrichment of genes involved in RNA splicing and protein translation, oxidative phosphorylation, and the cellular response to stress (Supplementary Fig. S4A and Supplementary Table S3). The latter group includes key genes involved in the protection of tumour cells against reactive oxygen species (GPX2, SOD1, PRDX1), a phenotype that we have previously linked to CMS4 CRC [29, 30]. Conversely, FAP-positive tumour cells expressed significantly lower levels of genes encoding epithelial junction proteins (including CDH1, JUP, CTNN1A) epithelial differentiation markers, including several keratins (6B, 7, 17, 19, 23) and mucins (1, 3A, 4, 12), and chemokines (CXCL1-3, 5, 6) (Supplementary Fig. S4B and Supplementary Table S3). Together the data indicate partial loss of epithelial identity and cell-cell contact in FAP-expressing tumour cells, but no clear gain in expression of mesenchymal genes, which is in line with a ‘partial EMT’ [31].

### Peritoneal metastases express uniformly high levels of FAP

We recently demonstrated that peritoneal metastases from CRC constitute an almost homogeneous entity of CMS4 tumours [18, 32]. Due to the small size of individual tumour nodules, the detection of peritoneal metastases by routine imaging is extremely challenging, hampering patient selection for surgery and assessment of treatment response [33, 34]. To assess whether FAP could function as a potential target for molecular imaging of peritoneal metastases, we measured FAP mRNA and protein expression in a cohort of primary CRC tumours with paired peritoneal metastases.

**Fig. 4:**
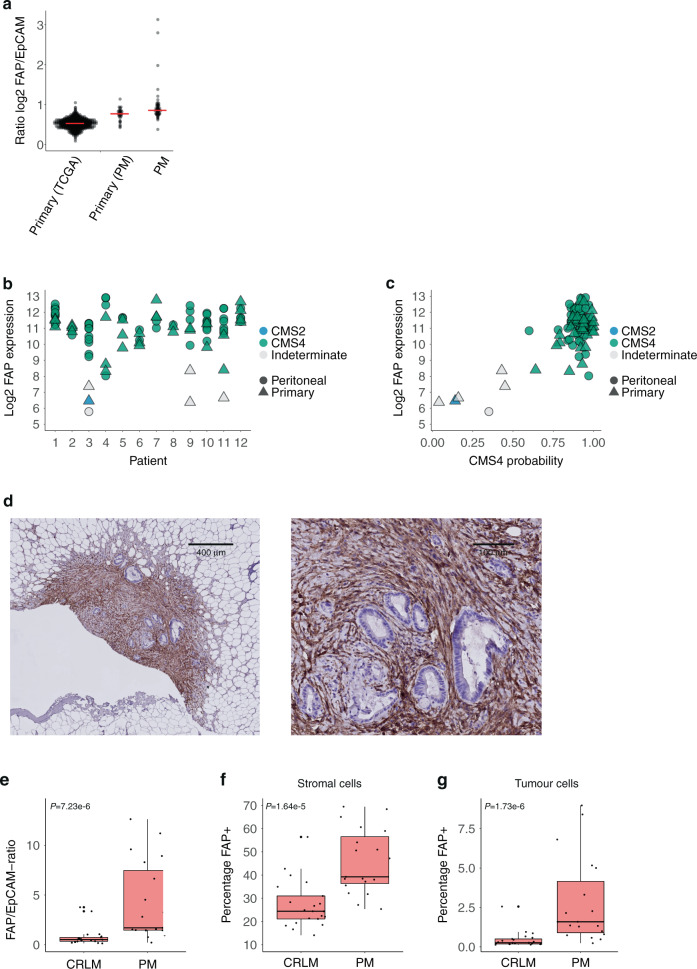
CMS4 Peritoneal Metastases uniformly express high levels of FAP. **a** Dot plot showing relative FAP expression levels in primary CRC tumours in the TCGA cohort (*n* = 592) versus primary tumours with peritoneal involvement (*n* = 35) and the peritoneal metastases derived from them (*n* = 59). **b** Dot plot showing FAP expression levels in peritoneal metastases and their matched primary tumours. CMS subtypes are colour-coded. Tissue type is annotated by shape. **c** Scatter plot showing the correlation of FAP mRNA levels with CMS4 probabilities of all tumours in the primary tumour/peritoneal metastasis cohort. **d** Immunohistochemistry for FAP expression in peritoneal metastasis. See Supplementary Fig. S5 for a comprehensive overview of FAP protein expression in peritoneal metastases. **e** Boxplot of FAP expression quantification on IHC slides of colorectal liver metastasis (CRLM, *n* = 21) and peritoneal metastasis (PM, *n* = 19). FAP expression is measured as the number of FAP-positive cells and corrected for tumour size using an EpCAM staining of the sequential slide. **f** Boxplot of the percentage of FAP-positive stromal cells of colorectal liver metastasis (CRLM, *n* = 21) and peritoneal metastasis (PM, *n* = 19). **g** Boxplot of the percentage of FAP-positive tumour cells of colorectal liver metastasis (CRLM, *n* = 21) and peritoneal metastasis (PM, *n* = 19).

Both primary tumours with peritoneal metastases involvement (*n* = 35 regions from 12 patients), and the peritoneal metastases derived from them (*n* = 59), displayed significantly higher expression levels of FAP when compared to primary CRC tumours in the TCGA cohort (*n* = 582) (Fig. 4a). Moreover, the expression of FAP in peritoneal metastases was even higher than that in the corresponding primary tumours (Fig. 4a). Despite the fact that FAP expression was relatively high across all peritoneal metastases samples, we noted considerable variation in FAP expression among lesions (Fig. 4b). However—also in this high expression range—FAP mRNA levels correlated extremely well with CMS4 probability of the same lesions (Fig. 4c). We conclude that peritoneal metastases constitute an extreme form of CMS4, expressing very high levels of FAP mRNA.

Next, we assessed FAP protein expression in peritoneal metastases from CRC. Immunohistochemistry of 24 peritoneal metastases from 12 individual patients revealed clearly detectable FAP expression in 90% (22/24) of the peritoneal metastases examined (Fig. 4d and Supplementary Fig. S5A). Only 1 metastasis contained identifiable tumour cells without clear FAP expression.

We quantified FAP expression by immunohistochemistry on whole tissue sections, using QuPath [20]. We compared FAP expression in the cohort of peritoneal metastases (*n* = 19) with a cohort of colorectal liver metastases (*n* = 21) [5]. When correcting for tumour size the analysis revealed a significantly higher expression of FAP in peritoneal metastases than in liver metastases (median FAP/EpCAM ratio was 1.7 for PM and 0.5 for CRLM, *P* value = 7.23e-06) (Fig. 4e). As expected, the majority of FAP-positive cells were stromal fibroblasts (Fig. 4f and Supplementary Fig. S5C). However, in a subset of tumours, we also detected FAP expression in tumour cells, again demonstrating the existence of mesenchymal-like tumour cells in CMS4 CRC (Fig. 4g and Supplementary Fig. S5B). Interestingly not only the percentage of FAP-positive stromal cells was significantly higher in peritoneal metastases (39.2% vs 24.4%, *P* value = 1.64e-05) but also the percentage of FAP-positive tumour cells (1.5% vs 0.3%, *P* value = 1.73e-06).

### FAPI-PET detects peritoneal metastases

Peritoneal metastases are notoriously difficult to detect by conventional imaging methods. Given the uniformly high expression of FAP in peritoneal metastases (and their almost uniform CMS4 status) we sought to obtain clinical proof-of-concept for their detection by FAPI-PET. A patient presented with abdominal pain, vomiting and constipation, raising the clinical suspicion of ileus. Two years earlier, this patient had been diagnosed with colon cancer with extensive peritoneal metastases (peritoneal cancer index 27). At that time, the patient received standard first-line chemotherapy followed by extensive cytoreductive surgery (CRS) and hyperthermic intraperitoneal chemotherapy (HIPEC). After treatment, the patient had no clinical or radiological signs of recurrence for over a year.

**Fig. 5:**
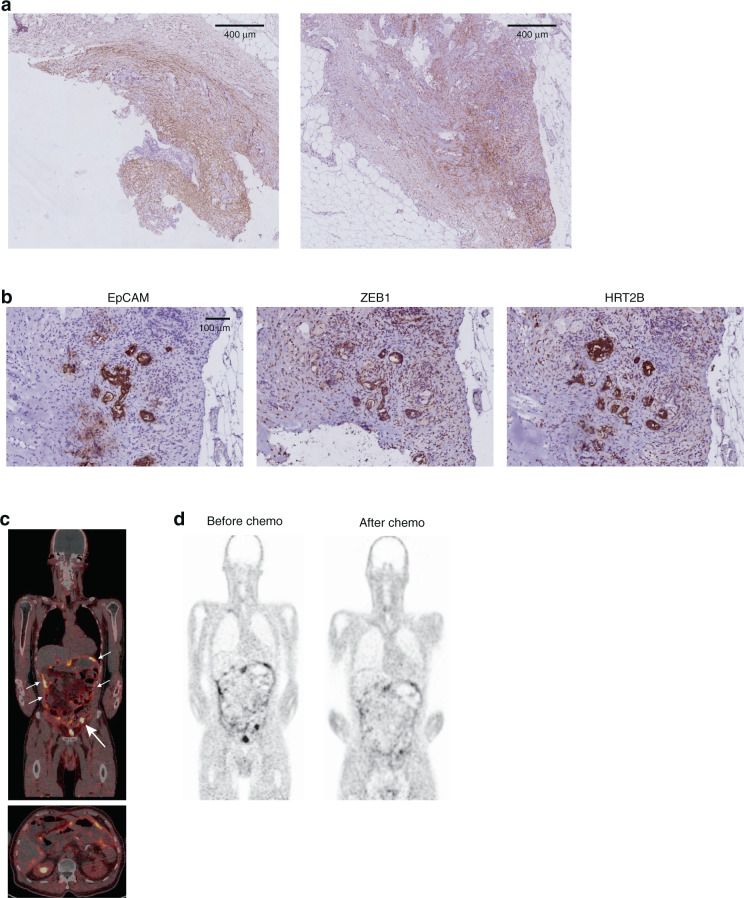
FAPI-PET detects peritoneal metastases. **a** Immunohistochemistry for FAP on peritoneal metastases tissue that was resected one year prior to FAPI-PET imaging. **b** Immunohistochemistry of EpCAM-positive tumour cells in the peritoneum show a strong ZEB1 and HTR2B staining. **c** FAPI-PET image shows tracer accumulation in pelvic peritoneal depositions and along the abdominal peritoneal lining (white arrows). See also Supplementary Fig. S6. **d** FAPI-PET image (same coronal section, same scaling)) after 2 months of chemotherapy shows a reduction of FAPI tracer uptake in the pelvic lesion and the serosal lesion and a complete loss of diffuse FAPI tracer uptake along the peritoneum.

During his recent hospitalisation, repeated CT abdomen and MRI showed mild dilatation of bowel loops but no abrupt calibre change, making a mechanical ileus unlikely. However, a new small peritoneal lesion was identified deep in the pelvis. A differential diagnosis of either recurrent peritoneal metastases, or adhesions after prior surgery, was made. To resolve this diagnostic dilemma, we considered FAPI-PET as an additional imaging modality. First, FAP expression was assessed by immunohistochemistry in the peritoneal metastases that had previously been resected. All resected peritoneal metastases showed nests of tumour cells surrounded by FAP-expressing stroma (Fig. 5a). Interestingly, the tumour cells (identified by EpCAM expression) expressed very high levels of the mesenchymal CRC markers ZEB1 and HTR2B, which can be used to distinguish mesenchymal-like CRC (now commonly referred to as CMS4) from non-mesenchymal CRC [4] (Fig. 5b and Supplementary Fig. S8).

The patient subsequently underwent PET/CT with ^68^Ga-FAPI-46. The FAPI-PET scan showed intense tracer uptake in the pelvic lesion, and in a second serosal lesion ventrally located in the left hemi-abdomen. In addition, diffuse tracer uptake was shown along the peritoneum. Thus, FAPI-PET confirmed recurrent peritoneal metastasis (Fig. 5c). On the same day, contrast-enhanced CT and MRI (using a protocol optimised for peritoneal metastasis detection) were performed, which only identified the focal pelvic lesion, but neither the serosal lesion nor the diffuse peritoneal disease (Supplementary Fig. S6).

The patient subsequently received FOLFIRI-B (fluorouracil, leucovorin, irinotecan, and bevacizumab) systemic therapy and total parenteral nutrition. After 2 months, abdominal pain, vomiting and constipation had resolved and nutritional support was discontinued. Disease evaluation with MRI and ^68^Ga-FAPI-46 PET/CT 2 months after initiation of systemic therapy showed a deep partial response with a reduction of FAPI tracer uptake in the pelvic lesion (SUVlbm-Max: from 5.31 to 3.75 and SUVlbm-Peak from 4.09 to 3.11) and the serosal lesion (SUVlbm-Max from 6.16 to 4.67 and SUVlbm-Peak from 4.91 to 3.96), and a complete loss of diffuse FAPI tracer uptake along the peritoneum (Fig. 5d). The reduction of FAPI tracer uptake following systemic therapy therefore correlated with the observed clinical response. The patient is currently continuing systemic therapy and doing well.

### Heterogeneous FAPI uptake in metastases outside the peritoneum

The majority of distant metastases in CRC occur in the liver (~70%), but only half of these lesions are classified as CMS4 [5, 8]. In normal liver tissue, FAP mRNA was undetectable in 8444 cells from 13 distinct cell types, when analysing single-cell mRNA expression data (Supplementary Fig. S7A, B, ref. [19]). In line with this result, analysis of FAP protein expression by immunohistochemistry showed a complete lack of FAP staining in the normal liver, while it was readily detected in liver metastases (Supplementary Fig. S7C).

**Fig. 6:**
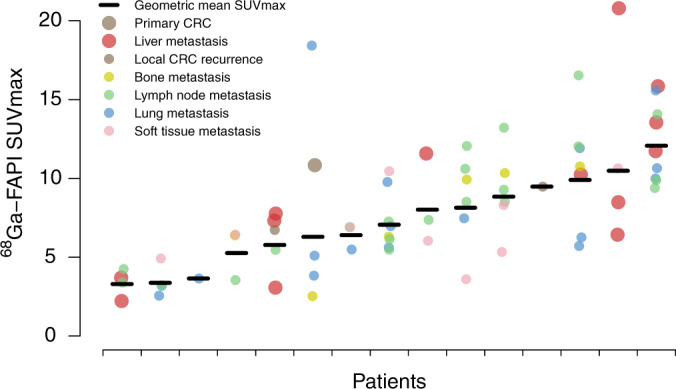
Heterogeneous FAPI uptake in metastases outside the peritoneum. FAPI-PET was performed in 15 CRC patients with distant extra-peritoneal metastases at various sites. The dot plot shows average SUV_max_ values for individual patients. Each dot represents a single lesion and each metastasis site is colour-coded.

FAPI-PET imaging of 15 CRC patients with metastases in the liver, the lungs, and other sites, revealed highly heterogeneous tracer uptake between distinct lesions in individual patients, as well as between patients (Fig. 6a). For instance, maximum standardised uptake values (SUV_max_) differed more than fourfold between three distinct liver metastases in a single patient, and between three distinct lung metastases in another patient (Fig. 6a). However, patients with homogeneously low and homogeneously high tracer uptake across all metastatic lesions were also identified. There was no correlation between extra-peritoneal metastasis site and tracer uptake.

## Discussion

The CMS classification system offers a promising new framework for developing personalised (subtype-targeted) therapeutic strategies. However, the design of such strategies depends on the availability of diagnostic tools that discriminate between the distinct CMS subtypes. For instance, MSI tests identify a CRC subtype that is highly enriched in CMS1 [1], which has consequences for the choice of first-line therapy in metastatic CRC [6]. In the present report, we provide evidence that FAP-binding tracers may serve as a comprehensive diagnostic tool to detect the highly heterogenous CMS4 subtype and distinguish it from CMS1-3. Prior work had already shown that FAP expression in CRC is associated with poor prognosis [35–37] and that FAP is highly expressed in CMS4 [38]. Our work confirms and extends these observations by demonstrating the power of FAP to distinguish CMS4 from CMS1-3 (AUC = 0.91) and by providing clinical proof-of-concept for the detection of a CMS4 tumour by FAPI-PET. The sensitivity and specificity of FAPI-PET for the detection of CMS4 CRC now needs to be determined in clinical studies in which FAPI scans are followed by molecular subtyping of the same surgically resected tumour lesions. As FAP expression is not only high in CMS4, but also in a subgroup of CMS1, it will be important to assess the discriminative ability of FAPI-PET between these two CRC subtypes. Since the vast majority of CMS1 are MSI-high, and MSI testing is routinely performed in the diagnostic work-up of CRC we propose that a combination of FAPI-PET and MSI testing is likely to yield the best discriminative power between CMS1 and CMS4 CRC. Further development of CMS4-targeted therapy requires the identification of effective CMS4-targeting drugs, and the determination of an optimal FAPI-PET signal threshold for patient selection.

The application of FAPI-PET as a CMS4 test has several theoretical advantages over tissue-based CMS4 diagnostic tests. First, it provides whole-body information on CMS4 load, while tissue-based-tests provide information that is limited to one or a few regions of a single lesion. This is important because CMS4 status can vary among and within tumours [5, 8, 10, 11]. Second, the non-invasive nature of PET scans in general allows longitudinal analysis of tumour growth and response to therapy. This would be very challenging with tissue-based diagnostic tools, and impossible in a whole-body multi-lesion manner. Third, FAPI-PET can be implemented in any hospital with a PET facility, thus allowing for rapid worldwide standardisation of CMS4 testing. In this study, we have labelled the FAPI tracer on-site with ^68^Ga. However, centralised production of ^18^F-labelled FAPI would allow its distribution on a national or regional scale, which would contribute to widespread application and standardisation of CMS4 diagnosis.

The specific recognition of CMS4 by FAPI-PET is based on FAP expression, predominantly in myofibroblasts, a cell type that is highly enriched in CMS4 tumours [1]. FAP is an endopeptidase and its substrates include MMP-cleaved collagens and α2-antiplasmin [39]. As a result, FAP activity promotes collagen matrix remodelling and fibrosis [39]. The expression of FAP in endothelial cells is interesting as it may help bind and sequester the systemically administered tracer. The relative contribution of endothelial FAP and stromal FAP to tracer binding deserves further analysis, as it may provide insight into the mechanisms of tracer uptake and retention in CMS4 CRC. Interestingly, we and others [35] have also identified FAP expression in a subset of tumour cells. The contribution of FAP + tumour cells to tracer retention is likely to be small. However, from a cancer biology perspective, this finding is very relevant because it demonstrates that mesenchymal-like tumour cells exist in cancer patients, and can be targeted through FAP. We recently demonstrated that mesenchymal-like tumour cells are the instigators of distant metastasis in models of CRC [31]. Others found that FAP stimulates tumour cell invasion and metastasis in a tumour cell-intrinsic manner [40]. The development of strategies eliminating metastasis-initiating cells is regarded as one of the holy grails in cancer research [41]. The development of FAPI as a theranostic tool would theoretically enable the ‘search-and-destroy’ of these metastasis-initiating cells: ^131^I-labelled or ^90^Y-labelled FAPI could not only target the FAP-positive tumour bulk (through its endothelium and stroma), but possibly also disseminated FAP-positive metastasis-initiating tumour cell populations. Of note, two case studies have demonstrated proof-of-concept for the potential benefit of FAP-targeted radiotherapy (^90^Y-FAPI), including the disappearance of colorectal PM [42, 43].

In the accompanying paper [18], we have identified peritoneal metastases as a near-homogeneous CMS4 entity within CRC. The detection of peritoneal metastases in CRC is a major clinical challenge [33, 34]. Underestimation of intraperitoneal tumour load based on laparoscopic exploration and/or conventional imaging leads to discontinuation of potentially curative surgery in up to 40% of the cases [44, 45]. Moreover, the response of peritoneal metastases to systemic therapy is difficult to assess using conventional imaging, often leading to the exclusion of patients with peritoneal metastases from clinical studies [46]. Diffusion-weighted MRI (DW-MRI) has the highest sensitivity for detecting peritoneal metastases and has recently been put forward as the method of choice for their imaging-based diagnosis [34, 47, 48]. Based on the high expression of FAP in virtually all peritoneal metastases, we propose that FAPI-PET may be an alternative promising imaging modality for the detection of peritoneal metastases. Recent clinical data indicate that FAPI-PET is superior over FDG-PET in detecting primary tumours and metastases—including peritoneal metastases—from multiple cancer types including CRC [49, 50]. Clinical studies comparing the golden standard of peritoneal metastasis detection (laparoscopic exploration and/or DW-MRI) with FAPI-PET are now needed to determine the best strategy for future diagnosis of peritoneal metastases.

Strikingly, we observed a correlation between the clinical response to systemic treatment and a concomitant reduction of FAPI tracer uptake in peritoneal lesions. The value of FAPI-PET as a method for response evaluation of peritoneal metastases should come from studies in which changes in tracer signal are compared with tissue-based cellular, histopathological and molecular analyses, for instance in patients who are candidates for cytoreductive surgery and have received prior systemic therapy. Alternatively, longitudinal biopsy-based analysis of target lesions is feasible in patients receiving multiple cycles of intra-abdominal chemotherapy for inoperable peritoneal metastases [51, 52]. The analysis of FAPI tracer uptake before and after chemotherapy coupled to histopathological tissue analysis is needed to assess the sensitivity and specificity of FAPI-PET as a diagnostic tool for intraperitoneal response monitoring.

The majority (~70%) of distant metastases in CRC occur in the liver. Approximately half of all liver metastases are classified as ‘mesenchymal-like’ tumours or CMS4 [5, 8]. Novel systemic CMS4-targeted therapies need to be tested in pre-selected patient groups with CMS4 liver metastases. Since the molecular classification of primary tumours and matched liver metastases is discordant in approximately half of the cases [5, 8], resected primary tumours cannot be used for patient selection. We propose that FAPI-PET may offer a solution to this problem, as it comprehensively identifies FAP-rich stroma (and thereby ‘CMS4 load’) in all target lesions, without the need to infer subtype from histological analysis of the primary tumour, and without the need for additional invasive biopsy procedures. While background signals in the normal liver, lungs and peritoneum are very low [53], benign lesions may show tracer uptake [16]. In some cases, this may lead to an overestimation of (metastatic) tumour load. Future studies should therefore compare FAPI-PET signals with tissue-based histopathological, molecular and cellular parameters of tumour biology, including CMS status.

In conclusion, our work identifies FAP as an excellent marker of CMS4 CRC, including peritoneal metastases, and suggests that FAPI-PET is a promising tool for its comprehensive detection, thus generating a quantitative measure of ‘CMS4-load’ in cancer patients, which may form a basis for patient selection. Furthermore, FAPI-PET could be rapidly implemented in most hospitals worldwide. Further development of FAPI-PET as a CMS4-identifying diagnostic tool will require assessment of its sensitivity and specificity in multiple organ microenvironments.

### Reporting summary

Further information on research design is available in the Nature Research Reporting Summary linked to this article.

## Supplementary information

Supplemental figures

Table S1

Table S2

Table S3

checklist reporting summary

## Data Availability

The datasets used and/or analysed during this study are available from the corresponding author on reasonable request.
