# Supplementary material for: Fibroblast activation protein identifies Consensus Molecular Subtype 4 in colorectal cancer and allows its detection by 68Ga-FAPI-PET imaging
Source: Br J Cancer. 2022 Mar 16;127(1):145–55. doi: 10.1038/s41416-022-01748-z (PMC9276750; doi:10.1038/s41416-022-01748-z)
Supplement: Supplementary file 1 — Supplemental figures [file 41416_2022_1748_MOESM1_ESM.pdf]

Supplemental Figures 1-8

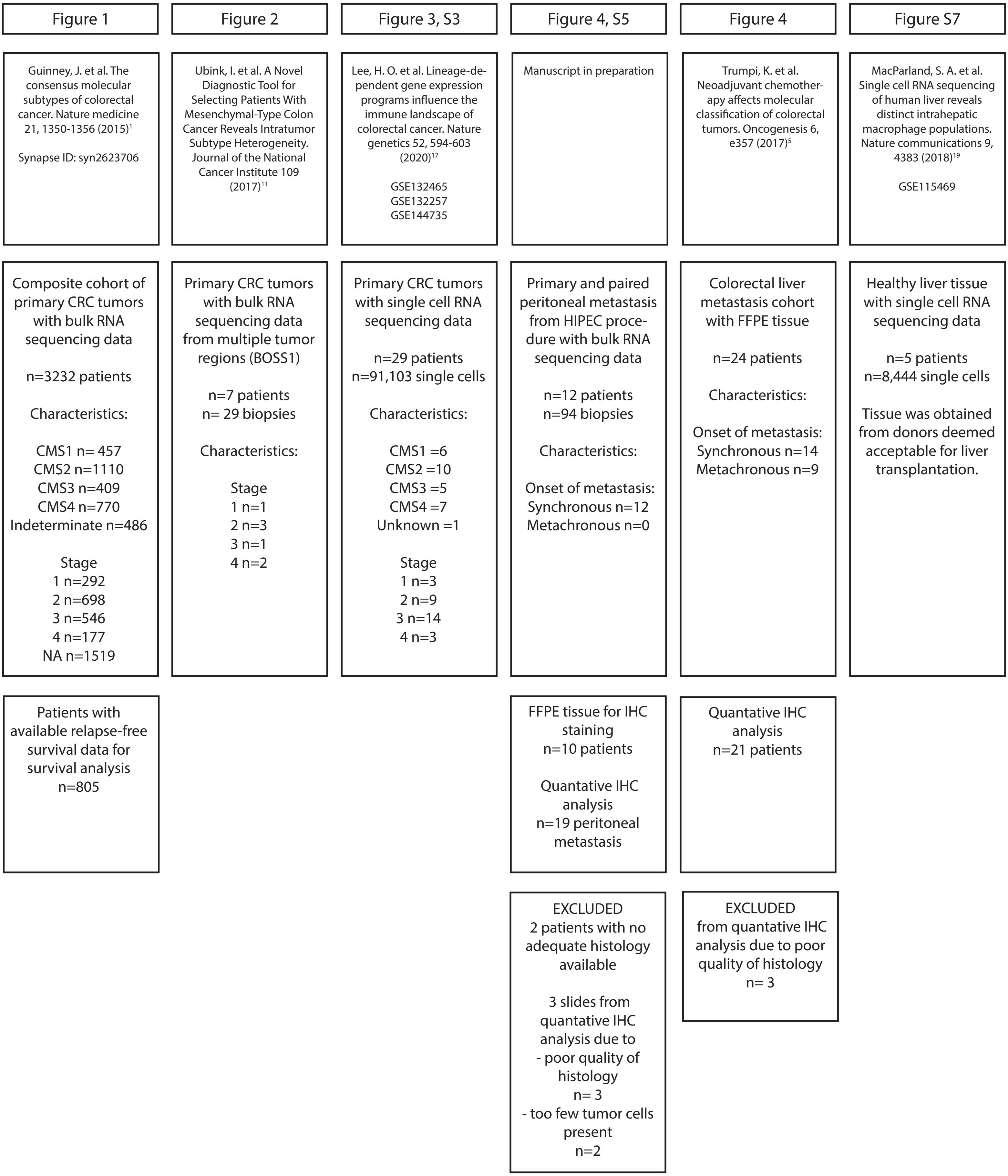

Figure S1. Overview of study cohorts.

A

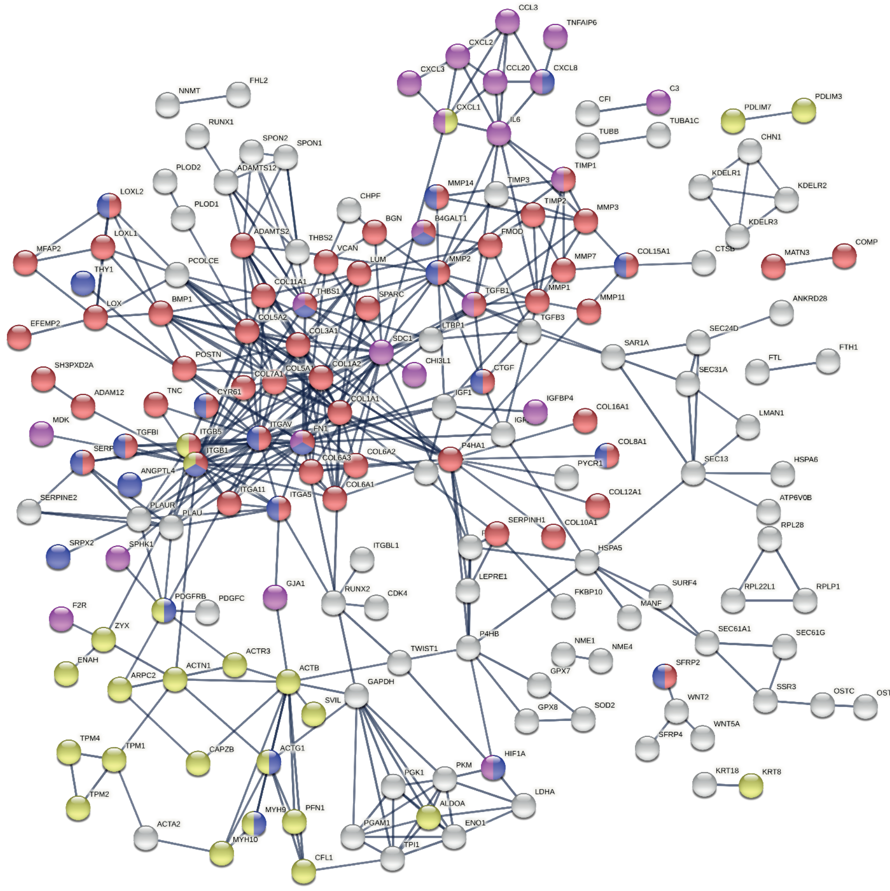

B

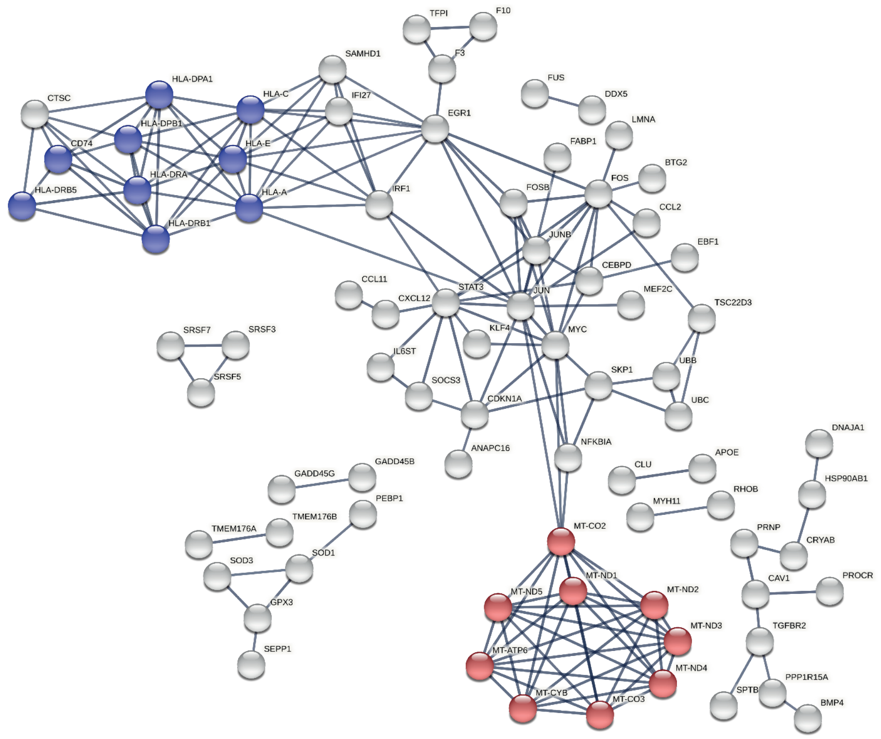

Figure S2. FAP-expressing stromal cells display a profibrotic phenotype. a. STRING protein network analysis was used to generate interaction maps (highest confidence) of genes expressed at significantly higher levels in FAP-positive stromal cells than in FAP-negative stromal cells. Disconnected nodes are not shown. The following gene ontology terms are color-coded: Red: Extracellular Matrix Organization. Purple: Inflammatory response. Yellow: Actin cytoskeleton organization. Blue: Angiogenesis. See Table S2 for all fold-changes and accompanying p-values. b. As in A, but for genes that are expressed at significantly lower levels in FAP-positive stromal cells than in FAP-negative stromal cells. The following gene ontology terms are color-coded: Blue: Antigen processing and presentation. Red: Oxidative Phosphorylation. See Table S2 for all fold-changes and accompanying p-values.

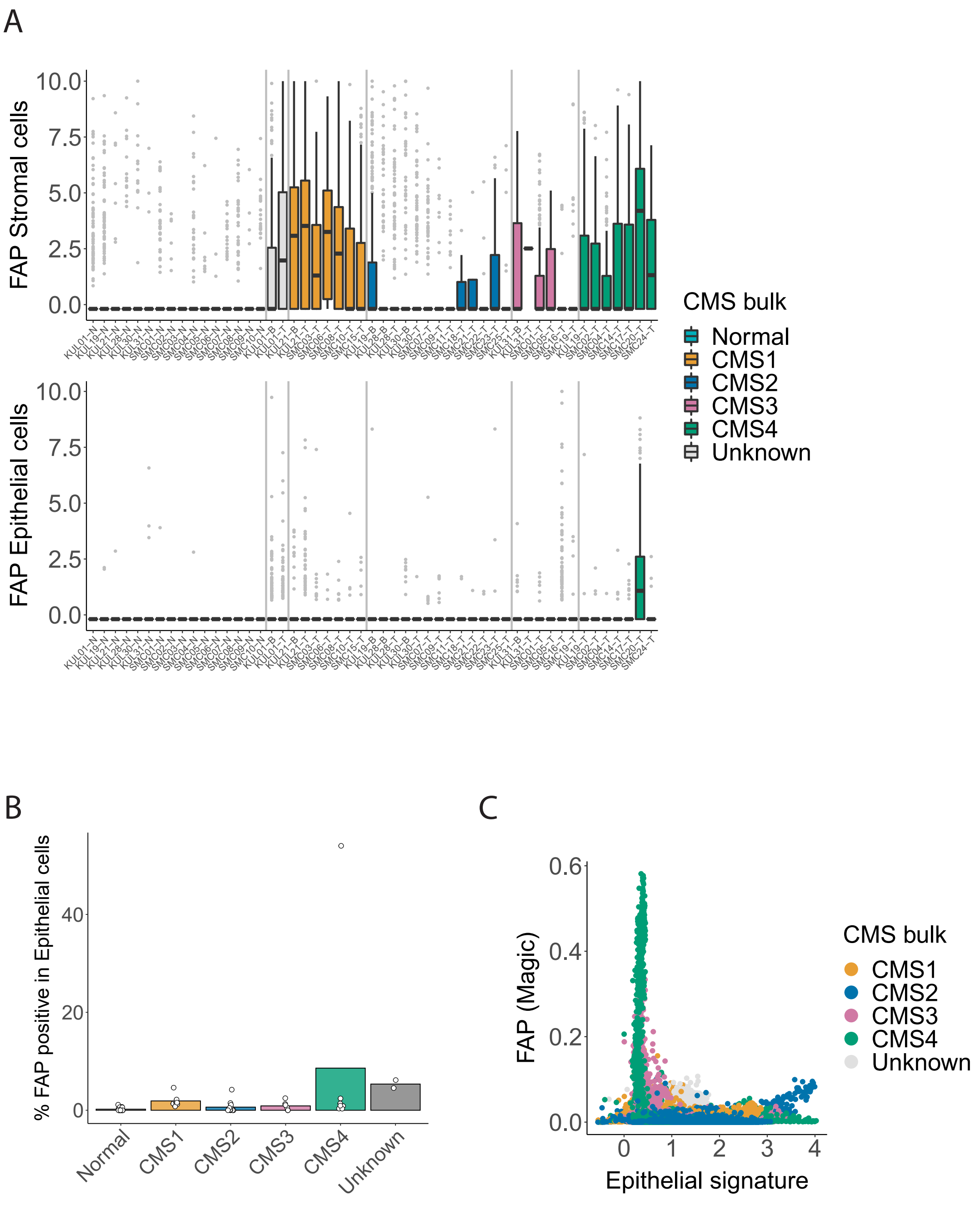

Figure S3. Distribution of FAP-expressing cell types in relation to tumor CMS classi¬fication. a. Box dot plots showing FAP expression in stromal cells (top) and epithelial cells (bottom), according to CMS subtype determined by analysis of bulk tumor tissue. b. Bar chart of mean percentage of FAP positive epithelial cells according to CMS subtype. Dots are datapoints of individual tumors. c. Scatter plot showing the anti-correlation between FAP expression in single tumor cells with a signature reflecting epithelial differentiation (17). CMS status of the corresponding tumors is color-coded.

A

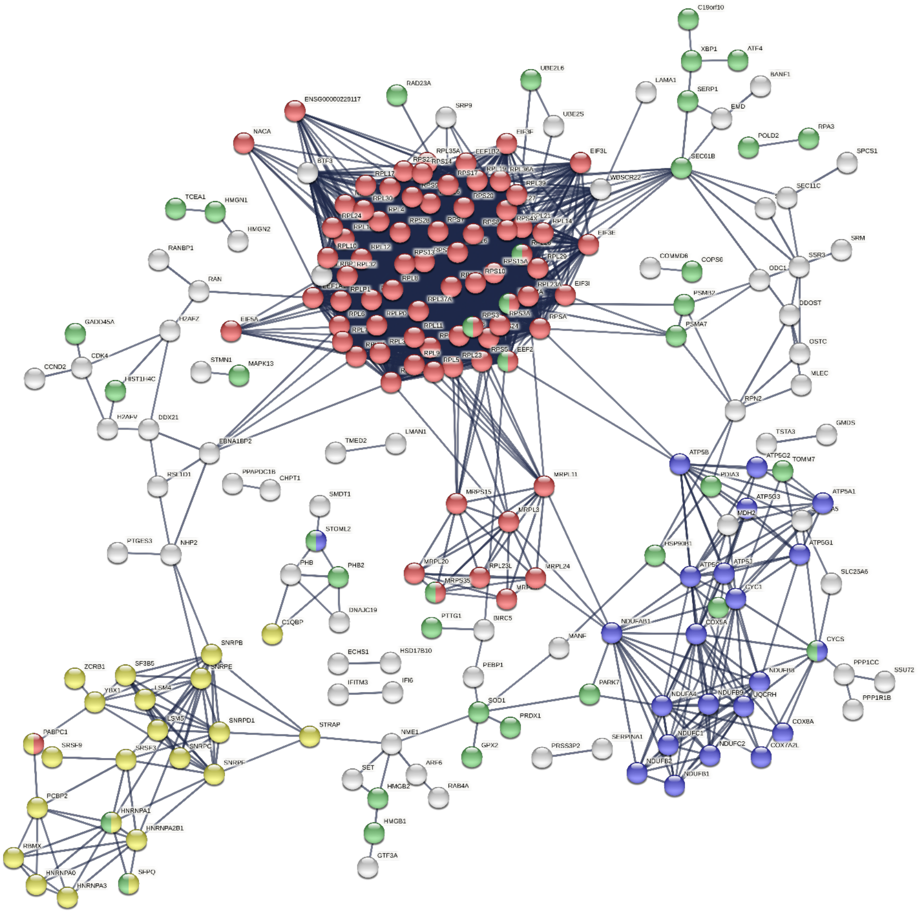

B

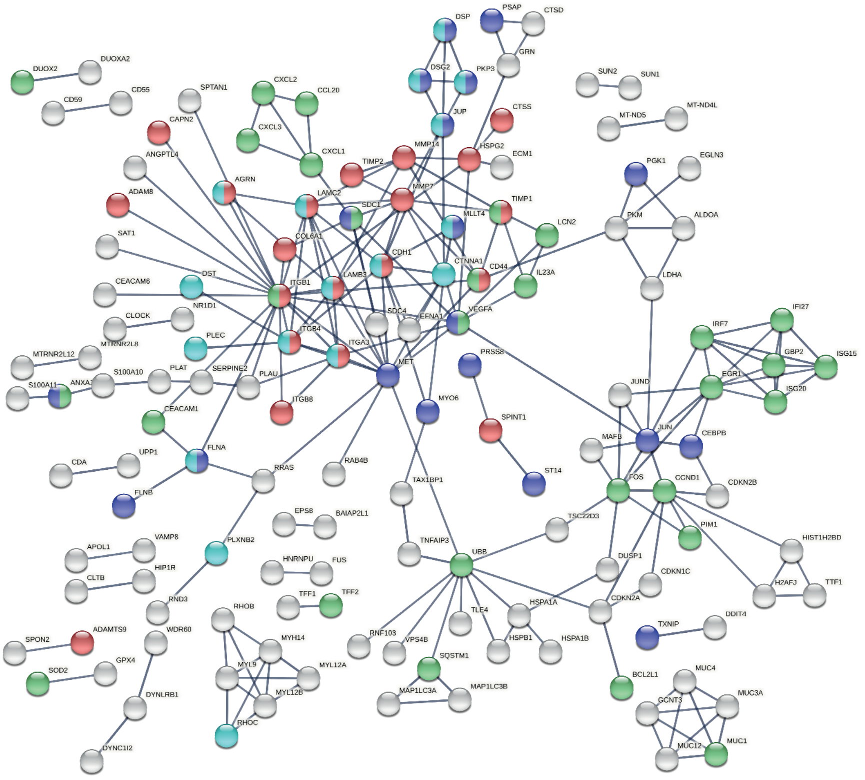

Figure S4. FAP-expressing tumor cells display features of a "partial EMT". a. STRING protein network analysis was used to generate interaction maps (highest confidence) of genes expressed at significantly higher levels in FAP-positive epithelial tumor cells from patient SMC20 than in FAP-negative tumor cells. Disconnected nodes are not shown. The following gene ontology terms are color-coded: Red: protein translation. Purple: Inflammatory response. Yellow: RNA splicing. Blue: Oxidative phosphorylation. Green: cellular response to stress. See Table S3 for all fold-changes and accompanying p-values. b. As in A, but for genes that are expressed at significantly lower levels in FAP-positive tumor cells than in FAP-negative tumor cells. The following gene ontology terms are color-coded: Dark blue: Epithelial cell differentiation. Light Blue: Cell Junction organization. Green: Cytokine-mediated signaling. Red: Extracellular matrix organization.

A

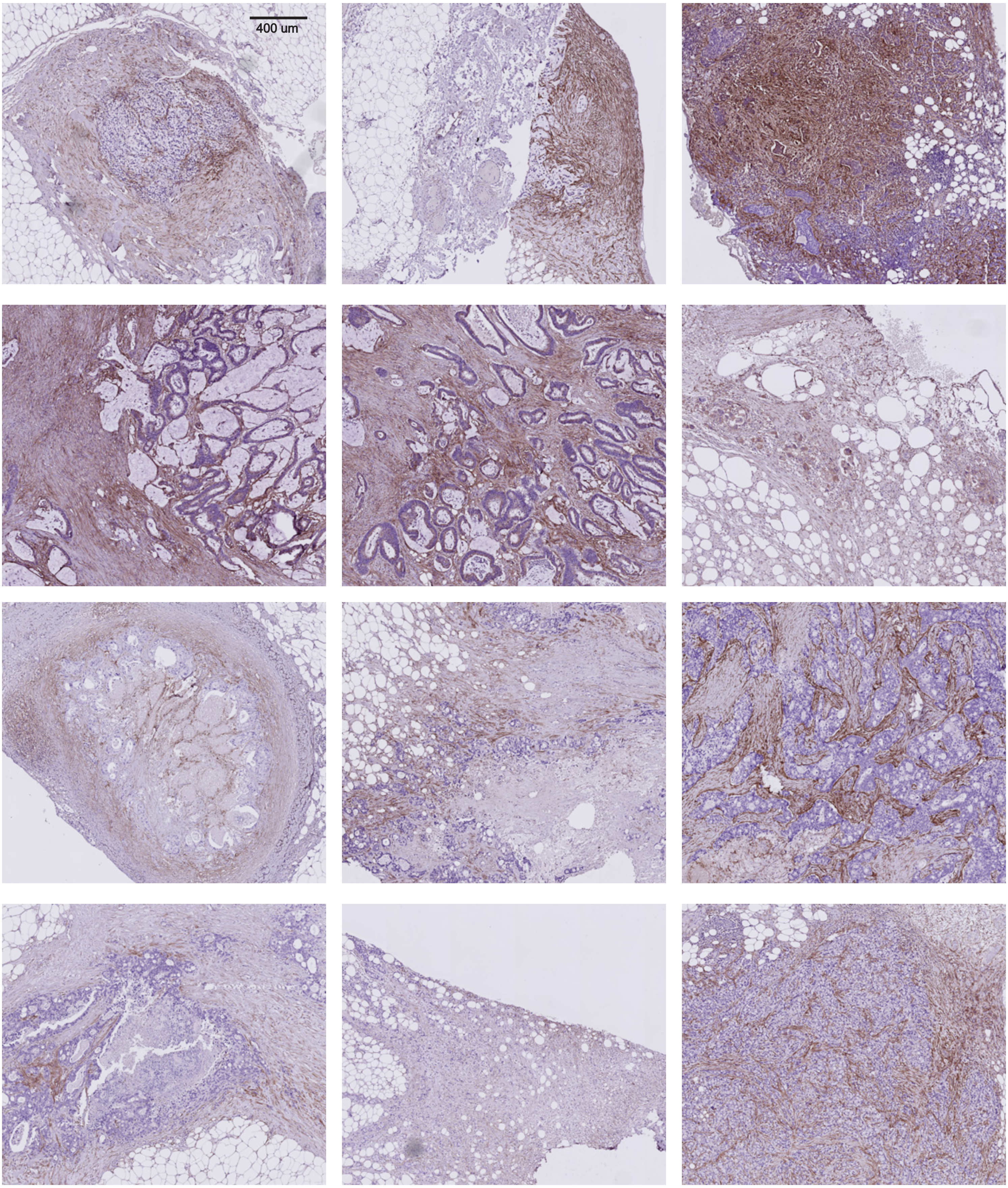

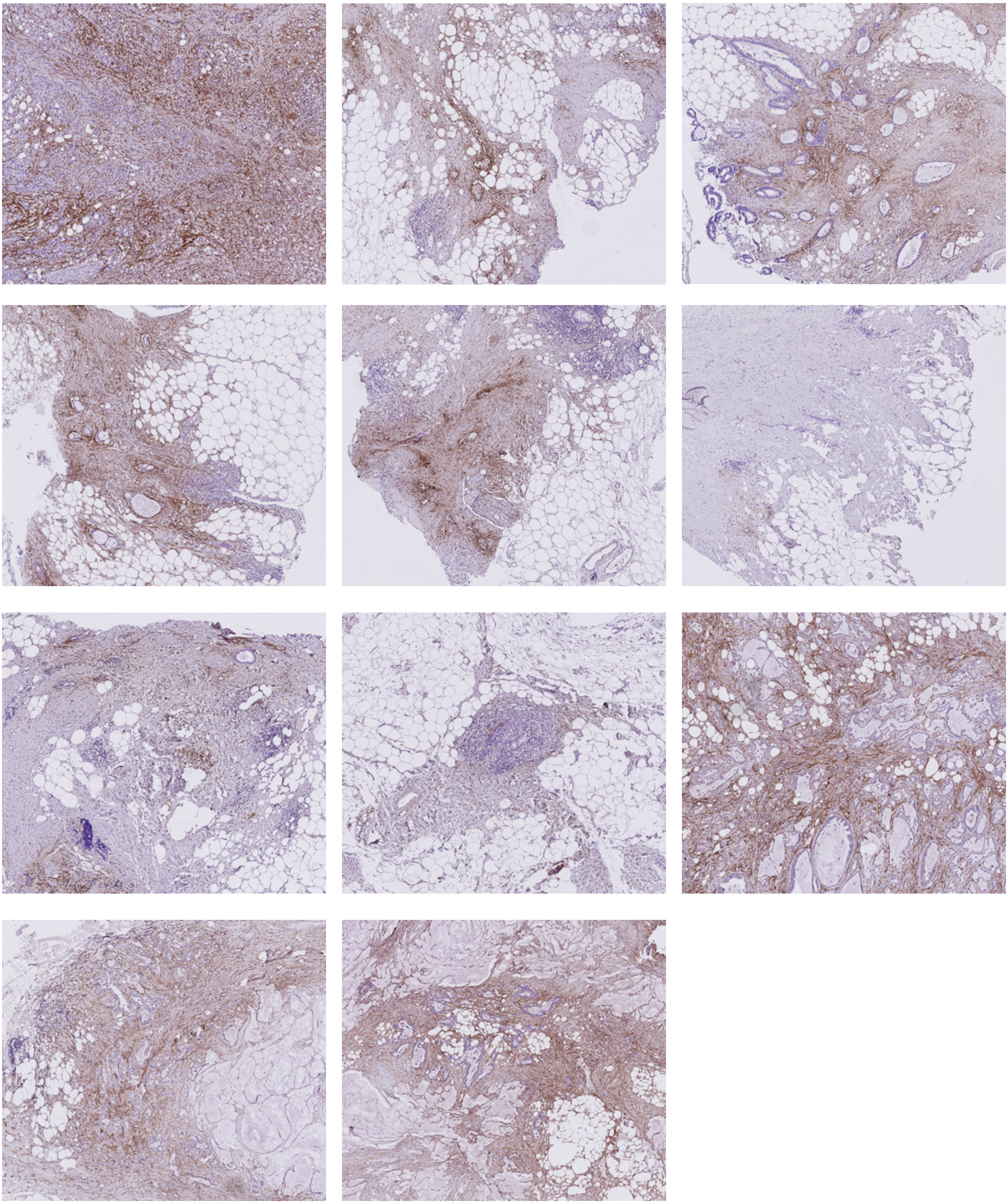

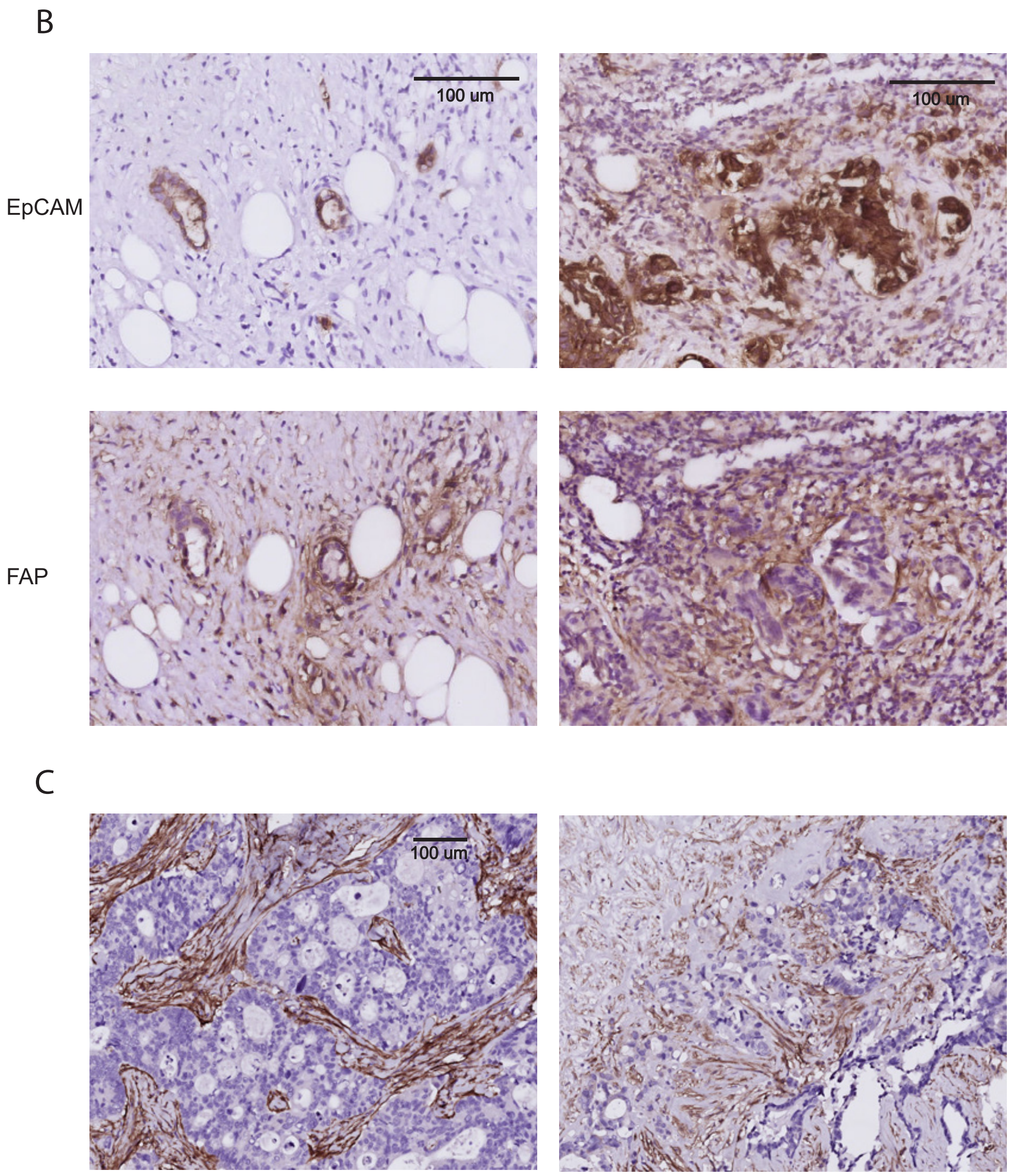

Figure S5. CMS4 Peritoneal Metastases uniformly express high levels of FAP. a. FAP immunohistochemistry on 23 peritoneal metastases. b. Zoomed images showing EpCAM and FAP staining of tumor cells. c. Zoomed images showing FAP staining of stromal fibroblasts.See Table S3 for all fold-changes and accompanying p-values.

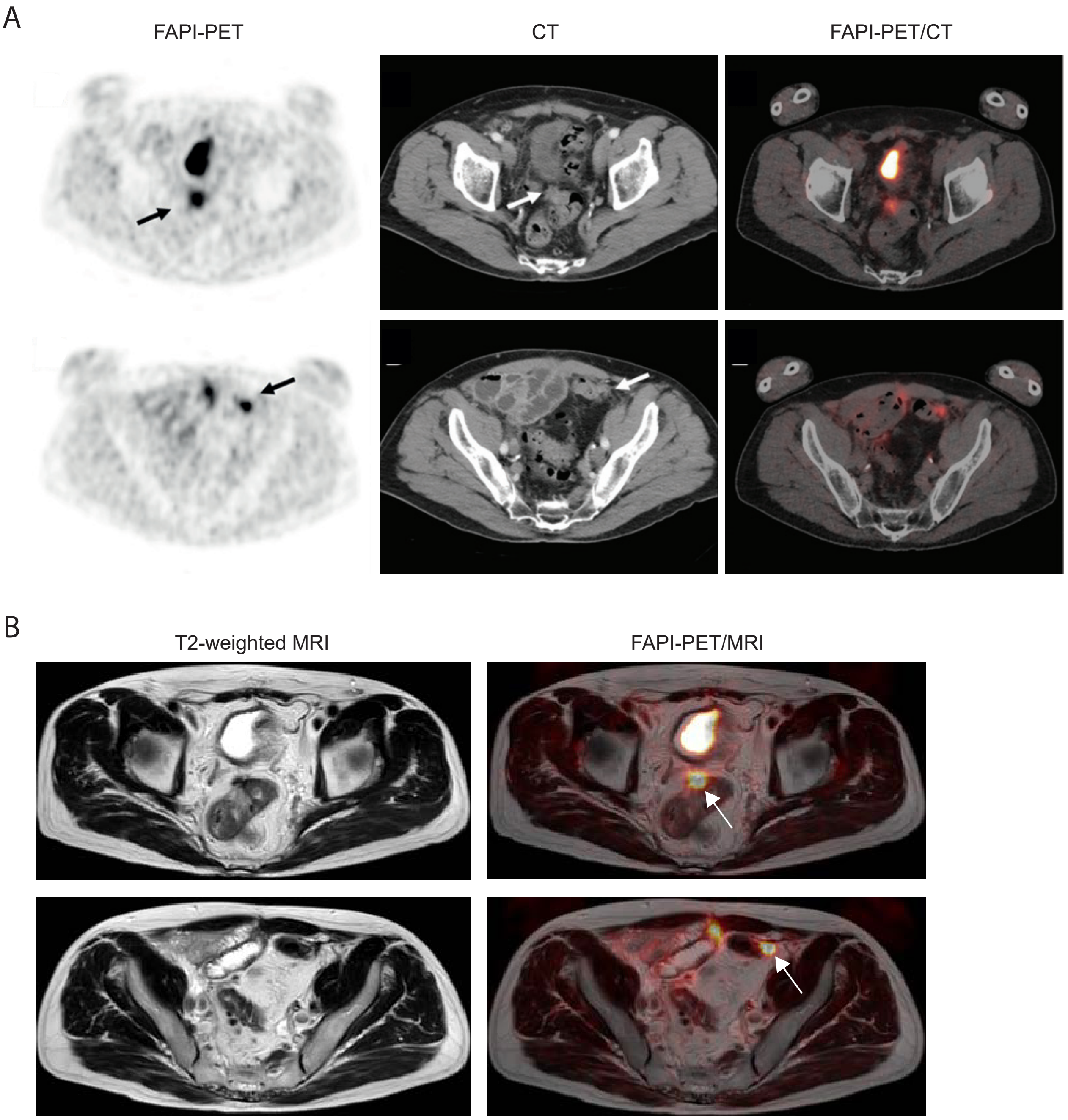

Figure S6. FAPI PET detects peritoneal metastases that are not visible using conventional imaging. a. Axial PET, CT, and fusion images show tracer retention in focal peritoneal metastatic lesions in the pelvis. The lesion adjacent to the sigmoid colon (upper image row) was the only lesion identified on CT and MRI. The ventrally located serosal lesion in the left hemi-abdomen (lower image row), was not recognized on CT or MRI. b. Axial T2-weighted MRI images and FAPI-PET-MRI fusion images showing the same lesions, indicated by the arrows.

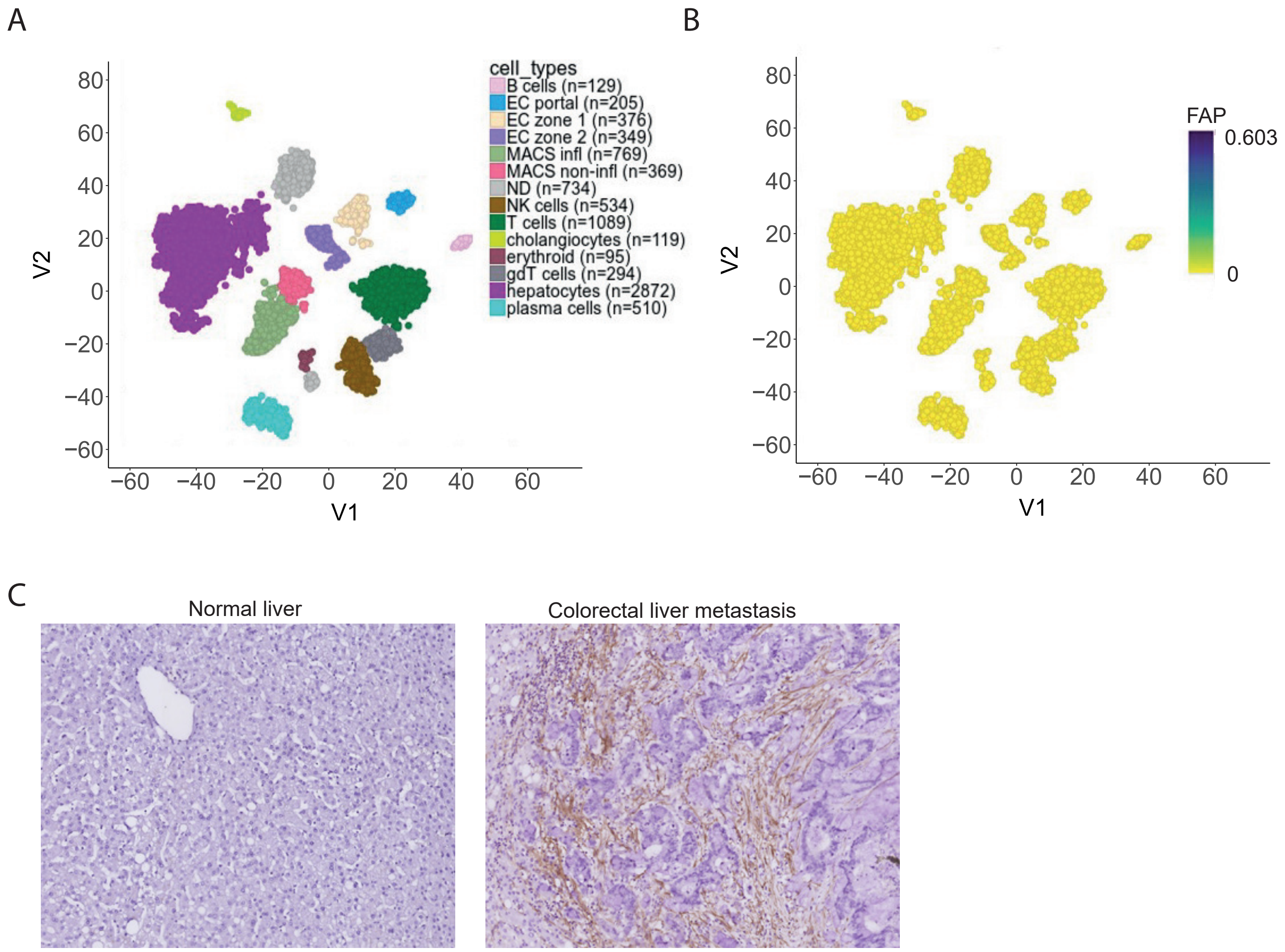

Figure S7. FAP is not expressed in the normal liver. a. tSNE plot showing 8444 cells from 13 distinct color-coded cell types that are present in the normal liver (22). b. The same tSNE plot as in (A), with FAP mRNA levels color-coded. Yellow indicates zero read counts. FAP expression was undetectable in all cells. c. Immunohistochemistry analysis of FAP protein expression in the normal liver (left panel) and in a liver metastasis (right panel).

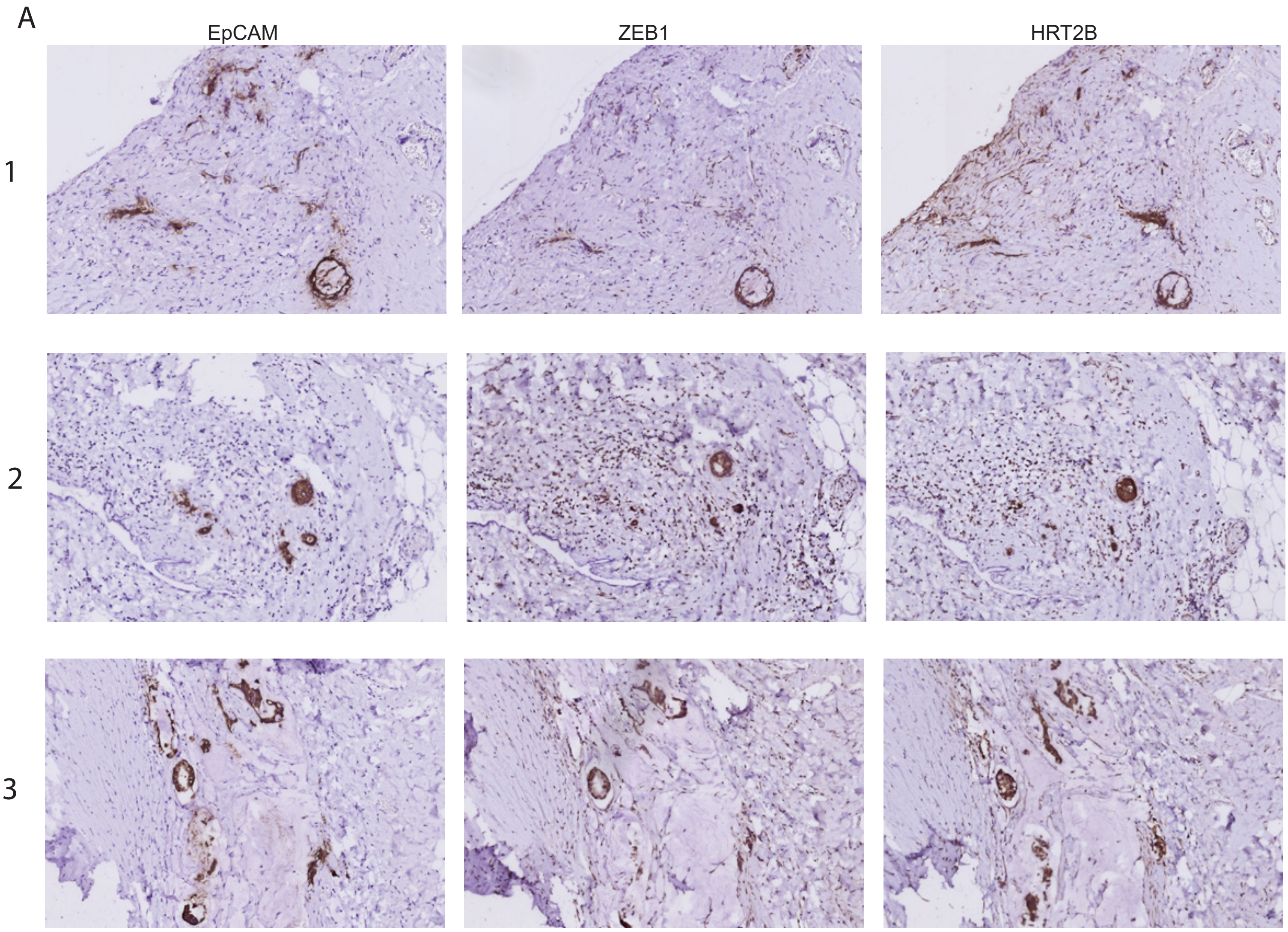

Figure S8. Peritoneal metastasis express high levels of ZEB1 and HTR2B. Three distinct peritoneal metastasis were stained for EpCAM, ZEB1 and HTR2B.
