# Supplementary material for: Fibroblast activation protein identifies Consensus Molecular Subtype 4 in colorectal cancer and allows its detection by 68Ga-FAPI-PET imaging
Source: Br J Cancer. 2022 Mar 16;127(1):145–55. doi: 10.1038/s41416-022-01748-z (PMC9276750; doi:10.1038/s41416-022-01748-z)
Supplement: Supplementary file 2 — Table S1 [file 41416_2022_1748_MOESM2_ESM.docx]

| **Cell surface proteins higher in CMS4** | | | |  | | **Cell surface proteins higher in CMS2** | | | |
| --- | --- | --- | --- | --- | --- | --- | --- | --- | --- |
| **hugo** | **corrected-P** | **Log2 fold-change** |  | | **hugo** | | **corrected-P** | **Log2 fold-change** |  |
| COL6A2 | 0,00E+00 | 1,22 |  | | SLC5A6 | | 2,32E-130 | -1,10 |  |
| HTRA1 | 0,00E+00 | 1,21 |  | | SRPK1 | | 9,10E-126 | -1,08 |  |
| GAS1 | 0,00E+00 | 1,64 |  | | UBE2C | | 1,38E-124 | -1,13 |  |
| PTRF | 0,00E+00 | 1,17 |  | | TSPAN6 | | 1,76E-118 | -1,10 |  |
| **FAP** | **5,92E-319** | **1,39** |  | | DNAJA3 | | 1,70E-116 | -1,07 |  |
| BGN | 3,00E-317 | 1,26 |  | | HSPD1 | | 3,01E-112 | -1,05 |  |
| FRMD6 | 1,05E-315 | 1,33 |  | | RNF43 | | 7,35E-109 | -1,13 |  |
| CNRIP1 | 2,10E-314 | 1,24 |  | | ATIC | | 6,32E-107 | -1,05 |  |
| MRC2 | 4,41E-310 | 1,16 |  | | EPB41L4B | | 1,57E-101 | -1,12 |  |
| PLEKHO1 | 3,94E-306 | 1,20 |  | | TMEM97 | | 6,11E-101 | -1,10 |  |
| DDR2 | 2,56E-305 | 1,31 |  | | SPIRE2 | | 1,26E-100 | -1,10 |  |
| RAB31 | 4,87E-305 | 1,24 |  | | AXIN2 | | 9,29E-98 | -1,14 |  |
| SPARC | 8,98E-301 | 1,16 |  | | RAB11FIP4 | | 1,06E-97 | -1,10 |  |
| PALLD | 3,32E-300 | 1,17 |  | | PIGU | | 7,63E-95 | -1,08 |  |
| EHD2 | 1,81E-297 | 1,16 |  | | MAP7 | | 1,34E-94 | -1,08 |  |
| PMP22 | 8,09E-294 | 1,19 |  | | BRCA1 | | 1,36E-94 | -1,12 |  |
| FGFR1 | 5,46E-291 | 1,20 |  | | EPHB2 | | 2,37E-93 | -1,09 |  |
| COLEC12 | 8,27E-291 | 1,49 |  | | PTCD3 | | 9,44E-92 | -1,06 |  |
| AMOTL1 | 9,42E-289 | 1,25 |  | | SUCLG1 | | 3,28E-91 | -1,06 |  |
| ANTXR1 | 4,47E-286 | 1,22 |  | | CLCN2 | | 3,69E-91 | -1,10 |  |
| KIAA1462 | 3,22E-285 | 1,30 |  | | FGFR4 | | 7,05E-91 | -1,11 |  |
| BASP1 | 1,14E-283 | 1,23 |  | | B3GNT3 | | 4,49E-89 | -1,08 |  |
| IL1R1 | 5,20E-283 | 1,24 |  | | PHB | | 1,28E-88 | -1,07 |  |
| SLIT2 | 2,40E-282 | 1,48 |  | | CFTR | | 3,29E-88 | -1,16 |  |
| ITGA5 | 2,26E-280 | 1,19 |  | | GPR35 | | 1,57E-87 | -1,09 |  |
| COL6A1 | 5,62E-280 | 1,18 |  | | VIL1 | | 4,54E-86 | -1,10 |  |
| PRKCDBP | 2,86E-276 | 1,23 |  | | PLK4 | | 1,86E-85 | -1,13 |  |
| CALD1 | 5,37E-276 | 1,17 |  | | CDH1 | | 3,15E-85 | -1,07 |  |
| BOC | 5,88E-275 | 1,28 |  | | ANKS4B | | 9,51E-85 | -1,11 |  |
| FERMT2 | 8,05E-272 | 1,27 |  | | ABCC6 | | 1,50E-84 | -1,17 |  |
| MSN | 1,17E-271 | 1,15 |  | | EPB41L5 | | 3,00E-84 | -1,12 |  |
| MRAS | 9,28E-271 | 1,20 |  | | VAV3 | | 3,33E-83 | -1,15 |  |
| COL6A3 | 1,33E-269 | 1,17 |  | | INPP5J | | 3,93E-82 | -1,09 |  |
| EVC | 2,32E-268 | 1,25 |  | | STX3 | | 7,97E-82 | -1,08 |  |
| MAP1B | 2,80E-268 | 1,30 |  | | PTPN3 | | 1,20E-80 | -1,08 |  |
| ATP8B2 | 2,55E-267 | 1,25 |  | | ANO9 | | 3,83E-80 | -1,10 |  |
| GPNMB | 4,05E-266 | 1,31 |  | | PARD6B | | 3,95E-80 | -1,13 |  |
| THY1 | 9,95E-264 | 1,16 |  | | ERBB3 | | 2,56E-78 | -1,08 |  |
| ITGAM | 3,58E-263 | 1,34 |  | | MARVELD2 | | 3,88E-77 | -1,08 |  |
| MMP2 | 2,26E-262 | 1,19 |  | | ATP10B | | 1,55E-76 | -1,10 |  |
| CACNA2D1 | 1,56E-261 | 1,47 |  | | GJB1 | | 2,32E-76 | -1,09 |  |
| TSPAN4 | 2,00E-260 | 1,16 |  | | ACE2 | | 1,39E-75 | -1,25 |  |
| CDH11 | 1,02E-259 | 1,26 |  | | HMMR | | 6,01E-75 | -1,12 |  |
| CD109 | 1,77E-259 | 1,36 |  | | RACGAP1 | | 8,25E-75 | -1,06 |  |
| FAM129A | 2,34E-258 | 1,32 |  | | GUCY2C | | 1,03E-74 | -1,14 |  |
| NRP2 | 1,90E-255 | 1,19 |  | | PRLR | | 1,65E-74 | -1,17 |  |
| VIM | 3,03E-254 | 1,15 |  | | FBXO45 | | 1,86E-73 | -1,07 |  |
| PRKD1 | 3,05E-254 | 1,35 |  | | IL22RA1 | | 3,69E-73 | -1,10 |  |
| GYPC | 4,69E-252 | 1,18 |  | | IL17RB | | 9,71E-73 | -1,11 |  |
| KCNE4 | 1,01E-251 | 1,30 |  | | SMAGP | | 1,48E-72 | -1,08 |  |
| ADAM12 | 4,66E-250 | 1,40 |  | | EXO1 | | 2,89E-72 | -1,11 |  |
| CDH2 | 1,86E-248 | 1,32 |  | | SLC5A1 | | 6,54E-71 | -1,12 |  |
| SYNE1 | 2,58E-248 | 1,26 |  | | CEP55 | | 1,11E-70 | -1,13 |  |
| DFNA5 | 2,61E-248 | 1,28 |  | | KIAA1804 | | 1,18E-70 | -1,09 |  |
| CYBRD1 | 3,72E-248 | 1,27 |  | | GPR39 | | 2,82E-70 | -1,11 |  |
| AKAP12 | 4,74E-248 | 1,30 |  | | PKP3 | | 4,00E-70 | -1,05 |  |
| FXYD6 | 2,60E-247 | 1,24 |  | | FZD5 | | 7,64E-70 | -1,08 |  |
| SSPN | 1,67E-246 | 1,28 |  | | CXADR | | 4,07E-69 | -1,09 |  |
| PCDH7 | 2,19E-246 | 1,39 |  | | KCNQ1 | | 5,03E-69 | -1,08 |  |
| GGT5 | 7,46E-246 | 1,17 |  | | ARHGEF5 | | 6,89E-69 | -1,05 |  |
| GPC6 | 1,25E-243 | 1,30 |  | | FERMT1 | | 8,23E-69 | -1,09 |  |
| CTGF | 1,53E-243 | 1,19 |  | | GOT2 | | 1,42E-68 | -1,05 |  |
| SPG20 | 4,01E-243 | 1,26 |  | | CDH17 | | 1,80E-68 | -1,10 |  |
| CCDC88A | 8,96E-242 | 1,27 |  | | SLC9A3R1 | | 2,32E-68 | -1,06 |  |
| PHLDB2 | 1,87E-241 | 1,32 |  | | CACNA1D | | 3,33E-67 | -1,15 |  |
| SGCE | 4,24E-241 | 1,29 |  | | SERINC5 | | 8,05E-67 | -1,05 |  |
| FLNA | 6,63E-241 | 1,18 |  | | ZNRF3 | | 2,62E-66 | -1,10 |  |
| FCGR2B | 1,45E-240 | 1,38 |  | | EBP | | 2,69E-66 | -1,06 |  |
| PDGFC | 2,45E-240 | 1,24 |  | | PAQR8 | | 3,83E-66 | -1,09 |  |
| NTM | 1,22E-238 | 1,35 |  | | CASK | | 4,94E-66 | -1,08 |  |
| AOC3 | 4,12E-238 | 1,34 |  | | ZNRF2 | | 6,54E-66 | -1,09 |  |
| EMP3 | 6,10E-238 | 1,19 |  | | SLC38A1 | | 1,29E-65 | -1,06 |  |
| NRP1 | 6,34E-237 | 1,25 |  | | GPR143 | | 1,29E-65 | -1,18 |  |
| CRYAB | 2,29E-236 | 1,27 |  | | ACP1 | | 4,19E-65 | -1,06 |  |
| CDK14 | 1,49E-234 | 1,28 |  | | PLEKHG6 | | 7,95E-65 | -1,07 |  |
| MPDZ | 1,50E-234 | 1,25 |  | | MELK | | 9,93E-65 | -1,11 |  |
| SULF1 | 4,59E-234 | 1,26 |  | | FLVCR1 | | 5,05E-63 | -1,09 |  |
| SPHK1 | 9,27E-233 | 1,21 |  | | CDHR1 | | 9,05E-63 | -1,19 |  |
| FEZ1 | 9,88E-233 | 1,26 |  | | GPSM2 | | 1,60E-62 | -1,10 |  |
| CLIP3 | 1,05E-232 | 1,17 |  | | KCNK5 | | 3,99E-62 | -1,10 |  |
| HEG1 | 1,86E-232 | 1,17 |  | | MAPRE1 | | 4,30E-62 | -1,04 |  |
| ARL4C | 3,46E-232 | 1,21 |  | | LLGL2 | | 2,71E-61 | -1,07 |  |
| CD14 | 5,31E-231 | 1,19 |  | | NOX1 | | 2,82E-61 | -1,14 |  |
| PTGIS | 9,87E-231 | 1,40 |  | | CLDN3 | | 4,49E-61 | -1,08 |  |
| CLDN11 | 1,13E-230 | 1,48 |  | | SLC19A1 | | 5,42E-61 | -1,08 |  |
| RFTN1 | 2,41E-230 | 1,15 |  | | SIGMAR1 | | 1,04E-60 | -1,05 |  |
| GPR68 | 4,21E-230 | 1,19 |  | | ENTPD6 | | 1,20E-60 | -1,07 |  |
| CALHM2 | 1,33E-227 | 1,16 |  | | OCLN | | 1,21E-60 | -1,08 |  |
| JAM3 | 5,16E-227 | 1,20 |  | | NME1 | | 3,01E-60 | -1,05 |  |
| NOTCH3 | 5,89E-227 | 1,17 |  | | POLE | | 5,42E-60 | -1,07 |  |
| TGFB3 | 1,80E-224 | 1,24 |  | | LRRC8D | | 5,92E-60 | -1,04 |  |
| NOX4 | 3,78E-224 | 1,40 |  | | TMPRSS2 | | 9,25E-60 | -1,07 |  |
| GUCY1B3 | 4,30E-224 | 1,24 |  | | VDAC1 | | 3,49E-59 | -1,04 |  |
| AXL | 1,91E-223 | 1,17 |  | | ACTL6A | | 4,20E-59 | -1,06 |  |
| TYROBP | 2,43E-223 | 1,21 |  | | SH3YL1 | | 4,20E-59 | -1,06 |  |
| RASSF2 | 1,97E-221 | 1,24 |  | | SLC2A8 | | 8,10E-59 | -1,07 |  |
| RHOJ | 2,18E-220 | 1,26 |  | | SLC7A1 | | 1,11E-58 | -1,07 |  |
| CXCL12 | 1,86E-219 | 1,33 |  | | ETS2 | | 1,61E-58 | -1,06 |  |
| CERCAM | 5,44E-219 | 1,19 |  | | SLC27A2 | | 1,96E-58 | -1,12 |  |
| MCC | 1,20E-218 | 1,32 |  | | SYAP1 | | 2,80E-58 | -1,05 |  |
| C3AR1 | 3,71E-216 | 1,21 |  | | MEP1A | | 4,01E-58 | -1,18 |  |
| PPAPDC1A | 5,45E-216 | 1,41 |  | | CD320 | | 5,99E-58 | -1,06 |  |
| ENTPD1 | 3,09E-215 | 1,14 |  | | ITGA6 | | 7,74E-58 | -1,06 |  |
| CD93 | 3,27E-212 | 1,14 |  | | RAB17 | | 1,14E-57 | -1,07 |  |
| LAPTM5 | 3,72E-212 | 1,15 |  | | MLEC | | 2,20E-57 | -1,05 |  |
| GNG11 | 4,90E-212 | 1,20 |  | | KNTC1 | | 4,86E-57 | -1,07 |  |
| HMCN1 | 7,33E-212 | 1,36 |  | | USH1C | | 6,14E-57 | -1,07 |  |
| STOM | 8,38E-210 | 1,15 |  | | GPD1L | | 1,37E-56 | -1,06 |  |
| S1PR3 | 4,77E-209 | 1,21 |  | | TLCD1 | | 2,05E-56 | -1,09 |  |
| CAV1 | 1,02E-208 | 1,18 |  | | IQGAP3 | | 8,14E-56 | -1,08 |  |
| GEM | 2,28E-208 | 1,20 |  | | NAE1 | | 1,25E-55 | -1,05 |  |
| SDC2 | 3,35E-208 | 1,18 |  | | KIF18A | | 3,16E-55 | -1,13 |  |
| JAM2 | 4,64E-208 | 1,27 |  | | RFFL | | 5,72E-55 | -1,07 |  |
| SYNC | 8,09E-208 | 1,33 |  | | TJP2 | | 9,14E-55 | -1,05 |  |
| CSF1R | 1,30E-207 | 1,18 |  | | GLO1 | | 1,77E-54 | -1,05 |  |
| MRC1 | 2,66E-206 | 1,30 |  | | PPP1R16A | | 2,29E-54 | -1,07 |  |
| MYH10 | 3,09E-205 | 1,20 |  | | TJP3 | | 3,20E-54 | -1,07 |  |
| MSR1 | 1,50E-204 | 1,33 |  | | FGFR3 | | 6,41E-54 | -1,13 |  |
| TIE1 | 2,72E-204 | 1,22 |  | | ROCK2 | | 1,63E-53 | -1,06 |  |
| CAV2 | 1,23E-203 | 1,22 |  | | TRIB3 | | 1,70E-53 | -1,09 |  |
| SLC2A3 | 5,67E-202 | 1,27 |  | | DNAJC9 | | 1,79E-53 | -1,07 |  |
| RECK | 3,44E-201 | 1,28 |  | | MYO1A | | 2,04E-53 | -1,10 |  |
| C3 | 1,28E-200 | 1,26 |  | | HSD17B10 | | 2,54E-53 | -1,05 |  |
| PKD2 | 1,05E-199 | 1,18 |  | | TFRC | | 3,46E-53 | -1,07 |  |
| **PDGFRB** | **1,10E-199** | **1,15** |  | | SCARB1 | | 3,89E-53 | -1,06 |  |
| CLEC2B | 1,19E-198 | 1,23 |  | | CAPN10 | | 6,22E-53 | -1,14 |  |
| ADGRA2 | 1,32E-198 | 1,16 |  | | PVR | | 7,45E-53 | -1,06 |  |
| CLIC4 | 1,49E-198 | 1,13 |  | | FARP1 | | 1,12E-52 | -1,08 |  |
| ATP2B4 | 1,80E-197 | 1,16 |  | | HN1L | | 2,65E-52 | -1,04 |  |
| SYT11 | 8,69E-197 | 1,18 |  | | KIF20A | | 3,75E-52 | -1,09 |  |
| PTPRM | 1,33E-196 | 1,15 |  | | ANKRD27 | | 5,28E-52 | -1,05 |  |
| VCAM1 | 2,78E-196 | 1,24 |  | | SLC26A6 | | 6,00E-52 | -1,06 |  |
| APOE | 9,66E-194 | 1,23 |  | | STAMBP | | 1,02E-51 | -1,04 |  |
| MFGE8 | 4,61E-193 | 1,13 |  | | STK26 | | 1,44E-51 | -1,08 |  |
| ANK2 | 1,33E-192 | 1,41 |  | | SVIP | | 3,19E-51 | -1,08 |  |
| FZD1 | 1,51E-192 | 1,18 |  | | SLC11A2 | | 4,86E-51 | -1,07 |  |
| FYN | 1,52E-191 | 1,19 |  | | NLN | | 8,13E-51 | -1,07 |  |
| LAIR1 | 1,69E-191 | 1,18 |  | | FAM84B | | 1,69E-50 | -1,07 |  |
| AHNAK2 | 4,73E-191 | 1,29 |  | | F2RL1 | | 3,44E-50 | -1,07 |  |
| PLXND1 | 6,56E-191 | 1,13 |  | | ZMYND19 | | 5,16E-50 | -1,05 |  |
| GUCY1A3 | 1,64E-190 | 1,22 |  | | ST14 | | 9,49E-50 | -1,06 |  |
| LDB2 | 3,96E-190 | 1,20 |  | | KIF14 | | 1,06E-49 | -1,11 |  |
| HTR2B | 7,99E-190 | 1,53 |  | | SLCO4A1 | | 1,21E-49 | -1,09 |  |
| DLC1 | 1,45E-189 | 1,17 |  | | CPNE1 | | 2,62E-49 | -1,06 |  |
| ITPRIP | 3,63E-189 | 1,13 |  | | ACSL6 | | 3,60E-49 | -1,27 |  |
| VASN | 4,25E-189 | 1,13 |  | | ACVR1B | | 7,95E-49 | -1,06 |  |
| HCLS1 | 5,12E-189 | 1,20 |  | | GPA33 | | 8,82E-49 | -1,09 |  |
| KCTD12 | 5,89E-189 | 1,22 |  | | SLC25A13 | | 9,70E-49 | -1,06 |  |
| EMILIN1 | 1,17E-188 | 1,18 |  | | DSG2 | | 1,49E-48 | -1,06 |  |
| IGF1 | 2,03E-188 | 1,41 |  | | CASP8 | | 4,59E-48 | -1,06 |  |
| THBD | 7,17E-188 | 1,20 |  | | CRCP | | 1,02E-47 | -1,05 |  |
| FCER1G | 1,15E-187 | 1,22 |  | | SLC29A2 | | 1,55E-47 | -1,06 |  |
| KCNMA1 | 2,26E-187 | 1,32 |  | | INADL | | 2,34E-47 | -1,07 |  |
| TRPC1 | 2,76E-187 | 1,30 |  | | KCNE3 | | 2,37E-47 | -1,06 |  |
| GJA1 | 2,26E-186 | 1,17 |  | | SLC3A1 | | 3,61E-47 | -1,17 |  |
| DAPK1 | 5,34E-186 | 1,22 |  | | FNBP1L | | 7,33E-47 | -1,05 |  |
| CD163 | 4,75E-185 | 1,29 |  | | ECT2 | | 1,49E-46 | -1,08 |  |
| SLC1A3 | 8,08E-185 | 1,23 |  | | CLCN3 | | 2,35E-46 | -1,05 |  |
| CCDC8 | 6,54E-183 | 1,20 |  | | CC2D1A | | 3,63E-46 | -1,05 |  |
| TGFB1 | 3,59E-182 | 1,19 |  | | EREG | | 4,19E-46 | -1,20 |  |
| TNFSF13B | 9,36E-182 | 1,24 |  | | ASPM | | 4,53E-46 | -1,10 |  |
| PDLIM4 | 1,56E-181 | 1,21 |  | | CLN3 | | 1,05E-45 | -1,05 |  |
| SYNPO | 1,81E-181 | 1,13 |  | | CKAP5 | | 1,06E-45 | -1,04 |  |
| SLC16A2 | 1,81E-181 | 1,21 |  | | NUP35 | | 1,93E-45 | -1,05 |  |
| STEAP4 | 2,01E-181 | 1,34 |  | | JPH1 | | 2,16E-45 | -1,09 |  |
| UCHL1 | 2,18E-181 | 1,30 |  | | MCTS1 | | 2,65E-45 | -1,04 |  |
| C5AR1 | 2,73E-181 | 1,26 |  | | SLC12A2 | | 2,89E-45 | -1,07 |  |
| PIK3CD | 2,98E-181 | 1,15 |  | | CLNS1A | | 3,03E-45 | -1,05 |  |
| ENG | 5,46E-181 | 1,11 |  | | EVPL | | 3,10E-45 | -1,07 |  |
| FAM26E | 7,52E-181 | 1,18 |  | | KIAA1524 | | 3,55E-45 | -1,10 |  |
| ROR2 | 3,45E-180 | 1,28 |  | | MRPL42 | | 3,78E-45 | -1,05 |  |
| TNS2 | 5,60E-180 | 1,12 |  | | MET | | 4,65E-45 | -1,07 |  |
| ACTN1 | 1,48E-179 | 1,09 |  | | SLC7A5 | | 5,60E-45 | -1,07 |  |
| NCF2 | 8,24E-179 | 1,27 |  | | TSPAN12 | | 1,08E-44 | -1,09 |  |
| PSAP | 2,26E-178 | 1,06 |  | | DGAT1 | | 1,18E-44 | -1,06 |  |
| KLF9 | 8,29E-178 | 1,25 |  | | PRR7 | | 1,58E-44 | -1,08 |  |
| HCK | 2,43E-177 | 1,20 |  | | SHANK2 | | 1,94E-44 | -1,08 |  |
| CEP170 | 2,73E-177 | 1,14 |  | | SLC25A5 | | 2,25E-44 | -1,04 |  |
| C7 | 3,63E-177 | 1,45 |  | | PPAP2C | | 4,60E-44 | -1,07 |  |
| GNG2 | 4,39E-177 | 1,21 |  | | ADD3 | | 5,20E-44 | -1,04 |  |
| LY86 | 7,99E-177 | 1,18 |  | | ATP9A | | 6,52E-44 | -1,06 |  |
| PIP4K2A | 2,30E-176 | 1,09 |  | | CEACAM1 | | 7,25E-44 | -1,08 |  |
| TMEM119 | 1,99E-175 | 1,21 |  | | DPEP1 | | 7,44E-44 | -1,13 |  |
| SERPINE1 | 3,91E-173 | 1,24 |  | | RCCD1 | | 9,08E-44 | -1,04 |  |
| OSMR | 4,38E-173 | 1,23 |  | | MMP15 | | 1,12E-43 | -1,05 |  |
| TMEM47 | 8,18E-173 | 1,25 |  | | FAM120A | | 1,27E-43 | -1,04 |  |
| LZTS1 | 6,95E-172 | 1,16 |  | | CD46 | | 2,03E-43 | -1,04 |  |
| CD37 | 1,29E-171 | 1,24 |  | | PKP2 | | 2,09E-43 | -1,08 |  |
| FHL1 | 1,39E-171 | 1,23 |  | | XK | | 2,80E-43 | -1,10 |  |
| TLR1 | 1,50E-171 | 1,29 |  | | RALGAPA2 | | 7,83E-43 | -1,07 |  |
| DCLK1 | 1,80E-171 | 1,42 |  | | UGT8 | | 1,16E-42 | -1,08 |  |
| ITGB2 | 1,81E-171 | 1,20 |  | | RAB15 | | 2,15E-42 | -1,05 |  |
| SGCD | 1,82E-171 | 1,23 |  | | P2RY2 | | 3,55E-42 | -1,08 |  |
| IL10RA | 2,93E-171 | 1,21 |  | | ILDR1 | | 5,39E-42 | -1,07 |  |
| ADGRL4 | 9,33E-171 | 1,22 |  | | IYD | | 7,51E-42 | -1,13 |  |
| FCGR2A | 9,40E-171 | 1,21 |  | | MLLT4 | | 1,11E-41 | -1,04 |  |
| TRPV2 | 2,21E-170 | 1,14 |  | | MGST2 | | 2,83E-41 | -1,04 |  |
| DBN1 | 2,25E-170 | 1,15 |  | | USP6NL | | 3,11E-41 | -1,06 |  |
| ANXA1 | 3,38E-170 | 1,19 |  | | STOML2 | | 3,75E-41 | -1,04 |  |
| NLGN4X | 4,06E-170 | 1,30 |  | | ATP1A1 | | 5,42E-41 | -1,04 |  |
| GNAI2 | 2,16E-169 | 1,09 |  | | CENPJ | | 9,15E-41 | -1,11 |  |
| MRGPRF | 5,58E-169 | 1,25 |  | | STAP2 | | 3,47E-40 | -1,05 |  |
| CXCR4 | 5,69E-169 | 1,20 |  | | EPS8L2 | | 4,83E-40 | -1,04 |  |
| MMP14 | 1,21E-167 | 1,10 |  | | RAB22A | | 6,18E-40 | -1,05 |  |
| SNX18 | 6,80E-167 | 1,11 |  | | BCAP31 | | 7,21E-40 | -1,03 |  |
| MYLK | 8,19E-167 | 1,19 |  | | LRRC1 | | 9,05E-40 | -1,05 |  |
| LSP1 | 2,00E-166 | 1,14 |  | | NRAS | | 1,29E-39 | -1,05 |  |
| HSPG2 | 3,10E-166 | 1,10 |  | | DLG3 | | 3,01E-39 | -1,04 |  |
| PIK3R5 | 5,06E-166 | 1,18 |  | | LRP8 | | 4,56E-39 | -1,10 |  |
| RAMP1 | 1,57E-165 | 1,25 |  | | CLDN7 | | 4,69E-39 | -1,05 |  |
| NOTCH2 | 6,61E-165 | 1,08 |  | | MUC13 | | 5,14E-39 | -1,07 |  |
| OLR1 | 7,36E-165 | 1,42 |  | | ARRDC1 | | 5,93E-39 | -1,04 |  |
| NCKAP1L | 1,23E-164 | 1,15 |  | | CRB3 | | 6,74E-39 | -1,04 |  |
| CNTNAP1 | 4,09E-164 | 1,17 |  | | LYPD6 | | 7,81E-39 | -1,09 |  |
| SLC11A1 | 6,24E-164 | 1,22 |  | | PLCH1 | | 9,00E-39 | -1,07 |  |
| ITGBL1 | 9,18E-164 | 1,39 |  | | EFNA2 | | 9,20E-39 | -1,11 |  |
| LRRC32 | 1,67E-163 | 1,12 |  | | TAB3 | | 9,76E-39 | -1,08 |  |
| KIRREL | 3,26E-163 | 1,17 |  | | DCXR | | 2,78E-38 | -1,05 |  |
| KCNMB1 | 4,41E-163 | 1,29 |  | | ATP7B | | 3,37E-38 | -1,08 |  |
| PARVG | 7,84E-163 | 1,13 |  | | SHROOM4 | | 3,41E-38 | -1,06 |  |
| SLC22A17 | 4,65E-162 | 1,14 |  | | RAB40C | | 1,22E-37 | -1,04 |  |
| ADGRF5 | 5,52E-162 | 1,18 |  | | PPP1CC | | 1,34E-37 | -1,03 |  |
| ROR1 | 7,30E-162 | 1,25 |  | | ATP13A3 | | 1,43E-37 | -1,06 |  |
| DOK3 | 1,28E-161 | 1,19 |  | | DPP3 | | 1,93E-37 | -1,04 |  |
| SLC24A3 | 1,37E-161 | 1,22 |  | | CNKSR1 | | 2,49E-37 | -1,06 |  |
| ECSCR | 4,47E-161 | 1,15 |  | | ACOX1 | | 5,08E-37 | -1,05 |  |
| STAB1 | 1,71E-160 | 1,14 |  | | AMACR | | 6,06E-37 | -1,08 |  |
| APBB1IP | 1,95E-160 | 1,21 |  | | SUCLG2 | | 7,94E-37 | -1,04 |  |
| HAVCR2 | 2,37E-160 | 1,21 |  | | SYT7 | | 9,10E-37 | -1,07 |  |
| CLEC7A | 6,39E-160 | 1,25 |  | | SLC13A3 | | 1,36E-36 | -1,16 |  |
| S1PR1 | 1,03E-159 | 1,17 |  | | RIF1 | | 2,06E-36 | -1,06 |  |
| RNF144A | 1,35E-159 | 1,17 |  | | OCRL | | 2,62E-36 | -1,04 |  |
| STAT2 | 2,35E-159 | 1,10 |  | | HINT1 | | 3,33E-36 | -1,03 |  |
| ICAM1 | 9,70E-159 | 1,11 |  | | BAIAP2L1 | | 5,45E-36 | -1,05 |  |
| TMEM204 | 1,87E-158 | 1,14 |  | | LRFN4 | | 7,45E-36 | -1,05 |  |
| LRCH2 | 2,63E-158 | 1,32 |  | | XRCC6BP1 | | 8,60E-36 | -1,07 |  |
| NID2 | 1,35E-157 | 1,18 |  | | XKRX | | 9,17E-36 | -1,11 |  |
| FN1 | 1,48E-157 | 1,17 |  | | SCRIB | | 1,02E-35 | -1,04 |  |
| ITGA7 | 2,58E-157 | 1,18 |  | | SLC7A6 | | 1,32E-35 | -1,05 |  |
| KCNJ8 | 2,81E-157 | 1,21 |  | | SLC26A3 | | 1,56E-35 | -1,22 |  |
| MMP16 | 5,90E-157 | 1,25 |  | | CDHR2 | | 1,84E-35 | -1,10 |  |
| AHNAK | 9,23E-157 | 1,11 |  | | CEACAM5 | | 1,94E-35 | -1,05 |  |
| CD86 | 2,42E-156 | 1,21 |  | | ITPR3 | | 2,27E-35 | -1,05 |  |
| PDPN | 3,01E-156 | 1,19 |  | | KIF21A | | 3,58E-35 | -1,06 |  |
| ITGAV | 3,59E-156 | 1,09 |  | | NUBPL | | 3,84E-35 | -1,06 |  |
| CD84 | 4,13E-156 | 1,23 |  | | SMPD3 | | 4,51E-35 | -1,06 |  |
| RGS4 | 1,02E-155 | 1,30 |  | | LRP11 | | 6,70E-35 | -1,04 |  |
| PLXNC1 | 1,50E-155 | 1,26 |  | | CAPRIN1 | | 1,63E-34 | -1,03 |  |
| JPH2 | 2,96E-155 | 1,21 |  | | WNK2 | | 1,66E-34 | -1,05 |  |
| CCR1 | 3,71E-155 | 1,19 |  | | SLC6A4 | | 1,99E-34 | -1,17 |  |
| PLA2G5 | 1,01E-154 | 1,32 |  | | MPP7 | | 2,11E-34 | -1,09 |  |
| CD53 | 1,24E-154 | 1,17 |  | | MTFR1 | | 2,15E-34 | -1,05 |  |
| RGMA | 1,43E-154 | 1,20 |  | | GRB7 | | 2,25E-34 | -1,06 |  |
| LAMA2 | 2,60E-154 | 1,22 |  | | HSP90AB1 | | 2,93E-34 | -1,02 |  |
| SFRP1 | 7,12E-154 | 1,46 |  | | LRRC8B | | 3,10E-34 | -1,06 |  |
| ITGA11 | 1,14E-153 | 1,16 |  | | IL20RA | | 5,82E-34 | -1,10 |  |
| FZD4 | 1,87E-153 | 1,11 |  | | VIPR1 | | 6,89E-34 | -1,08 |  |
| CNTN4 | 4,36E-153 | 1,28 |  | | ADAP1 | | 7,73E-34 | -1,04 |  |
| PHLDA3 | 8,66E-153 | 1,15 |  | | MASTL | | 1,35E-33 | -1,08 |  |
| ADAP2 | 1,50E-152 | 1,13 |  | | PARM1 | | 2,83E-33 | -1,07 |  |
| PLXDC1 | 5,44E-150 | 1,16 |  | | LRRC16A | | 3,84E-33 | -1,05 |  |
| PTAFR | 1,34E-149 | 1,15 |  | | EFNA4 | | 6,42E-33 | -1,06 |  |
| PDE2A | 3,37E-149 | 1,20 |  | | WARS2 | | 6,46E-33 | -1,06 |  |
| GJC1 | 3,47E-149 | 1,23 |  | | SLC19A3 | | 1,34E-32 | -1,14 |  |
| AIF1 | 6,73E-149 | 1,16 |  | | G3BP1 | | 1,45E-32 | -1,03 |  |
| EVI2B | 4,27E-148 | 1,23 |  | | NUDT1 | | 1,82E-32 | -1,05 |  |
| FPR3 | 1,16E-147 | 1,21 |  | | SLC30A2 | | 3,08E-32 | -1,13 |  |
| FCN1 | 2,61E-147 | 1,27 |  | | EXOC6 | | 6,46E-32 | -1,05 |  |
| CSF2RB | 3,75E-147 | 1,23 |  | | RABGGTB | | 1,07E-31 | -1,04 |  |
| TNFSF4 | 4,76E-147 | 1,25 |  | | SLC3A2 | | 1,10E-31 | -1,03 |  |
| ENOX1 | 1,50E-146 | 1,18 |  | | HDAC11 | | 1,59E-31 | -1,05 |  |
| VCL | 2,75E-146 | 1,07 |  | | TMEM63A | | 1,78E-31 | -1,05 |  |
| TEK | 2,98E-146 | 1,17 |  | | STXBP6 | | 2,05E-31 | -1,07 |  |
| CYTH4 | 3,25E-146 | 1,14 |  | | GRM8 | | 2,35E-31 | -1,18 |  |
| CD34 | 6,58E-146 | 1,10 |  | | TDP1 | | 2,49E-31 | -1,05 |  |
| NPR3 | 1,05E-144 | 1,35 |  | | SLC39A11 | | 3,20E-31 | -1,04 |  |
| UNC5B | 2,17E-144 | 1,12 |  | | CCT3 | | 4,27E-31 | -1,03 |  |
| MYADM | 3,77E-144 | 1,11 |  | | MRPL44 | | 6,66E-31 | -1,04 |  |
| SIGLEC1 | 3,99E-144 | 1,21 |  | | PDZD3 | | 9,13E-31 | -1,08 |  |
| CLEC14A | 7,15E-144 | 1,11 |  | | TTC17 | | 1,05E-30 | -1,04 |  |
| ITGAX | 7,92E-144 | 1,16 |  | | PTPRO | | 1,22E-30 | -1,13 |  |
| DEGS1 | 1,13E-143 | 1,12 |  | | SEMA4G | | 1,34E-30 | -1,05 |  |
| PPP3CB | 3,48E-143 | 1,08 |  | | HIP1R | | 1,59E-30 | -1,03 |  |
| ITPR1 | 9,45E-143 | 1,17 |  | | CDHR5 | | 3,93E-30 | -1,06 |  |
| SLA | 1,85E-142 | 1,16 |  | | FMR1 | | 4,90E-30 | -1,04 |  |
| PALM | 2,13E-142 | 1,15 |  | | AGPAT2 | | 7,43E-30 | -1,03 |  |
| SLC9A9 | 1,13E-141 | 1,17 |  | | TMEM123 | | 8,68E-30 | -1,03 |  |
| FPR1 | 1,15E-141 | 1,22 |  | | SLC16A1 | | 1,01E-29 | -1,07 |  |
| SNCA | 9,87E-141 | 1,23 |  | | CYP4F12 | | 1,09E-29 | -1,06 |  |
| PARD6G | 5,71E-140 | 1,12 |  | | ITCH | | 1,24E-29 | -1,07 |  |
| THBS1 | 7,16E-140 | 1,16 |  | | ENOX2 | | 1,45E-29 | -1,05 |  |
| GPR183 | 2,65E-139 | 1,22 |  | | LIMD1 | | 1,48E-29 | -1,04 |  |
| TTC7B | 3,15E-139 | 1,17 |  | | CNNM4 | | 1,61E-29 | -1,05 |  |
| ALDH1A3 | 3,28E-139 | 1,16 |  | | SPATA13 | | 2,12E-29 | -1,05 |  |
| TUSC3 | 4,97E-139 | 1,20 |  | | SLC44A3 | | 3,63E-29 | -1,06 |  |
| BACE1 | 2,58E-138 | 1,11 |  | | MYO1D | | 6,90E-29 | -1,05 |  |
| LRRC8C | 2,59E-138 | 1,14 |  | | CEP295 | | 7,51E-29 | -1,05 |  |
| TUB | 4,18E-138 | 1,21 |  | | EPHB3 | | 7,58E-29 | -1,06 |  |
| GNB5 | 1,06E-137 | 1,12 |  | | STRN | | 1,34E-28 | -1,05 |  |
| BTK | 3,30E-137 | 1,17 |  | | GNG4 | | 1,49E-28 | -1,15 |  |
| GLRB | 9,62E-137 | 1,25 |  | | C1QBP | | 1,79E-28 | -1,03 |  |
| CDH5 | 1,80E-136 | 1,17 |  | | LRP4 | | 3,75E-28 | -1,12 |  |
| TREM1 | 1,88E-136 | 1,29 |  | | ENPP3 | | 4,97E-28 | -1,15 |  |
| PARVA | 2,12E-136 | 1,09 |  | | LGALS4 | | 7,23E-28 | -1,05 |  |
| ANTXR2 | 1,05E-135 | 1,11 |  | | NKD1 | | 1,32E-27 | -1,10 |  |
| ABCA1 | 2,97E-135 | 1,16 |  | | MYO10 | | 2,47E-27 | -1,04 |  |
| CSF1 | 1,32E-134 | 1,10 |  | | DUOX2 | | 2,53E-27 | -1,18 |  |
| SELPLG | 3,29E-134 | 1,14 |  | | CGN | | 2,57E-27 | -1,05 |  |
| RHOQ | 4,68E-134 | 1,10 |  | | PRKAR1B | | 9,23E-27 | -1,04 |  |
| LPAR1 | 4,72E-134 | 1,17 |  | | TMUB1 | | 9,40E-27 | -1,04 |  |
| ANXA5 | 4,93E-134 | 1,07 |  | | LRBA | | 1,05E-26 | -1,04 |  |
| HLA-DMB | 8,43E-134 | 1,15 |  | | CAMK2N1 | | 4,50E-26 | -1,04 |  |
| TIAM1 | 6,04E-133 | 1,22 |  | | CCRL2 | | 7,82E-26 | -1,05 |  |
| CLDN5 | 6,05E-133 | 1,14 |  | | SLC26A2 | | 9,24E-26 | -1,09 |  |
| LILRB2 | 6,56E-133 | 1,23 |  | | ATP8B1 | | 9,51E-26 | -1,05 |  |
| SGIP1 | 6,79E-133 | 1,20 |  | | RAB25 | | 9,61E-26 | -1,05 |  |
| LCP2 | 3,04E-132 | 1,20 |  | | EPS8 | | 1,06E-25 | -1,03 |  |
| CPEB1 | 3,20E-132 | 1,20 |  | | TMEM5 | | 1,13E-25 | -1,05 |  |
| RASGRP2 | 5,28E-132 | 1,17 |  | | SLC16A9 | | 1,63E-25 | -1,11 |  |
| CD300A | 1,28E-131 | 1,16 |  | | MEN1 | | 2,22E-25 | -1,03 |  |
| SYNGR1 | 4,78E-131 | 1,16 |  | | GPC4 | | 4,85E-25 | -1,06 |  |
| APLNR | 9,06E-131 | 1,13 |  | | MAGI3 | | 9,19E-25 | -1,04 |  |
| PLCG2 | 1,76E-130 | 1,15 |  | | C2CD4A | | 2,77E-24 | -1,13 |  |
| DOCK8 | 3,33E-130 | 1,22 |  | | LIN7C | | 2,97E-24 | -1,03 |  |
| MCAM | 6,11E-130 | 1,13 |  | | FASN | | 3,00E-24 | -1,05 |  |
| GLIPR1 | 6,71E-130 | 1,17 |  | | CARD14 | | 3,55E-24 | -1,06 |  |
| IL6ST | 9,40E-130 | 1,10 |  | | ADORA2B | | 5,83E-24 | -1,07 |  |
| GSN | 1,95E-129 | 1,11 |  | | PLP2 | | 6,22E-24 | -1,03 |  |
| Mar/01 | 4,41E-129 | 1,21 |  | | FXYD3 | | 6,30E-24 | -1,06 |  |
| C10ORF54 | 4,77E-129 | 1,10 |  | | MST1R | | 8,50E-24 | -1,05 |  |
| POPDC2 | 1,58E-128 | 1,25 |  | | COG3 | | 8,56E-24 | -1,04 |  |
| LPPR4 | 2,62E-128 | 1,23 |  | | LGR5 | | 1,03E-23 | -1,12 |  |
| FOLR2 | 5,34E-128 | 1,16 |  | | PTK6 | | 1,20E-23 | -1,04 |  |
| ABCA8 | 6,85E-128 | 1,44 |  | | ABHD12 | | 1,30E-23 | -1,04 |  |
| MARCO | 2,87E-127 | 1,30 |  | | IRAK1 | | 1,39E-23 | -1,03 |  |
| VAMP5 | 3,71E-127 | 1,12 |  | | ADGRA3 | | 1,58E-23 | -1,04 |  |
| PCDH9 | 7,44E-127 | 1,24 |  | | GPRC5C | | 2,20E-23 | -1,03 |  |
| HCST | 1,19E-126 | 1,13 |  | | ITGA2 | | 2,77E-23 | -1,05 |  |
| HS3ST3B1 | 1,30E-126 | 1,26 |  | | DLG1 | | 3,41E-23 | -1,04 |  |
| RAB8B | 1,45E-126 | 1,11 |  | | AZGP1 | | 4,70E-23 | -1,09 |  |
| LYVE1 | 2,88E-126 | 1,31 |  | | TMEM30B | | 5,76E-23 | -1,05 |  |
| ACKR1 | 1,01E-125 | 1,24 |  | | ZGPAT | | 6,42E-23 | -1,03 |  |
| PEAR1 | 1,32E-125 | 1,15 |  | | NIPSNAP1 | | 6,98E-23 | -1,03 |  |
| CD74 | 2,49E-125 | 1,10 |  | | PARD6A | | 9,34E-23 | -1,03 |  |
| LIMS2 | 5,78E-125 | 1,12 |  | | CDCA4 | | 1,02E-22 | -1,04 |  |
| LILRB1 | 7,83E-125 | 1,15 |  | | CLDN12 | | 1,31E-22 | -1,04 |  |
| EPHA3 | 1,44E-124 | 1,29 |  | | ACVR2B | | 1,55E-22 | -1,04 |  |
| AMPH | 1,99E-124 | 1,18 |  | | ETNK1 | | 1,59E-22 | -1,04 |  |
| RASGRP3 | 6,65E-124 | 1,13 |  | | SNX5 | | 1,60E-22 | -1,04 |  |
| PTPRS | 1,62E-123 | 1,15 |  | | MFSD3 | | 2,04E-22 | -1,05 |  |
| GAP43 | 6,45E-123 | 1,34 |  | | SLC25A3 | | 2,16E-22 | -1,02 |  |
| CHRNA3 | 8,15E-123 | 1,25 |  | | DFFA | | 2,66E-22 | -1,04 |  |
| NEGR1 | 8,96E-123 | 1,25 |  | | NAPEPLD | | 3,30E-22 | -1,05 |  |
| TLR2 | 1,10E-122 | 1,16 |  | | CNPY2 | | 3,70E-22 | -1,03 |  |
| TFPI | 4,07E-122 | 1,18 |  | | LRRC45 | | 4,77E-22 | -1,03 |  |
| ARHGAP10 | 8,16E-122 | 1,14 |  | | GPI | | 5,40E-22 | -1,02 |  |
| BVES | 2,47E-121 | 1,21 |  | | PTCH1 | | 6,19E-22 | -1,05 |  |
| FYB | 2,79E-121 | 1,21 |  | | NOSTRIN | | 6,29E-22 | -1,06 |  |
| NLGN2 | 3,56E-121 | 1,10 |  | | IDE | | 1,20E-21 | -1,03 |  |
| ADCY2 | 1,53E-120 | 1,16 |  | | CLDN1 | | 1,87E-21 | -1,07 |  |
| CAP2 | 2,56E-120 | 1,20 |  | | PTPRD | | 1,98E-21 | -1,10 |  |
| GJA4 | 3,25E-120 | 1,13 |  | | PNN | | 2,03E-21 | -1,03 |  |
| LAT2 | 3,64E-120 | 1,13 |  | | MGST1 | | 2,37E-21 | -1,05 |  |
| RGS16 | 4,68E-120 | 1,14 |  | | ADGRG1 | | 2,46E-21 | -1,03 |  |
| ENO2 | 6,70E-120 | 1,17 |  | | GPHN | | 2,63E-21 | -1,05 |  |
| LRRK2 | 1,06E-119 | 1,26 |  | | JUP | | 2,87E-21 | -1,03 |  |
| NALCN | 2,19E-119 | 1,31 |  | | FANCG | | 3,41E-21 | -1,03 |  |
| DNAJB4 | 2,66E-119 | 1,16 |  | | PDS5A | | 3,41E-21 | -1,04 |  |
| GPR65 | 4,91E-119 | 1,20 |  | | CEACAM7 | | 3,67E-21 | -1,15 |  |
| HLA-DPB1 | 1,11E-118 | 1,17 |  | | SLC39A8 | | 4,30E-21 | -1,06 |  |
| SLC12A4 | 1,42E-118 | 1,08 |  | | C4BPA | | 7,46E-21 | -1,10 |  |
| PTGER3 | 1,99E-118 | 1,21 |  | | FRK | | 9,58E-21 | -1,07 |  |
| SVIL | 2,93E-118 | 1,10 |  | | SRI | | 1,29E-20 | -1,03 |  |
| ANOS1 | 5,31E-118 | 1,22 |  | | PTPRK | | 1,39E-20 | -1,03 |  |
| AGTR1 | 1,32E-117 | 1,41 |  | | SEMA5A | | 1,46E-20 | -1,05 |  |
| TLR5 | 2,72E-117 | 1,15 |  | | PDCD6IP | | 1,56E-20 | -1,03 |  |
| CD36 | 2,78E-117 | 1,34 |  | | CHP2 | | 2,02E-20 | -1,11 |  |
| HTR2A | 4,26E-117 | 1,23 |  | | IGSF8 | | 2,35E-20 | -1,04 |  |
| FLNC | 4,89E-117 | 1,16 |  | | MUC20 | | 3,27E-20 | -1,06 |  |
| SGK1 | 7,03E-117 | 1,11 |  | | STEAP3 | | 3,68E-20 | -1,04 |  |
| EPB41L3 | 8,53E-117 | 1,26 |  | | MMP24 | | 4,27E-20 | -1,04 |  |
| FNBP1 | 1,12E-116 | 1,11 |  | | TSPAN33 | | 5,22E-20 | -1,04 |  |
| TREM2 | 1,57E-116 | 1,17 |  | | FUT1 | | 7,88E-20 | -1,05 |  |
| SLC16A7 | 3,72E-116 | 1,27 |  | | SSFA2 | | 8,86E-20 | -1,04 |  |
| GPR84 | 6,76E-116 | 1,20 |  | | SPINT1 | | 1,25E-19 | -1,03 |  |
| FAM126A | 7,03E-116 | 1,19 |  | | EEA1 | | 1,60E-19 | -1,05 |  |
| LCP1 | 4,23E-115 | 1,13 |  | | DSP | | 1,74E-19 | -1,03 |  |
| HLA-DOA | 5,04E-115 | 1,16 |  | | PHKA1 | | 1,86E-19 | -1,05 |  |
| DOCK4 | 9,48E-115 | 1,17 |  | | SLC44A4 | | 1,96E-19 | -1,05 |  |
| SUSD2 | 1,15E-114 | 1,19 |  | | RELL2 | | 2,43E-19 | -1,04 |  |
| EMCN | 1,30E-114 | 1,18 |  | | SLC7A11 | | 2,96E-19 | -1,08 |  |
| TNFSF8 | 1,72E-114 | 1,21 |  | | HAS3 | | 3,63E-19 | -1,08 |  |
| PCDHB5 | 2,86E-114 | 1,21 |  | | CHRNA5 | | 3,92E-19 | -1,06 |  |
| LHFPL2 | 2,88E-114 | 1,07 |  | | RALGPS1 | | 3,93E-19 | -1,04 |  |
| HLA-DRA | 3,98E-114 | 1,14 |  | | MTDH | | 8,33E-19 | -1,03 |  |
| GABARAPL1 | 5,66E-114 | 1,11 |  | | ARL4A | | 9,84E-19 | -1,03 |  |
| TTYH2 | 6,28E-114 | 1,14 |  | | SHROOM3 | | 9,96E-19 | -1,04 |  |
| AQP9 | 2,97E-113 | 1,29 |  | | CPNE3 | | 1,01E-18 | -1,03 |  |
| CD300LF | 3,37E-113 | 1,15 |  | | DENND4C | | 1,24E-18 | -1,03 |  |
| HOMER3 | 3,77E-113 | 1,12 |  | | MFSD2A | | 1,44E-18 | -1,05 |  |
| MACF1 | 8,03E-113 | 1,07 |  | | SNX4 | | 1,47E-18 | -1,04 |  |
| Sep/06 | 1,12E-112 | 1,13 |  | | RAB3D | | 2,39E-18 | -1,03 |  |
| AMIGO2 | 1,19E-112 | 1,17 |  | | TWF1 | | 2,65E-18 | -1,03 |  |
| HLA-DPA1 | 4,79E-112 | 1,14 |  | | SERINC3 | | 4,24E-18 | -1,02 |  |
| CD52 | 6,99E-112 | 1,16 |  | | SH3D19 | | 4,90E-18 | -1,03 |  |
| CD69 | 8,47E-112 | 1,31 |  | | GBAS | | 5,11E-18 | -1,03 |  |
| PRKCB | 1,21E-111 | 1,20 |  | | SPRY2 | | 6,63E-18 | -1,04 |  |
| CACNA1C | 2,18E-111 | 1,16 |  | | CD164 | | 7,10E-18 | -1,03 |  |
| HCFC2 | 5,16E-111 | 1,11 |  | | RGMB | | 7,89E-18 | -1,08 |  |
| CYBB | 6,11E-111 | 1,16 |  | | EPHA1 | | 8,50E-18 | -1,04 |  |
| SAMSN1 | 1,90E-110 | 1,22 |  | | RPGRIP1L | | 1,05E-17 | -1,06 |  |
| PDGFRA | 2,34E-110 | 1,13 |  | | IGSF9 | | 1,43E-17 | -1,05 |  |
| HVCN1 | 9,04E-110 | 1,11 |  | | SNTB1 | | 1,56E-17 | -1,05 |  |
| CDH19 | 1,16E-108 | 1,42 |  | | MYO6 | | 1,57E-17 | -1,03 |  |
| PLEK | 1,28E-108 | 1,18 |  | | SLC22A18 | | 1,59E-17 | -1,04 |  |
| MAP3K12 | 1,42E-108 | 1,12 |  | | ATAD3B | | 2,29E-17 | -1,03 |  |
| MYOF | 5,68E-108 | 1,09 |  | | MTM1 | | 2,87E-17 | -1,05 |  |
| PDE4B | 9,19E-108 | 1,17 |  | | ENPP5 | | 4,51E-17 | -1,08 |  |
| TSPAN2 | 2,03E-107 | 1,23 |  | | HEPH | | 4,68E-17 | -1,03 |  |
| CALCRL | 2,31E-107 | 1,16 |  | | PDCD10 | | 4,88E-17 | -1,03 |  |
| PCDH12 | 2,69E-107 | 1,09 |  | | LPAR5 | | 8,33E-17 | -1,04 |  |
| PTPRC | 1,15E-106 | 1,19 |  | | BCL10 | | 1,08E-16 | -1,03 |  |
| HSPA2 | 1,20E-106 | 1,18 |  | | SLC33A1 | | 1,15E-16 | -1,03 |  |
| LRRC4C | 4,51E-106 | 1,19 |  | | EPB41L1 | | 1,58E-16 | -1,04 |  |
| PRKCH | 5,33E-106 | 1,11 |  | | UNC13B | | 2,00E-16 | -1,03 |  |
| TRO | 2,48E-105 | 1,13 |  | | STX17 | | 2,18E-16 | -1,03 |  |
| SDK1 | 2,76E-105 | 1,19 |  | | SDC1 | | 3,28E-16 | -1,03 |  |
| SLC31A2 | 3,75E-105 | 1,12 |  | | LDLRAP1 | | 4,71E-16 | -1,03 |  |
| TGFBR1 | 4,44E-105 | 1,08 |  | | ARHGAP32 | | 7,39E-16 | -1,04 |  |
| RDX | 8,32E-105 | 1,16 |  | | SYPL1 | | 7,44E-16 | -1,02 |  |
| DES | 1,82E-104 | 1,31 |  | | PODXL2 | | 7,80E-16 | -1,04 |  |
| DCHS1 | 2,05E-104 | 1,10 |  | | KRIT1 | | 1,01E-15 | -1,03 |  |
| PPAP2A | 2,20E-104 | 1,10 |  | | CPD | | 1,09E-15 | -1,03 |  |
| TMEM100 | 2,64E-103 | 1,30 |  | | SLC40A1 | | 1,13E-15 | -1,03 |  |
| FGD5 | 2,33E-102 | 1,12 |  | | DGKQ | | 1,61E-15 | -1,03 |  |
| F2R | 2,38E-102 | 1,14 |  | | CASP3 | | 1,92E-15 | -1,03 |  |
| CR1 | 2,90E-102 | 1,28 |  | | KANK1 | | 2,56E-15 | -1,03 |  |
| TLR8 | 3,03E-102 | 1,25 |  | | ABCC2 | | 3,08E-15 | -1,10 |  |
| CD4 | 3,18E-102 | 1,11 |  | | PLEK2 | | 4,85E-15 | -1,03 |  |
| RGS1 | 3,27E-102 | 1,20 |  | | SUMO1 | | 6,06E-15 | -1,02 |  |
| SYNM | 3,69E-102 | 1,30 |  | | ATP11B | | 6,95E-15 | -1,03 |  |
| PCDH17 | 4,73E-102 | 1,17 |  | | LDLRAD3 | | 9,12E-15 | -1,04 |  |
| DPYSL2 | 7,62E-102 | 1,09 |  | | YIPF4 | | 1,06E-14 | -1,04 |  |
| AR | 7,63E-102 | 1,21 |  | | CDC42SE2 | | 1,43E-14 | -1,03 |  |
| FMNL3 | 1,26E-101 | 1,11 |  | | FGFRL1 | | 2,52E-14 | -1,04 |  |
| CD68 | 1,69E-101 | 1,08 |  | | GSDMB | | 2,75E-14 | -1,05 |  |
| GM2A | 1,85E-101 | 1,07 |  | | TNFRSF11A | | 3,07E-14 | -1,06 |  |
| TPBG | 4,65E-101 | 1,15 |  | | ENPP4 | | 3,78E-14 | -1,04 |  |
| SELL | 2,57E-100 | 1,22 |  | | ABCC4 | | 4,16E-14 | -1,05 |  |
| ARHGAP25 | 2,62E-100 | 1,11 |  | | TRPM6 | | 4,99E-14 | -1,11 |  |
| ESR1 | 2,69E-100 | 1,24 |  | | FAM57A | | 6,81E-14 | -1,03 |  |
| SCN7A | 3,92E-100 | 1,43 |  | | PTK2 | | 6,94E-14 | -1,02 |  |
| FZD2 | 4,70E-100 | 1,15 |  | | GSR | | 1,11E-13 | -1,03 |  |
| BMPR1B | 3,24E-99 | 1,30 |  | | ATP5O | | 1,35E-13 | -1,03 |  |
| COL13A1 | 9,95E-99 | 1,18 |  | | CA4 | | 1,78E-13 | -1,13 |  |
| PLAU | 1,44E-98 | 1,12 |  | | SLC5A9 | | 2,73E-13 | -1,07 |  |
| CASQ2 | 1,80E-98 | 1,31 |  | | SLC19A2 | | 2,88E-13 | -1,04 |  |
| ILK | 3,06E-98 | 1,06 |  | | HSD17B8 | | 3,59E-13 | -1,03 |  |
| TPM1 | 6,53E-98 | 1,06 |  | | NF1 | | 3,85E-13 | -1,03 |  |
| STX11 | 1,23E-97 | 1,17 |  | | SPA17 | | 4,00E-13 | -1,04 |  |
| NBEA | 2,43E-97 | 1,27 |  | | ANXA13 | | 4,39E-13 | -1,08 |  |
| SRGAP2 | 2,71E-97 | 1,10 |  | | PRKCI | | 5,03E-13 | -1,03 |  |
| CCR5 | 3,37E-97 | 1,14 |  | | GAL3ST1 | | 8,95E-13 | -1,04 |  |
| CDC42EP3 | 5,15E-97 | 1,12 |  | | EFNA1 | | 1,40E-12 | -1,03 |  |
| CSPG4 | 6,31E-97 | 1,14 |  | | ZDHHC21 | | 1,97E-12 | -1,04 |  |
| ITM2A | 7,95E-97 | 1,17 |  | | CNTNAP2 | | 2,73E-12 | -1,10 |  |
| ATP8B4 | 1,09E-96 | 1,17 |  | | MYO1B | | 3,70E-12 | -1,02 |  |
| ITGB5 | 2,00E-96 | 1,06 |  | | FABP5 | | 4,15E-12 | -1,03 |  |
| ANGPT1 | 4,48E-96 | 1,18 |  | | ERBB2 | | 4,33E-12 | -1,03 |  |
| RRAD | 1,16E-95 | 1,18 |  | | TSPAN8 | | 5,44E-12 | -1,03 |  |
| SLC7A2 | 6,31E-95 | 1,21 |  | | KRT8 | | 6,35E-12 | -1,02 |  |
| ENPP2 | 7,34E-95 | 1,17 |  | | MAGT1 | | 6,64E-12 | -1,02 |  |
| RGS19 | 1,32E-94 | 1,10 |  | | EFNA3 | | 8,12E-12 | -1,03 |  |
| PPAP2B | 1,80E-94 | 1,09 |  | | GOLM1 | | 1,03E-11 | -1,03 |  |
| SHANK3 | 3,81E-94 | 1,12 |  | | TC2N | | 1,58E-11 | -1,05 |  |
| SLC7A7 | 5,12E-94 | 1,10 |  | | SORL1 | | 1,62E-11 | -1,03 |  |
| FCGR3B | 8,28E-94 | 1,23 |  | | SLC43A1 | | 1,70E-11 | -1,03 |  |
| OSCAR | 1,52E-93 | 1,14 |  | | ACVR1C | | 1,77E-11 | -1,07 |  |
| RAB3B | 1,52E-93 | 1,20 |  | | SOD1 | | 1,91E-11 | -1,01 |  |
| PLEKHH2 | 3,14E-93 | 1,21 |  | | LPAR2 | | 1,93E-11 | -1,02 |  |
| LPP | 4,80E-93 | 1,08 |  | | GPR153 | | 2,52E-11 | -1,02 |  |
| ITGB3 | 8,81E-93 | 1,16 |  | | UNC93A | | 2,65E-11 | -1,10 |  |
| DYSF | 9,58E-93 | 1,09 |  | | MRAP2 | | 3,50E-11 | -1,06 |  |
| DCBLD2 | 1,44E-92 | 1,15 |  | | EPHB4 | | 4,66E-11 | -1,02 |  |
| SIGLEC10 | 2,91E-92 | 1,13 |  | | ABCC1 | | 7,21E-11 | -1,02 |  |
| ITGB1 | 4,65E-92 | 1,06 |  | | PERP | | 1,07E-10 | -1,02 |  |
| SLC8A1 | 5,13E-92 | 1,15 |  | | PKN2 | | 1,15E-10 | -1,02 |  |
| PDGFB | 7,33E-92 | 1,09 |  | | STYK1 | | 1,25E-10 | -1,05 |  |
| FAM65B | 8,37E-92 | 1,14 |  | | MALL | | 1,38E-10 | -1,03 |  |
| HLA-DMA | 1,22E-91 | 1,12 |  | | PTPRH | | 1,61E-10 | -1,03 |  |
| RAMP3 | 1,68E-91 | 1,08 |  | | WWP1 | | 1,96E-10 | -1,02 |  |
| PAM | 2,05E-91 | 1,09 |  | | NCKAP1 | | 2,21E-10 | -1,03 |  |
| SDPR | 5,41E-91 | 1,20 |  | | PLCE1 | | 2,74E-10 | -1,05 |  |
| PDLIM5 | 5,67E-91 | 1,06 |  | | PLEKHA1 | | 2,97E-10 | -1,02 |  |
| CYTH3 | 6,13E-91 | 1,07 |  | | SLC31A1 | | 3,57E-10 | -1,02 |  |
| CD200 | 1,09E-90 | 1,17 |  | | PIGR | | 7,21E-10 | -1,08 |  |
| PLXNA4 | 1,34E-90 | 1,15 |  | | KCNH8 | | 7,36E-10 | -1,06 |  |
| FGD2 | 2,86E-90 | 1,09 |  | | ALG10B | | 9,32E-10 | -1,04 |  |
| SELP | 5,39E-90 | 1,17 |  | | CEMIP | | 1,10E-09 | -1,05 |  |
| TLR7 | 2,22E-89 | 1,22 |  | | ENO1 | | 1,10E-09 | -1,01 |  |
| CSF3R | 2,81E-89 | 1,15 |  | | LRP6 | | 1,38E-09 | -1,02 |  |
| EHD3 | 1,33E-88 | 1,09 |  | | MAP2K2 | | 2,25E-09 | -1,01 |  |
| S100A13 | 1,74E-88 | 1,09 |  | | TM7SF2 | | 2,40E-09 | -1,03 |  |
| GPM6A | 3,79E-88 | 1,50 |  | | WNT4 | | 3,33E-09 | -1,04 |  |
| PLSCR4 | 5,41E-88 | 1,12 |  | | TMEM8B | | 4,83E-09 | -1,02 |  |
| ST3GAL5 | 7,60E-88 | 1,11 |  | | CANT1 | | 4,91E-09 | -1,02 |  |
| RELL1 | 1,24E-87 | 1,08 |  | | ACPP | | 6,09E-09 | -1,05 |  |
| PRKAR2B | 1,64E-87 | 1,20 |  | | RP2 | | 6,30E-09 | -1,03 |  |
| RAB23 | 3,02E-87 | 1,12 |  | | TBCD | | 6,87E-09 | -1,02 |  |
| CHL1 | 3,93E-87 | 1,27 |  | | STXBP3 | | 8,41E-09 | -1,02 |  |
| DLG4 | 7,44E-87 | 1,11 |  | | TDG | | 8,52E-09 | -1,02 |  |
| ADORA3 | 1,23E-86 | 1,16 |  | | CCNT2 | | 1,14E-08 | -1,02 |  |
| SLC27A1 | 1,52E-86 | 1,08 |  | | ATP13A2 | | 1,14E-08 | -1,02 |  |
| TLN1 | 1,99E-86 | 1,08 |  | | NEDD4 | | 1,23E-08 | -1,03 |  |
| PRIMA1 | 6,46E-86 | 1,24 |  | | SIAH1 | | 1,25E-08 | -1,02 |  |
| EDNRA | 6,52E-86 | 1,17 |  | | CLDN23 | | 1,33E-08 | -1,05 |  |
| CADM1 | 7,50E-86 | 1,17 |  | | ATP1B1 | | 3,89E-08 | -1,02 |  |
| STC1 | 1,05E-85 | 1,16 |  | | FZD3 | | 5,38E-08 | -1,05 |  |
| CLEC4A | 1,43E-85 | 1,16 |  | | PCSK9 | | 5,96E-08 | -1,05 |  |
| MYO1G | 1,60E-85 | 1,11 |  | | RASL10B | | 1,24E-07 | -1,04 |  |
| STAC | 1,61E-85 | 1,16 |  | | CDC42EP5 | | 1,61E-07 | -1,03 |  |
| KCND3 | 1,93E-85 | 1,25 |  | | XPNPEP2 | | 2,03E-07 | -1,06 |  |
| CD72 | 2,45E-85 | 1,15 |  | | PIK3C2A | | 2,33E-07 | -1,02 |  |
| RHOH | 4,61E-85 | 1,15 |  | | ENTPD2 | | 2,41E-07 | -1,03 |  |
| P2RY8 | 1,09E-84 | 1,13 |  | | CAT | | 2,67E-07 | -1,02 |  |
| NOTCH4 | 2,02E-84 | 1,07 |  | | SLC9A2 | | 3,09E-07 | -1,05 |  |
| FES | 2,13E-84 | 1,09 |  | | RAPGEF6 | | 3,09E-07 | -1,02 |  |
| SIDT2 | 2,41E-84 | 1,06 |  | | PHKB | | 3,75E-07 | -1,02 |  |
| IGSF6 | 5,24E-84 | 1,18 |  | | ART3 | | 4,38E-07 | -1,07 |  |
| RRAS | 9,33E-84 | 1,06 |  | | CLCA4 | | 5,99E-07 | -1,13 |  |
| IL6 | 1,99E-83 | 1,25 |  | | NMT1 | | 7,89E-07 | -1,01 |  |
| ATP10D | 3,01E-83 | 1,12 |  | | SLC17A4 | | 8,08E-07 | -1,05 |  |
| RILPL1 | 3,60E-83 | 1,08 |  | | PRKAA1 | | 9,08E-07 | -1,02 |  |
| SLC2A5 | 3,63E-83 | 1,16 |  | |  | |  |  |  |
| DIRAS3 | 5,51E-83 | 1,20 |  | |  | |  |  |  |
| PIK3AP1 | 6,50E-83 | 1,11 |  | |  | |  |  |  |
| GNAL | 1,44E-82 | 1,16 |  | |  | |  |  |  |
| CD59 | 2,44E-82 | 1,05 |  | |  | |  |  |  |
| EMP1 | 2,47E-82 | 1,10 |  | |  | |  |  |  |
| PCDHB4 | 2,51E-82 | 1,15 |  | |  | |  |  |  |
| LIFR | 4,98E-82 | 1,19 |  | |  | |  |  |  |
| SERINC1 | 5,06E-82 | 1,05 |  | |  | |  |  |  |
| PJA2 | 2,75E-81 | 1,07 |  | |  | |  |  |  |
| ELMO1 | 2,90E-81 | 1,14 |  | |  | |  |  |  |
| CMKLR1 | 3,09E-81 | 1,11 |  | |  | |  |  |  |
| SLC26A10 | 4,15E-81 | 1,12 |  | |  | |  |  |  |
| CD33 | 7,02E-81 | 1,12 |  | |  | |  |  |  |
| ADAM23 | 8,20E-81 | 1,18 |  | |  | |  |  |  |
| KCNN3 | 1,75E-80 | 1,14 |  | |  | |  |  |  |
| PRKG1 | 2,13E-80 | 1,14 |  | |  | |  |  |  |
| IL1RN | 2,95E-79 | 1,18 |  | |  | |  |  |  |
| GPR34 | 4,18E-79 | 1,19 |  | |  | |  |  |  |
| NFKBIA | 5,05E-79 | 1,07 |  | |  | |  |  |  |
| CD83 | 6,02E-79 | 1,08 |  | |  | |  |  |  |
| GPC1 | 8,67E-79 | 1,09 |  | |  | |  |  |  |
| PRNP | 2,01E-78 | 1,10 |  | |  | |  |  |  |
| GPR161 | 6,35E-78 | 1,12 |  | |  | |  |  |  |
| FLT4 | 1,05E-77 | 1,10 |  | |  | |  |  |  |
| ARRDC3 | 1,30E-77 | 1,08 |  | |  | |  |  |  |
| GPM6B | 1,97E-77 | 1,19 |  | |  | |  |  |  |
| PREX1 | 2,32E-77 | 1,08 |  | |  | |  |  |  |
| NGFR | 2,89E-77 | 1,16 |  | |  | |  |  |  |
| EFNB3 | 3,35E-77 | 1,12 |  | |  | |  |  |  |
| IL16 | 4,29E-77 | 1,10 |  | |  | |  |  |  |
| SIRPA | 4,66E-77 | 1,11 |  | |  | |  |  |  |
| SWAP70 | 5,00E-77 | 1,08 |  | |  | |  |  |  |
| CD48 | 6,80E-77 | 1,13 |  | |  | |  |  |  |
| HLA-DQB1 | 6,81E-77 | 1,13 |  | |  | |  |  |  |
| FAT3 | 7,90E-77 | 1,17 |  | |  | |  |  |  |
| ADRB2 | 1,18E-76 | 1,18 |  | |  | |  |  |  |
| PPP1R16B | 3,47E-76 | 1,12 |  | |  | |  |  |  |
| JAK3 | 3,65E-76 | 1,10 |  | |  | |  |  |  |
| PLCL1 | 4,20E-76 | 1,15 |  | |  | |  |  |  |
| ITGAL | 4,24E-76 | 1,15 |  | |  | |  |  |  |
| ADGRE2 | 5,43E-76 | 1,12 |  | |  | |  |  |  |
| ATP1A2 | 8,44E-76 | 1,31 |  | |  | |  |  |  |
| PLVAP | 2,49E-75 | 1,07 |  | |  | |  |  |  |
| CLEC4E | 3,55E-75 | 1,18 |  | |  | |  |  |  |
| LRP1 | 7,28E-74 | 1,06 |  | |  | |  |  |  |
| APBA2 | 8,71E-74 | 1,11 |  | |  | |  |  |  |
| ACVR1 | 1,13E-73 | 1,06 |  | |  | |  |  |  |
| SHC4 | 2,38E-73 | 1,14 |  | |  | |  |  |  |
| RTN2 | 4,34E-73 | 1,10 |  | |  | |  |  |  |
| PGM5 | 1,00E-72 | 1,25 |  | |  | |  |  |  |
| MAGI2 | 1,04E-72 | 1,15 |  | |  | |  |  |  |
| NISCH | 1,85E-72 | 1,05 |  | |  | |  |  |  |
| DTNA | 3,49E-72 | 1,21 |  | |  | |  |  |  |
| CACNA2D3 | 8,27E-72 | 1,14 |  | |  | |  |  |  |
| DYNC2H1 | 1,05E-71 | 1,13 |  | |  | |  |  |  |
| ADAM19 | 2,16E-71 | 1,09 |  | |  | |  |  |  |
| MR1 | 2,96E-71 | 1,14 |  | |  | |  |  |  |
| HIP1 | 3,55E-71 | 1,10 |  | |  | |  |  |  |
| RASGRF2 | 7,20E-71 | 1,10 |  | |  | |  |  |  |
| HMOX1 | 7,57E-71 | 1,10 |  | |  | |  |  |  |
| HAS1 | 1,66E-70 | 1,17 |  | |  | |  |  |  |
| ITGA1 | 3,10E-70 | 1,16 |  | |  | |  |  |  |
| LPXN | 5,82E-70 | 1,10 |  | |  | |  |  |  |
| MAPK10 | 1,41E-69 | 1,11 |  | |  | |  |  |  |
| PTGER2 | 1,47E-69 | 1,14 |  | |  | |  |  |  |
| ADD1 | 1,91E-69 | 1,05 |  | |  | |  |  |  |
| GBP1 | 2,03E-69 | 1,13 |  | |  | |  |  |  |
| SLCO2B1 | 3,05E-69 | 1,07 |  | |  | |  |  |  |
| HSPB1 | 6,47E-69 | 1,07 |  | |  | |  |  |  |
| AQP1 | 1,10E-68 | 1,13 |  | |  | |  |  |  |
| PRCP | 1,17E-68 | 1,05 |  | |  | |  |  |  |
| ROBO3 | 1,57E-68 | 1,10 |  | |  | |  |  |  |
| CYSLTR1 | 1,75E-68 | 1,15 |  | |  | |  |  |  |
| BTN3A3 | 1,94E-68 | 1,10 |  | |  | |  |  |  |
| TBC1D10C | 2,34E-68 | 1,11 |  | |  | |  |  |  |
| BICD2 | 3,80E-68 | 1,06 |  | |  | |  |  |  |
| CTSG | 4,15E-68 | 1,19 |  | |  | |  |  |  |
| GPRC5B | 4,68E-68 | 1,09 |  | |  | |  |  |  |
| LRRFIP1 | 1,03E-67 | 1,08 |  | |  | |  |  |  |
| ADCY4 | 1,03E-67 | 1,08 |  | |  | |  |  |  |
| ZYX | 1,29E-67 | 1,06 |  | |  | |  |  |  |
| SNAP25 | 1,59E-67 | 1,25 |  | |  | |  |  |  |
| LRMP | 2,24E-67 | 1,15 |  | |  | |  |  |  |
| GPR4 | 3,13E-67 | 1,08 |  | |  | |  |  |  |
| CACNA1H | 5,42E-67 | 1,09 |  | |  | |  |  |  |
| BST2 | 1,00E-66 | 1,13 |  | |  | |  |  |  |
| ADORA2A | 1,04E-66 | 1,08 |  | |  | |  |  |  |
| FZD8 | 1,65E-66 | 1,10 |  | |  | |  |  |  |
| DNM1 | 1,72E-66 | 1,12 |  | |  | |  |  |  |
| EFNA5 | 2,14E-66 | 1,15 |  | |  | |  |  |  |
| RAMP2 | 2,89E-66 | 1,09 |  | |  | |  |  |  |
| TSPAN18 | 7,06E-66 | 1,10 |  | |  | |  |  |  |
| PDE4D | 1,34E-65 | 1,10 |  | |  | |  |  |  |
| KDR | 1,47E-65 | 1,09 |  | |  | |  |  |  |
| PLAUR | 2,69E-65 | 1,08 |  | |  | |  |  |  |
| GNAI1 | 3,68E-64 | 1,14 |  | |  | |  |  |  |
| CPE | 6,39E-64 | 1,17 |  | |  | |  |  |  |
| MARCKS | 1,06E-63 | 1,05 |  | |  | |  |  |  |
| WNT2 | 1,39E-63 | 1,12 |  | |  | |  |  |  |
| SGCB | 1,77E-63 | 1,08 |  | |  | |  |  |  |
| APBB1 | 1,97E-63 | 1,08 |  | |  | |  |  |  |
| GPR27 | 6,38E-63 | 1,20 |  | |  | |  |  |  |
| CNR1 | 7,42E-63 | 1,26 |  | |  | |  |  |  |
| CA11 | 1,14E-62 | 1,10 |  | |  | |  |  |  |
| PENK | 1,16E-62 | 1,24 |  | |  | |  |  |  |
| CYLD | 1,91E-62 | 1,07 |  | |  | |  |  |  |
| C16ORF62 | 4,12E-62 | 1,09 |  | |  | |  |  |  |
| LTB | 4,57E-62 | 1,09 |  | |  | |  |  |  |
| ZBTB16 | 1,18E-61 | 1,19 |  | |  | |  |  |  |
| FNDC4 | 2,52E-61 | 1,07 |  | |  | |  |  |  |
| EPHA4 | 2,57E-61 | 1,19 |  | |  | |  |  |  |
| DISP1 | 3,05E-61 | 1,09 |  | |  | |  |  |  |
| LEPR | 1,53E-60 | 1,14 |  | |  | |  |  |  |
| ARHGEF4 | 3,74E-60 | 1,15 |  | |  | |  |  |  |
| P2RY14 | 3,90E-60 | 1,19 |  | |  | |  |  |  |
| VAV1 | 5,97E-60 | 1,09 |  | |  | |  |  |  |
| ADAM28 | 8,04E-60 | 1,16 |  | |  | |  |  |  |
| ROBO1 | 8,71E-60 | 1,11 |  | |  | |  |  |  |
| GAPT | 1,46E-59 | 1,20 |  | |  | |  |  |  |
| RGS17 | 1,93E-59 | 1,13 |  | |  | |  |  |  |
| PLIN4 | 2,61E-59 | 1,17 |  | |  | |  |  |  |
| GNG7 | 5,10E-59 | 1,10 |  | |  | |  |  |  |
| FLT1 | 5,60E-59 | 1,09 |  | |  | |  |  |  |
| PTGS2 | 1,03E-58 | 1,23 |  | |  | |  |  |  |
| CORIN | 1,11E-58 | 1,20 |  | |  | |  |  |  |
| PTGFR | 1,76E-58 | 1,18 |  | |  | |  |  |  |
| CD81 | 2,08E-58 | 1,03 |  | |  | |  |  |  |
| PIM1 | 2,24E-58 | 1,08 |  | |  | |  |  |  |
| PSD | 2,92E-58 | 1,12 |  | |  | |  |  |  |
| RASGRP1 | 3,76E-58 | 1,15 |  | |  | |  |  |  |
| LY6H | 4,48E-58 | 1,22 |  | |  | |  |  |  |
| ITSN1 | 7,64E-58 | 1,08 |  | |  | |  |  |  |
| ACSL1 | 1,67E-57 | 1,10 |  | |  | |  |  |  |
| CD28 | 4,36E-57 | 1,14 |  | |  | |  |  |  |
| SLC41A1 | 4,65E-57 | 1,07 |  | |  | |  |  |  |
| CD99L2 | 1,75E-56 | 1,06 |  | |  | |  |  |  |
| CD99 | 2,11E-56 | 1,05 |  | |  | |  |  |  |
| STX2 | 2,30E-56 | 1,07 |  | |  | |  |  |  |
| SCN9A | 3,90E-56 | 1,16 |  | |  | |  |  |  |
| WDFY3 | 4,48E-56 | 1,07 |  | |  | |  |  |  |
| PTCHD1 | 1,23E-55 | 1,24 |  | |  | |  |  |  |
| IL11RA | 2,72E-55 | 1,08 |  | |  | |  |  |  |
| ROCK1 | 7,08E-55 | 1,06 |  | |  | |  |  |  |
| CLIC6 | 7,13E-55 | 1,16 |  | |  | |  |  |  |
| DST | 8,09E-55 | 1,07 |  | |  | |  |  |  |
| RHOB | 1,68E-54 | 1,06 |  | |  | |  |  |  |
| GFRA1 | 2,77E-54 | 1,14 |  | |  | |  |  |  |
| GNAO1 | 2,96E-54 | 1,11 |  | |  | |  |  |  |
| P2RY6 | 4,09E-54 | 1,07 |  | |  | |  |  |  |
| P2RY13 | 4,77E-54 | 1,15 |  | |  | |  |  |  |
| SLC2A10 | 8,04E-54 | 1,13 |  | |  | |  |  |  |
| PIK3CG | 9,20E-54 | 1,13 |  | |  | |  |  |  |
| HLA-E | 1,41E-53 | 1,04 |  | |  | |  |  |  |
| SLC2A6 | 2,57E-53 | 1,08 |  | |  | |  |  |  |
| ENPEP | 5,18E-53 | 1,14 |  | |  | |  |  |  |
| SPIRE1 | 1,24E-52 | 1,09 |  | |  | |  |  |  |
| VAMP2 | 1,81E-52 | 1,06 |  | |  | |  |  |  |
| TMEM173 | 3,51E-52 | 1,06 |  | |  | |  |  |  |
| IL7R | 6,76E-52 | 1,12 |  | |  | |  |  |  |
| TACR2 | 9,53E-52 | 1,17 |  | |  | |  |  |  |
| SAMHD1 | 1,39E-51 | 1,07 |  | |  | |  |  |  |
| RAP1A | 2,48E-51 | 1,04 |  | |  | |  |  |  |
| CD8A | 3,80E-51 | 1,12 |  | |  | |  |  |  |
| LAT | 4,25E-51 | 1,08 |  | |  | |  |  |  |
| AMICA1 | 4,61E-51 | 1,08 |  | |  | |  |  |  |
| MS4A2 | 1,40E-50 | 1,16 |  | |  | |  |  |  |
| LPL | 1,72E-50 | 1,15 |  | |  | |  |  |  |
| PCDHB16 | 1,82E-50 | 1,21 |  | |  | |  |  |  |
| ITK | 2,73E-50 | 1,15 |  | |  | |  |  |  |
| IGDCC4 | 3,85E-50 | 1,12 |  | |  | |  |  |  |
| CX3CR1 | 7,10E-50 | 1,16 |  | |  | |  |  |  |
| SCUBE3 | 8,59E-50 | 1,16 |  | |  | |  |  |  |
| SGCA | 9,60E-50 | 1,13 |  | |  | |  |  |  |
| FER | 1,31E-49 | 1,10 |  | |  | |  |  |  |
| CDON | 1,86E-49 | 1,11 |  | |  | |  |  |  |
| SORBS1 | 3,33E-49 | 1,10 |  | |  | |  |  |  |
| CD3E | 4,20E-49 | 1,09 |  | |  | |  |  |  |
| RGS5 | 7,66E-49 | 1,09 |  | |  | |  |  |  |
| OR51E2 | 1,35E-48 | 1,18 |  | |  | |  |  |  |
| LSAMP | 1,66E-48 | 1,11 |  | |  | |  |  |  |
| CPM | 2,34E-48 | 1,12 |  | |  | |  |  |  |
| FAT4 | 2,70E-48 | 1,11 |  | |  | |  |  |  |
| ITGA4 | 3,20E-48 | 1,13 |  | |  | |  |  |  |
| CD274 | 7,81E-48 | 1,13 |  | |  | |  |  |  |
| DMPK | 9,10E-48 | 1,06 |  | |  | |  |  |  |
| STK17A | 9,31E-48 | 1,07 |  | |  | |  |  |  |
| PICALM | 9,52E-48 | 1,04 |  | |  | |  |  |  |
| CD1C | 1,00E-47 | 1,11 |  | |  | |  |  |  |
| FZD7 | 1,28E-47 | 1,12 |  | |  | |  |  |  |
| GHR | 2,34E-47 | 1,21 |  | |  | |  |  |  |
| ITM2B | 2,61E-47 | 1,04 |  | |  | |  |  |  |
| LRRC8A | 4,35E-47 | 1,04 |  | |  | |  |  |  |
| FCER1A | 1,11E-46 | 1,19 |  | |  | |  |  |  |
| LRP12 | 1,15E-46 | 1,12 |  | |  | |  |  |  |
| SCN4B | 2,99E-46 | 1,11 |  | |  | |  |  |  |
| FGFR2 | 6,87E-46 | 1,14 |  | |  | |  |  |  |
| PTH1R | 1,10E-45 | 1,10 |  | |  | |  |  |  |
| SLMAP | 1,79E-45 | 1,08 |  | |  | |  |  |  |
| CD1D | 2,93E-45 | 1,11 |  | |  | |  |  |  |
| VAMP1 | 3,13E-45 | 1,10 |  | |  | |  |  |  |
| AIF1L | 3,13E-45 | 1,12 |  | |  | |  |  |  |
| SLC47A1 | 3,68E-45 | 1,12 |  | |  | |  |  |  |
| NFAM1 | 3,82E-45 | 1,06 |  | |  | |  |  |  |
| PLS3 | 4,00E-45 | 1,05 |  | |  | |  |  |  |
| MYH9 | 4,17E-45 | 1,04 |  | |  | |  |  |  |
| CD2 | 5,60E-45 | 1,10 |  | |  | |  |  |  |
| RAB33A | 8,73E-45 | 1,09 |  | |  | |  |  |  |
| CDH6 | 1,17E-44 | 1,08 |  | |  | |  |  |  |
| IFNAR2 | 1,18E-44 | 1,04 |  | |  | |  |  |  |
| SHC2 | 1,46E-44 | 1,08 |  | |  | |  |  |  |
| TIGIT | 1,62E-44 | 1,11 |  | |  | |  |  |  |
| DGKA | 2,02E-44 | 1,06 |  | |  | |  |  |  |
| CD40 | 3,78E-44 | 1,07 |  | |  | |  |  |  |
| BIN2 | 3,79E-44 | 1,07 |  | |  | |  |  |  |
| BST1 | 9,43E-44 | 1,11 |  | |  | |  |  |  |
| ARL6IP5 | 9,74E-44 | 1,04 |  | |  | |  |  |  |
| TNFRSF9 | 1,27E-43 | 1,11 |  | |  | |  |  |  |
| RGS18 | 1,56E-43 | 1,15 |  | |  | |  |  |  |
| RAC2 | 1,62E-43 | 1,06 |  | |  | |  |  |  |
| SCEL | 2,34E-43 | 1,30 |  | |  | |  |  |  |
| ABTB1 | 4,00E-43 | 1,05 |  | |  | |  |  |  |
| CPEB4 | 5,96E-43 | 1,07 |  | |  | |  |  |  |
| BDKRB1 | 6,67E-43 | 1,09 |  | |  | |  |  |  |
| IL2RB | 7,32E-43 | 1,07 |  | |  | |  |  |  |
| RNF146 | 7,81E-43 | 1,05 |  | |  | |  |  |  |
| NPR1 | 8,58E-43 | 1,06 |  | |  | |  |  |  |
| LITAF | 9,80E-43 | 1,04 |  | |  | |  |  |  |
| ST6GALNAC6 | 9,94E-43 | 1,08 |  | |  | |  |  |  |
| SGMS2 | 2,25E-42 | 1,08 |  | |  | |  |  |  |
| STK17B | 2,41E-42 | 1,07 |  | |  | |  |  |  |
| CCR7 | 4,58E-42 | 1,13 |  | |  | |  |  |  |
| SLC16A6 | 9,91E-42 | 1,12 |  | |  | |  |  |  |
| JAK2 | 1,97E-41 | 1,08 |  | |  | |  |  |  |
| NLRX1 | 2,19E-41 | 1,05 |  | |  | |  |  |  |
| LYNX1 | 2,59E-41 | 1,08 |  | |  | |  |  |  |
| HLA-DOB | 3,35E-41 | 1,10 |  | |  | |  |  |  |
| IL2RA | 4,28E-41 | 1,10 |  | |  | |  |  |  |
| SLAMF7 | 5,67E-41 | 1,15 |  | |  | |  |  |  |
| CLEC10A | 7,74E-41 | 1,12 |  | |  | |  |  |  |
| CORO1C | 9,66E-41 | 1,04 |  | |  | |  |  |  |
| ERRFI1 | 1,08E-40 | 1,07 |  | |  | |  |  |  |
| CD300C | 1,33E-40 | 1,08 |  | |  | |  |  |  |
| ADRB1 | 1,65E-40 | 1,18 |  | |  | |  |  |  |
| CSTA | 1,71E-40 | 1,14 |  | |  | |  |  |  |
| PTPN22 | 1,93E-40 | 1,11 |  | |  | |  |  |  |
| FAM26F | 1,94E-40 | 1,13 |  | |  | |  |  |  |
| RASD1 | 2,93E-40 | 1,10 |  | |  | |  |  |  |
| CD80 | 5,25E-40 | 1,10 |  | |  | |  |  |  |
| NCF4 | 8,32E-40 | 1,10 |  | |  | |  |  |  |
| KCND1 | 1,53E-39 | 1,07 |  | |  | |  |  |  |
| GRK5 | 2,97E-39 | 1,06 |  | |  | |  |  |  |
| FAM155A | 4,97E-39 | 1,15 |  | |  | |  |  |  |
| TCIRG1 | 7,93E-39 | 1,05 |  | |  | |  |  |  |
| PTPRCAP | 9,36E-39 | 1,07 |  | |  | |  |  |  |
| LZTS2 | 1,24E-38 | 1,05 |  | |  | |  |  |  |
| PIK3IP1 | 1,71E-38 | 1,04 |  | |  | |  |  |  |
| NLGN4Y | 2,44E-38 | 1,21 |  | |  | |  |  |  |
| MIB1 | 3,24E-38 | 1,06 |  | |  | |  |  |  |
| ENTPD3 | 4,29E-38 | 1,12 |  | |  | |  |  |  |
| NDRG4 | 7,73E-38 | 1,07 |  | |  | |  |  |  |
| EHBP1 | 1,27E-37 | 1,05 |  | |  | |  |  |  |
| NCAM1 | 1,32E-37 | 1,11 |  | |  | |  |  |  |
| RAB27A | 1,48E-37 | 1,08 |  | |  | |  |  |  |
| CD247 | 1,83E-37 | 1,08 |  | |  | |  |  |  |
| C1QTNF1 | 2,12E-37 | 1,05 |  | |  | |  |  |  |
| TMEM108 | 2,51E-37 | 1,08 |  | |  | |  |  |  |
| LPAR6 | 4,93E-37 | 1,08 |  | |  | |  |  |  |
| L1CAM | 1,36E-36 | 1,17 |  | |  | |  |  |  |
| CAPN2 | 2,09E-36 | 1,04 |  | |  | |  |  |  |
| CKAP4 | 2,28E-36 | 1,04 |  | |  | |  |  |  |
| HGSNAT | 3,89E-36 | 1,04 |  | |  | |  |  |  |
| CD79B | 4,14E-36 | 1,09 |  | |  | |  |  |  |
| CD27 | 4,30E-36 | 1,10 |  | |  | |  |  |  |
| SIGLEC5 | 4,82E-36 | 1,09 |  | |  | |  |  |  |
| GPR171 | 1,22E-35 | 1,14 |  | |  | |  |  |  |
| RAB9B | 1,46E-35 | 1,10 |  | |  | |  |  |  |
| NAIP | 1,65E-35 | 1,08 |  | |  | |  |  |  |
| NEDD9 | 1,79E-35 | 1,06 |  | |  | |  |  |  |
| BTN3A1 | 2,36E-35 | 1,05 |  | |  | |  |  |  |
| VLDLR | 2,55E-35 | 1,13 |  | |  | |  |  |  |
| KCNK3 | 2,63E-35 | 1,10 |  | |  | |  |  |  |
| ATP6V0D2 | 5,38E-35 | 1,09 |  | |  | |  |  |  |
| EPHB6 | 1,30E-34 | 1,09 |  | |  | |  |  |  |
| ARHGEF2 | 1,31E-34 | 1,04 |  | |  | |  |  |  |
| SULF2 | 1,66E-34 | 1,06 |  | |  | |  |  |  |
| P2RX1 | 2,98E-34 | 1,08 |  | |  | |  |  |  |
| ADCY5 | 5,20E-34 | 1,10 |  | |  | |  |  |  |
| KIAA0922 | 5,24E-34 | 1,07 |  | |  | |  |  |  |
| SCN3B | 1,19E-33 | 1,11 |  | |  | |  |  |  |
| S100A12 | 1,27E-33 | 1,17 |  | |  | |  |  |  |
| LCK | 1,38E-33 | 1,09 |  | |  | |  |  |  |
| ADGRG3 | 1,61E-33 | 1,08 |  | |  | |  |  |  |
| ANGPT2 | 1,63E-33 | 1,08 |  | |  | |  |  |  |
| MS4A1 | 2,54E-33 | 1,21 |  | |  | |  |  |  |
| FCRL3 | 2,80E-33 | 1,14 |  | |  | |  |  |  |
| ARL4D | 3,23E-33 | 1,06 |  | |  | |  |  |  |
| CXCL10 | 4,56E-33 | 1,13 |  | |  | |  |  |  |
| OSBPL6 | 5,02E-33 | 1,12 |  | |  | |  |  |  |
| CD22 | 5,51E-33 | 1,09 |  | |  | |  |  |  |
| BMPR2 | 6,11E-33 | 1,05 |  | |  | |  |  |  |
| CD55 | 7,01E-33 | 1,06 |  | |  | |  |  |  |
| GPBAR1 | 1,13E-32 | 1,06 |  | |  | |  |  |  |
| RASAL3 | 1,91E-32 | 1,06 |  | |  | |  |  |  |
| GAA | 2,36E-32 | 1,05 |  | |  | |  |  |  |
| DAAM1 | 3,07E-32 | 1,05 |  | |  | |  |  |  |
| PTPN13 | 3,87E-32 | 1,15 |  | |  | |  |  |  |
| SLCO2A1 | 6,51E-32 | 1,07 |  | |  | |  |  |  |
| P2RY10 | 1,75E-31 | 1,10 |  | |  | |  |  |  |
| NT5E | 2,74E-31 | 1,09 |  | |  | |  |  |  |
| HHIP | 2,93E-31 | 1,12 |  | |  | |  |  |  |
| TMEM25 | 3,00E-31 | 1,06 |  | |  | |  |  |  |
| GRASP | 3,04E-31 | 1,05 |  | |  | |  |  |  |
| CD96 | 7,82E-31 | 1,09 |  | |  | |  |  |  |
| KCNS3 | 1,18E-30 | 1,09 |  | |  | |  |  |  |
| ITGB7 | 1,19E-30 | 1,06 |  | |  | |  |  |  |
| GNAQ | 4,16E-30 | 1,04 |  | |  | |  |  |  |
| CASP4 | 5,99E-30 | 1,05 |  | |  | |  |  |  |
| AQP3 | 1,09E-29 | 1,10 |  | |  | |  |  |  |
| ICAM2 | 1,24E-29 | 1,05 |  | |  | |  |  |  |
| CXCL9 | 1,43E-29 | 1,12 |  | |  | |  |  |  |
| S100A9 | 1,56E-29 | 1,09 |  | |  | |  |  |  |
| CFLAR | 2,00E-29 | 1,04 |  | |  | |  |  |  |
| NAALADL1 | 3,22E-29 | 1,06 |  | |  | |  |  |  |
| SLAMF1 | 4,61E-29 | 1,08 |  | |  | |  |  |  |
| IL6R | 2,12E-28 | 1,08 |  | |  | |  |  |  |
| FAS | 2,30E-28 | 1,08 |  | |  | |  |  |  |
| TRAT1 | 2,71E-28 | 1,13 |  | |  | |  |  |  |
| SENP7 | 3,73E-28 | 1,06 |  | |  | |  |  |  |
| IL1R2 | 5,72E-28 | 1,11 |  | |  | |  |  |  |
| FGF13 | 7,58E-28 | 1,12 |  | |  | |  |  |  |
| PNOC | 9,41E-28 | 1,11 |  | |  | |  |  |  |
| SLC39A6 | 1,07E-27 | 1,05 |  | |  | |  |  |  |
| DAPP1 | 1,75E-27 | 1,10 |  | |  | |  |  |  |
| SLC16A4 | 3,23E-27 | 1,10 |  | |  | |  |  |  |
| LAX1 | 1,21E-26 | 1,09 |  | |  | |  |  |  |
| BMF | 1,36E-26 | 1,04 |  | |  | |  |  |  |
| LY9 | 1,52E-26 | 1,09 |  | |  | |  |  |  |
| IL3RA | 2,50E-26 | 1,05 |  | |  | |  |  |  |
| NUCB2 | 6,98E-26 | 1,05 |  | |  | |  |  |  |
| ARRDC4 | 8,28E-26 | 1,06 |  | |  | |  |  |  |
| PRF1 | 1,32E-25 | 1,07 |  | |  | |  |  |  |
| UNC5C | 2,33E-25 | 1,07 |  | |  | |  |  |  |
| BEST1 | 2,85E-25 | 1,05 |  | |  | |  |  |  |
| BTN3A2 | 3,33E-25 | 1,05 |  | |  | |  |  |  |
| PSD3 | 4,77E-25 | 1,07 |  | |  | |  |  |  |
| B2M | 1,02E-24 | 1,02 |  | |  | |  |  |  |
| F5 | 1,16E-24 | 1,13 |  | |  | |  |  |  |
| ANPEP | 1,99E-24 | 1,12 |  | |  | |  |  |  |
| SLC4A3 | 2,20E-24 | 1,07 |  | |  | |  |  |  |
| EPHA7 | 2,35E-24 | 1,14 |  | |  | |  |  |  |
| PTPN7 | 3,00E-24 | 1,07 |  | |  | |  |  |  |
| DKK1 | 3,18E-24 | 1,18 |  | |  | |  |  |  |
| NCOA1 | 5,70E-24 | 1,03 |  | |  | |  |  |  |
| SNX9 | 2,52E-23 | 1,03 |  | |  | |  |  |  |
| PCDH18 | 2,69E-23 | 1,06 |  | |  | |  |  |  |
| SYT17 | 2,75E-23 | 1,08 |  | |  | |  |  |  |
| DAB2 | 3,59E-23 | 1,04 |  | |  | |  |  |  |
| CD3D | 4,60E-23 | 1,06 |  | |  | |  |  |  |
| ZAP70 | 4,62E-23 | 1,07 |  | |  | |  |  |  |
| STRN3 | 5,93E-23 | 1,05 |  | |  | |  |  |  |
| TGFBR2 | 6,02E-23 | 1,03 |  | |  | |  |  |  |
| SMPD1 | 6,50E-23 | 1,04 |  | |  | |  |  |  |
| RGS13 | 6,55E-23 | 1,16 |  | |  | |  |  |  |
| ADGRE1 | 8,58E-23 | 1,07 |  | |  | |  |  |  |
| FCRL5 | 8,80E-23 | 1,11 |  | |  | |  |  |  |
| SEMA4C | 1,51E-22 | 1,03 |  | |  | |  |  |  |
| TLR4 | 3,56E-22 | 1,10 |  | |  | |  |  |  |
| SDCBP | 3,95E-22 | 1,03 |  | |  | |  |  |  |
| PPP3CA | 5,61E-22 | 1,03 |  | |  | |  |  |  |
| SPRED1 | 7,71E-22 | 1,05 |  | |  | |  |  |  |
| ADRA2A | 7,78E-22 | 1,10 |  | |  | |  |  |  |
| PRKAR1A | 1,49E-21 | 1,03 |  | |  | |  |  |  |
| TLR10 | 2,09E-21 | 1,08 |  | |  | |  |  |  |
| PROS1 | 3,42E-21 | 1,07 |  | |  | |  |  |  |
| VNN1 | 3,64E-21 | 1,12 |  | |  | |  |  |  |
| PLEC | 5,36E-21 | 1,04 |  | |  | |  |  |  |
| RAB18 | 8,55E-21 | 1,03 |  | |  | |  |  |  |
| IKZF3 | 2,34E-20 | 1,07 |  | |  | |  |  |  |
| TMEM30A | 3,16E-20 | 1,03 |  | |  | |  |  |  |
| SEMA4A | 3,61E-20 | 1,03 |  | |  | |  |  |  |
| CXCR2 | 1,06E-19 | 1,09 |  | |  | |  |  |  |
| CD19 | 1,18E-19 | 1,08 |  | |  | |  |  |  |
| CDC42EP2 | 1,09E-18 | 1,03 |  | |  | |  |  |  |
| SNTB2 | 2,19E-18 | 1,03 |  | |  | |  |  |  |
| MSLN | 3,22E-18 | 1,13 |  | |  | |  |  |  |
| SPPL2A | 5,53E-18 | 1,03 |  | |  | |  |  |  |
| SCFD1 | 6,24E-18 | 1,03 |  | |  | |  |  |  |
| HLA-F | 8,70E-18 | 1,03 |  | |  | |  |  |  |
| PTGDR | 9,25E-18 | 1,13 |  | |  | |  |  |  |
| NTRK2 | 1,28E-17 | 1,16 |  | |  | |  |  |  |
| SORBS2 | 2,19E-17 | 1,07 |  | |  | |  |  |  |
| ANO6 | 3,14E-17 | 1,03 |  | |  | |  |  |  |
| VIPR2 | 3,66E-17 | 1,05 |  | |  | |  |  |  |
| LY6E | 4,12E-17 | 1,05 |  | |  | |  |  |  |
| RAB29 | 4,21E-17 | 1,04 |  | |  | |  |  |  |
| TNFRSF11B | 5,31E-17 | 1,09 |  | |  | |  |  |  |
| ITGB1BP1 | 6,89E-17 | 1,03 |  | |  | |  |  |  |
| STEAP1 | 9,18E-17 | 1,05 |  | |  | |  |  |  |
| GFRA3 | 1,09E-16 | 1,07 |  | |  | |  |  |  |
| ZDHHC17 | 1,22E-16 | 1,03 |  | |  | |  |  |  |
| IL18R1 | 1,41E-16 | 1,06 |  | |  | |  |  |  |
| SPRR1B | 1,55E-16 | 1,15 |  | |  | |  |  |  |
| PIK3CA | 1,65E-16 | 1,04 |  | |  | |  |  |  |
| RANBP9 | 1,80E-16 | 1,03 |  | |  | |  |  |  |
| HLA-G | 3,02E-16 | 1,03 |  | |  | |  |  |  |
| BDKRB2 | 3,15E-16 | 1,03 |  | |  | |  |  |  |
| CACNB2 | 3,73E-16 | 1,06 |  | |  | |  |  |  |
| HLA-C | 4,52E-16 | 1,03 |  | |  | |  |  |  |
| GNA13 | 4,64E-16 | 1,03 |  | |  | |  |  |  |
| CD38 | 6,70E-16 | 1,07 |  | |  | |  |  |  |
| MAGED1 | 7,70E-16 | 1,02 |  | |  | |  |  |  |
| FMN2 | 9,96E-16 | 1,11 |  | |  | |  |  |  |
| VEZT | 1,29E-15 | 1,03 |  | |  | |  |  |  |
| PLXNB2 | 1,62E-15 | 1,02 |  | |  | |  |  |  |
| BCAM | 3,77E-15 | 1,03 |  | |  | |  |  |  |
| GLDN | 7,01E-15 | 1,08 |  | |  | |  |  |  |
| SERPINE2 | 9,12E-15 | 1,04 |  | |  | |  |  |  |
| FCRL2 | 1,34E-14 | 1,08 |  | |  | |  |  |  |
| SGPP1 | 1,41E-14 | 1,04 |  | |  | |  |  |  |
| SCN3A | 2,20E-14 | 1,08 |  | |  | |  |  |  |
| PVRL2 | 3,75E-14 | 1,02 |  | |  | |  |  |  |
| TJP1 | 4,16E-14 | 1,02 |  | |  | |  |  |  |
| HAS2 | 6,41E-14 | 1,07 |  | |  | |  |  |  |
| C8ORF4 | 6,52E-14 | 1,05 |  | |  | |  |  |  |
| APC | 6,64E-14 | 1,05 |  | |  | |  |  |  |
| SPRR1A | 1,04E-13 | 1,13 |  | |  | |  |  |  |
| TNFRSF19 | 1,07E-13 | 1,11 |  | |  | |  |  |  |
| SCARA5 | 1,21E-13 | 1,08 |  | |  | |  |  |  |
| SIRPG | 1,76E-13 | 1,05 |  | |  | |  |  |  |
| ATP6V1D | 1,99E-13 | 1,03 |  | |  | |  |  |  |
| EBI3 | 2,78E-13 | 1,05 |  | |  | |  |  |  |
| PLEKHA4 | 2,82E-13 | 1,04 |  | |  | |  |  |  |
| ABCG2 | 4,50E-13 | 1,08 |  | |  | |  |  |  |
| SIT1 | 4,71E-13 | 1,05 |  | |  | |  |  |  |
| KLRB1 | 7,58E-13 | 1,06 |  | |  | |  |  |  |
| AGTRAP | 9,76E-13 | 1,02 |  | |  | |  |  |  |
| IFIT5 | 1,32E-12 | 1,04 |  | |  | |  |  |  |
| ALCAM | 1,38E-12 | 1,05 |  | |  | |  |  |  |
| TGM2 | 1,67E-12 | 1,03 |  | |  | |  |  |  |
| DMD | 1,69E-12 | 1,07 |  | |  | |  |  |  |
| EDNRB | 2,04E-12 | 1,06 |  | |  | |  |  |  |
| TNFRSF18 | 2,26E-12 | 1,03 |  | |  | |  |  |  |
| MARK1 | 2,31E-12 | 1,08 |  | |  | |  |  |  |
| GNAZ | 4,41E-12 | 1,04 |  | |  | |  |  |  |
| MALT1 | 1,20E-11 | 1,03 |  | |  | |  |  |  |
| TSPAN32 | 1,50E-11 | 1,05 |  | |  | |  |  |  |
| SLC4A11 | 1,51E-11 | 1,07 |  | |  | |  |  |  |
| RHOBTB1 | 2,05E-11 | 1,04 |  | |  | |  |  |  |
| CD6 | 2,31E-11 | 1,03 |  | |  | |  |  |  |
| ENAH | 2,87E-11 | 1,03 |  | |  | |  |  |  |
| CXCR6 | 3,65E-11 | 1,04 |  | |  | |  |  |  |
| MICB | 3,87E-11 | 1,05 |  | |  | |  |  |  |
| ARL13B | 5,80E-11 | 1,04 |  | |  | |  |  |  |
| NCK1 | 6,43E-10 | 1,02 |  | |  | |  |  |  |
| SGMS1 | 8,48E-10 | 1,04 |  | |  | |  |  |  |
| ZDHHC2 | 9,75E-10 | 1,05 |  | |  | |  |  |  |
| KCNH2 | 1,06E-09 | 1,04 |  | |  | |  |  |  |
| PLA2G2A | 1,10E-09 | 1,08 |  | |  | |  |  |  |
| ASAP1 | 2,76E-09 | 1,03 |  | |  | |  |  |  |
| ITGB8 | 5,02E-09 | 1,04 |  | |  | |  |  |  |
| ADAM9 | 7,62E-09 | 1,02 |  | |  | |  |  |  |
| NPY1R | 9,26E-09 | 1,09 |  | |  | |  |  |  |
| TNFRSF17 | 1,49E-08 | 1,10 |  | |  | |  |  |  |
| KL | 2,84E-08 | 1,07 |  | |  | |  |  |  |
| SNAP23 | 3,06E-08 | 1,02 |  | |  | |  |  |  |
| CAMK2D | 7,06E-08 | 1,02 |  | |  | |  |  |  |
| SDR16C5 | 8,41E-08 | 1,09 |  | |  | |  |  |  |
| RAB27B | 1,08E-07 | 1,05 |  | |  | |  |  |  |
| GPR18 | 1,79E-07 | 1,05 |  | |  | |  |  |  |
| ADA | 2,38E-07 | 1,03 |  | |  | |  |  |  |
| ARID4A | 2,63E-07 | 1,03 |  | |  | |  |  |  |
| RHBDF2 | 2,89E-07 | 1,02 |  | |  | |  |  |  |
| TAB2 | 3,89E-07 | 1,02 |  | |  | |  |  |  |
| SLC41A2 | 5,91E-07 | 1,03 |  | |  | |  |  |  |
| TNFSF10 | 6,51E-07 | 1,03 |  | |  | |  |  |  |

**Table S1 – Differentially expressed cell surface markers in CMS2 versus CMS4 colon cancer.** Differential gene expression analysis between 110 CMS2 and 770 CMS4 tumors in the CMS3232 cohort was performed on the R2: Genomics Analysis and Visualization Platform (http://r2.amc.nl) using genes in the GO term ‘plasma membrane’ only (GO:0005886) and multiple testing correction by False Discovery Rate. Genes with a significantly different (p<0.05) expression between CMS2 and CMS4 tumors are listed.
