# Supplementary material for: Fibroblast activation protein identifies Consensus Molecular Subtype 4 in colorectal cancer and allows its detection by 68Ga-FAPI-PET imaging
Source: Br J Cancer. 2022 Mar 16;127(1):145–55. doi: 10.1038/s41416-022-01748-z (PMC9276750; doi:10.1038/s41416-022-01748-z)
Supplement: Supplementary file 3 — Table S2 [file 41416_2022_1748_MOESM3_ESM.docx]

| **Genes sign. UP in FAP+ stromal cells** | | |  | **Genes sign. DOWN in FAP+ stromal cells** | | |
| --- | --- | --- | --- | --- | --- | --- |
| **hugo** | **corrected p** | **log2 fold change** |  | **hugo** | **corrected p** | **log2 fold change** |
| COL1A1 | 0,00E+00 | 2,43 |  | ADAMDEC1 | 3,43E-74 | -2,94 |
| MMP1 | 3,08E-166 | 2,30 |  | CFD | 0,00E+00 | -1,61 |
| CTHRC1 | 0,00E+00 | 2,25 |  | ADIRF | 0,00E+00 | -1,57 |
| COL3A1 | 0,00E+00 | 2,02 |  | CCL13 | 6,50E-48 | -1,34 |
| SFRP4 | 0,00E+00 | 1,92 |  | IGHA1 | 0,00E+00 | -1,32 |
| COMP | 0,00E+00 | 1,88 |  | CXCL14 | 2,90E-55 | -1,24 |
| COL1A2 | 0,00E+00 | 1,87 |  | CCL2 | 6,00E-97 | -1,20 |
| MMP11 | 1,09E-259 | 1,87 |  | GPX3 | 0,00E+00 | -1,20 |
| BGN | 0,00E+00 | 1,74 |  | FABP5 | 5,96E-115 | -1,18 |
| MMP3 | 1,68E-113 | 1,64 |  | IRF1 | 1,77E-134 | -1,09 |
| POSTN | 0,00E+00 | 1,61 |  | CEBPD | 2,19E-229 | -1,06 |
| THBS2 | 0,00E+00 | 1,52 |  | FXYD1 | 1,40E-171 | -1,05 |
| CST1 | 5,51E-106 | 1,41 |  | GSN | 0,00E+00 | -1,04 |
| INHBA | 0,00E+00 | 1,39 |  | PLAC9 | 3,22E-244 | -1,03 |
| FN1 | 8,62E-206 | 1,38 |  | SOCS3 | 1,40E-132 | -1,00 |
| FAP | 0,00E+00 | 1,37 |  | JUNB | 9,54E-151 | -0,99 |
| SULF1 | 0,00E+00 | 1,35 |  | CRYAB | 1,00E+00 | -0,99 |
| COL5A2 | 0,00E+00 | 1,32 |  | JUN | 6,72E-191 | -0,98 |
| CXCL8 | 5,16E-121 | 1,27 |  | CLU | 4,12E-69 | -0,96 |
| COL10A1 | 0,00E+00 | 1,27 |  | CCL8 | 1,13E-51 | -0,95 |
| SPARC | 0,00E+00 | 1,26 |  | TXNIP | 9,58E-288 | -0,95 |
| COL6A3 | 0,00E+00 | 1,24 |  | LTBP4 | 6,86E-209 | -0,93 |
| COL5A1 | 0,00E+00 | 1,24 |  | ADH1B | 2,57E-113 | -0,91 |
| COL12A1 | 0,00E+00 | 1,21 |  | FABP4 | 9,77E-72 | -0,90 |
| ASPN | 2,31E-150 | 1,18 |  | APOE | 1,16E-07 | -0,89 |
| CHI3L1 | 1,48E-194 | 1,17 |  | ZFP36 | 4,21E-180 | -0,88 |
| AEBP1 | 1,02E-293 | 1,16 |  | SPARCL1 | 2,24E-234 | -0,88 |
| GJB2 | 0,00E+00 | 1,14 |  | JCHAIN | 0,00E+00 | -0,86 |
| MMP14 | 0,00E+00 | 1,14 |  | SOD3 | 2,34E-143 | -0,82 |
| TNFRSF12A | 0,00E+00 | 1,05 |  | ABCA8 | 4,67E-134 | -0,80 |
| COL11A1 | 0,00E+00 | 1,02 |  | IGLC2 | 9,53E-89 | -0,79 |
| TGFBI | 6,29E-198 | 0,99 |  | S100A4 | 1,30E-90 | -0,79 |
| CTSB | 0,00E+00 | 0,98 |  | IGFBP6 | 1,77E-34 | -0,78 |
| CTSK | 7,36E-196 | 0,97 |  | PPP1R14A | 2,99E-136 | -0,78 |
| EPYC | 1,03E-207 | 0,96 |  | FOS | 7,33E-99 | -0,76 |
| TIMP1 | 6,47E-146 | 0,96 |  | RGS5 | 2,45E-23 | -0,75 |
| RCN3 | 0,00E+00 | 0,95 |  | CLDN5 | 1,62E-27 | -0,75 |
| SERPINE1 | 3,62E-158 | 0,94 |  | SEPP1 | 3,58E-227 | -0,74 |
| CXCL1 | 1,46E-26 | 0,94 |  | KLF4 | 9,30E-158 | -0,74 |
| RAB31 | 0,00E+00 | 0,94 |  | MFAP4 | 1,04E-63 | -0,73 |
| ANTXR1 | 0,00E+00 | 0,92 |  | GPM6B | 2,06E-02 | -0,72 |
| SPON2 | 1,05E-177 | 0,91 |  | PLPP3 | 3,98E-138 | -0,72 |
| SERPINH1 | 0,00E+00 | 0,91 |  | CD320 | 6,27E-24 | -0,71 |
| CRABP2 | 5,96E-263 | 0,90 |  | BTG2 | 1,45E-154 | -0,70 |
| MMP2 | 5,46E-226 | 0,89 |  | CD74 | 1,24E-75 | -0,70 |
| LUM | 9,36E-164 | 0,87 |  | DPT | 3,59E-11 | -0,69 |
| C3 | 4,25E-131 | 0,87 |  | GADD45B | 3,97E-68 | -0,69 |
| MFAP2 | 0,00E+00 | 0,86 |  | A2M | 1,67E-182 | -0,69 |
| THY1 | 1,54E-222 | 0,85 |  | CD9 | 5,45E-39 | -0,69 |
| CHPF | 0,00E+00 | 0,85 |  | H3F3B | 8,66E-237 | -0,67 |
| COL8A1 | 0,00E+00 | 0,84 |  | IGKC | 2,99E-227 | -0,67 |
| FTH1 | 1,97E-68 | 0,84 |  | MFAP5 | 3,34E-01 | -0,67 |
| VCAN | 2,34E-204 | 0,84 |  | PDK4 | 2,64E-137 | -0,67 |
| IGFL2 | 4,02E-237 | 0,83 |  | TPPP3 | 1,90E-74 | -0,66 |
| MXRA5 | 0,00E+00 | 0,83 |  | HLA-E | 2,83E-140 | -0,66 |
| GREM1 | 3,41E-253 | 0,82 |  | PTN | 1,89E-88 | -0,65 |
| NBL1 | 7,90E-208 | 0,82 |  | TCF21 | 2,18E-68 | -0,65 |
| MDK | 2,19E-235 | 0,80 |  | BTG1 | 4,01E-165 | -0,64 |
| CDH11 | 0,00E+00 | 0,80 |  | LGI4 | 4,42E-64 | -0,63 |
| PDPN | 1,47E-283 | 0,79 |  | C8orf4 | 1,56E-53 | -0,63 |
| TAGLN | 1,12E-161 | 0,78 |  | TNXB | 1,44E-64 | -0,62 |
| HOPX | 3,12E-225 | 0,77 |  | TM4SF1 | 8,93E-74 | -0,62 |
| TNC | 2,82E-183 | 0,75 |  | ALDH1A1 | 3,09E-30 | -0,62 |
| RARRES2 | 5,66E-126 | 0,75 |  | FHL1 | 8,16E-108 | -0,61 |
| LGALS1 | 1,04E-261 | 0,74 |  | TMEM176B | 3,55E-43 | -0,61 |
| ADAM12 | 0,00E+00 | 0,74 |  | ZFP36L2 | 2,57E-136 | -0,61 |
| SLC16A3 | 0,00E+00 | 0,74 |  | MATN2 | 4,37E-103 | -0,60 |
| HSPA6 | 7,60E-44 | 0,73 |  | MYH11 | 9,53E-12 | -0,59 |
| CXCL6 | 7,22E-76 | 0,73 |  | PCOLCE2 | 5,73E-39 | -0,59 |
| CERCAM | 0,00E+00 | 0,72 |  | RHOB | 1,12E-135 | -0,58 |
| TNFAIP6 | 1,19E-156 | 0,72 |  | MT-ND2 | 3,43E-241 | -0,57 |
| COL6A1 | 1,94E-123 | 0,72 |  | MT-ND3 | 1,72E-131 | -0,57 |
| MXRA8 | 2,54E-220 | 0,71 |  | CXCL12 | 1,07E-111 | -0,57 |
| PKM | 6,39E-219 | 0,71 |  | TFPI | 1,24E-72 | -0,55 |
| PCOLCE | 8,08E-150 | 0,71 |  | SCARA5 | 2,34E-88 | -0,55 |
| P4HB | 5,53E-289 | 0,70 |  | FOSB | 8,61E-61 | -0,54 |
| IGF1 | 2,57E-25 | 0,70 |  | GNG11 | 7,23E-136 | -0,54 |
| SFRP2 | 3,96E-100 | 0,69 |  | SDPR | 4,88E-30 | -0,53 |
| MIF | 1,22E-223 | 0,69 |  | STMN2 | 4,84E-55 | -0,53 |
| RARRES1 | 9,48E-176 | 0,69 |  | APLP2 | 5,08E-126 | -0,52 |
| LOXL2 | 4,34E-257 | 0,69 |  | HLA-DRB1 | 2,92E-28 | -0,52 |
| SOD2 | 3,32E-29 | 0,69 |  | EGR1 | 7,75E-40 | -0,52 |
| HTRA1 | 3,14E-167 | 0,69 |  | MT-ND1 | 1,24E-202 | -0,52 |
| CXCL3 | 8,26E-63 | 0,68 |  | TSC22D3 | 2,36E-86 | -0,52 |
| PLOD2 | 0,00E+00 | 0,67 |  | IER2 | 6,67E-78 | -0,51 |
| ISLR | 5,96E-172 | 0,66 |  | SLPI | 2,43E-04 | -0,50 |
| TPM4 | 5,11E-275 | 0,65 |  | DDX5 | 1,38E-133 | -0,50 |
| FKBP10 | 1,10E-291 | 0,65 |  | PMP22 | 4,27E-17 | -0,50 |
| PRRX1 | 0,00E+00 | 0,65 |  | PLAT | 1,30E-09 | -0,49 |
| PLAU | 2,45E-95 | 0,64 |  | PPP1R15A | 8,68E-55 | -0,49 |
| CALU | 3,18E-245 | 0,63 |  | SRSF5 | 2,15E-146 | -0,48 |
| CNN2 | 0,00E+00 | 0,63 |  | ITM2B | 1,07E-140 | -0,47 |
| GAS1 | 9,27E-242 | 0,63 |  | MT-CYB | 1,55E-169 | -0,47 |
| CLEC11A | 2,13E-181 | 0,63 |  | HAPLN1 | 1,08E-59 | -0,47 |
| PTGDS | 6,83E-46 | 0,63 |  | MT-ATP6 | 1,34E-134 | -0,46 |
| IGHG3 | 1,28E-303 | 0,62 |  | LSP1 | 2,51E-72 | -0,46 |
| TPM2 | 6,50E-171 | 0,62 |  | NDRG2 | 6,01E-77 | -0,46 |
| LOX | 5,54E-235 | 0,61 |  | CD36 | 5,76E-35 | -0,46 |
| OSTC | 8,80E-249 | 0,61 |  | PRNP | 2,93E-12 | -0,45 |
| PDLIM7 | 2,15E-226 | 0,61 |  | HLA-DRA | 1,38E-09 | -0,44 |
| SPHK1 | 0,00E+00 | 0,60 |  | CCNL1 | 3,66E-69 | -0,44 |
| MT2A | 3,11E-19 | 0,60 |  | PROCR | 1,72E-42 | -0,44 |
| CTGF | 1,23E-36 | 0,60 |  | IGHA2 | 1,20E-118 | -0,43 |
| PDLIM3 | 9,73E-153 | 0,59 |  | MT-CO3 | 3,27E-127 | -0,43 |
| HTRA3 | 3,08E-126 | 0,59 |  | ITM2A | 8,46E-45 | -0,42 |
| GAPDH | 6,21E-175 | 0,59 |  | CTSC | 1,00E+00 | -0,42 |
| KRT8 | 9,73E-126 | 0,58 |  | RBP1 | 1,83E-33 | -0,42 |
| PALLD | 4,29E-209 | 0,58 |  | SPTBN1 | 7,61E-90 | -0,41 |
| KDELR2 | 6,73E-207 | 0,57 |  | SCN7A | 5,12E-43 | -0,40 |
| TPM1 | 2,32E-81 | 0,57 |  | NFIA | 6,02E-73 | -0,40 |
| C4orf48 | 1,58E-303 | 0,56 |  | ADAM28 | 4,86E-56 | -0,40 |
| ENO1 | 2,74E-108 | 0,56 |  | VAMP2 | 2,20E-82 | -0,40 |
| G0S2 | 2,84E-07 | 0,55 |  | LINC01082 | 2,67E-44 | -0,40 |
| SERPINF1 | 7,69E-72 | 0,55 |  | SNCG | 2,21E-26 | -0,40 |
| SDC1 | 0,00E+00 | 0,54 |  | WSB1 | 1,89E-69 | -0,40 |
| FBXO32 | 4,54E-172 | 0,54 |  | NFKBIA | 3,34E-55 | -0,40 |
| IGFBP5 | 7,57E-03 | 0,54 |  | MT-CO2 | 8,45E-107 | -0,40 |
| SSR3 | 5,67E-218 | 0,53 |  | ZBTB16 | 1,39E-96 | -0,39 |
| ARF4 | 1,61E-175 | 0,53 |  | TINAGL1 | 3,56E-33 | -0,39 |
| PODNL1 | 0,00E+00 | 0,52 |  | BMP4 | 9,58E-36 | -0,38 |
| IL6 | 2,38E-03 | 0,52 |  | BCAM | 1,77E-38 | -0,38 |
| ACTN1 | 7,06E-160 | 0,52 |  | C7 | 3,13E-02 | -0,38 |
| ACTB | 2,98E-96 | 0,52 |  | FRZB | 2,09E-04 | -0,38 |
| IL32 | 8,73E-114 | 0,52 |  | SORBS2 | 2,64E-17 | -0,38 |
| STEAP1 | 1,43E-244 | 0,52 |  | CIRBP | 2,45E-78 | -0,37 |
| NNMT | 2,24E-134 | 0,51 |  | HLA-C | 2,23E-62 | -0,37 |
| PLPP4 | 0,00E+00 | 0,51 |  | ADAMTS1 | 1,00E-48 | -0,37 |
| PLAUR | 4,77E-185 | 0,51 |  | MGP | 2,99E-04 | -0,37 |
| LMCD1 | 3,26E-181 | 0,51 |  | SERTAD1 | 1,23E-14 | -0,37 |
| COL6A2 | 3,26E-115 | 0,51 |  | FCGRT | 2,23E-46 | -0,36 |
| PPIB | 3,18E-209 | 0,51 |  | CST3 | 6,38E-54 | -0,36 |
| FNDC1 | 4,97E-292 | 0,50 |  | PLVAP | 4,01E-07 | -0,36 |
| EMILIN1 | 1,79E-154 | 0,49 |  | MGLL | 1,41E-39 | -0,36 |
| TMEM45A | 3,50E-235 | 0,49 |  | SEMA3B | 7,70E-17 | -0,36 |
| CKAP4 | 3,84E-218 | 0,49 |  | SON | 9,61E-66 | -0,35 |
| ANGPTL2 | 2,53E-142 | 0,48 |  | PNRC1 | 1,02E-62 | -0,35 |
| ADAMTS2 | 1,31E-278 | 0,47 |  | IL6ST | 1,38E-53 | -0,35 |
| IER3 | 6,89E-03 | 0,47 |  | LITAF | 1,38E-29 | -0,35 |
| IGHG4 | 3,17E-279 | 0,47 |  | SPRY1 | 2,71E-46 | -0,35 |
| CYR61 | 5,26E-12 | 0,47 |  | EFEMP1 | 1,00E+00 | -0,35 |
| RBP4 | 2,23E-183 | 0,47 |  | SFRP1 | 8,30E-37 | -0,34 |
| ITGBL1 | 1,70E-160 | 0,47 |  | FUS | 8,75E-53 | -0,34 |
| PDLIM2 | 6,69E-172 | 0,47 |  | CKB | 2,15E-52 | -0,34 |
| MMP7 | 5,48E-203 | 0,47 |  | HLA-DPA1 | 2,11E-38 | -0,34 |
| ERRFI1 | 1,31E-67 | 0,47 |  | TGFBR2 | 3,01E-32 | -0,34 |
| LTBP1 | 1,51E-196 | 0,46 |  | EMP2 | 9,75E-43 | -0,34 |
| CMTM3 | 6,43E-175 | 0,46 |  | TMEM176A | 2,30E-13 | -0,34 |
| LXN | 3,31E-157 | 0,46 |  | TACC1 | 2,40E-42 | -0,34 |
| SPATS2L | 1,14E-188 | 0,46 |  | NOVA1 | 4,18E-70 | -0,34 |
| COL7A1 | 1,58E-170 | 0,46 |  | MT1M | 1,00E+00 | -0,34 |
| RUNX1 | 1,31E-253 | 0,45 |  | GADD45G | 9,17E-20 | -0,33 |
| PYCR1 | 0,00E+00 | 0,45 |  | CALM2 | 1,62E-13 | -0,33 |
| SPOCK1 | 8,20E-285 | 0,45 |  | PEBP1 | 2,53E-34 | -0,33 |
| KDELR3 | 3,15E-207 | 0,45 |  | ZC3HAV1 | 2,62E-20 | -0,33 |
| CXCL2 | 6,13E-22 | 0,45 |  | DNAJA1 | 1,57E-23 | -0,32 |
| FKBP11 | 3,57E-196 | 0,45 |  | EPB41L2 | 2,34E-24 | -0,32 |
| HIF1A | 2,12E-142 | 0,45 |  | HLA-A | 2,73E-42 | -0,32 |
| SEC61G | 1,24E-148 | 0,45 |  | MT-ND4 | 2,47E-75 | -0,32 |
| ARL4C | 3,95E-287 | 0,44 |  | CCL11 | 1,00E+00 | -0,32 |
| CPXM1 | 4,83E-270 | 0,44 |  | INTS6 | 1,99E-21 | -0,32 |
| TMED3 | 1,35E-219 | 0,44 |  | PDLIM1 | 5,12E-48 | -0,31 |
| SLC12A8 | 2,23E-303 | 0,44 |  | MEF2C | 2,33E-33 | -0,31 |
| GPX8 | 2,84E-209 | 0,44 |  | SRGN | 1,00E+00 | -0,31 |
| BMP1 | 8,39E-271 | 0,44 |  | ARHGAP15 | 2,28E-46 | -0,31 |
| GEM | 4,03E-86 | 0,44 |  | CDKN1A | 3,20E-03 | -0,31 |
| SGK1 | 3,81E-29 | 0,43 |  | CADM3 | 1,26E-37 | -0,31 |
| DDIT4 | 1,00E+00 | 0,43 |  | CNBP | 2,23E-57 | -0,31 |
| LINC00152 | 3,66E-115 | 0,43 |  | MT-ND5 | 1,35E-60 | -0,31 |
| RP11-400N13.3 | 1,64E-302 | 0,43 |  | UBB | 4,21E-53 | -0,31 |
| MT1E | 7,13E-54 | 0,43 |  | STOM | 9,27E-49 | -0,31 |
| IGHG1 | 2,59E-157 | 0,42 |  | VAMP5 | 6,86E-44 | -0,31 |
| YIF1A | 7,23E-179 | 0,42 |  | STAT3 | 2,53E-19 | -0,30 |
| HDLBP | 7,17E-191 | 0,42 |  | IFI27 | 2,15E-15 | -0,30 |
| BASP1 | 1,24E-187 | 0,42 |  | EIF1 | 4,48E-60 | -0,30 |
| ACTA2 | 2,66E-29 | 0,42 |  | SEPW1 | 5,40E-39 | -0,30 |
| MRC2 | 6,16E-180 | 0,42 |  | PI16 | 6,12E-20 | -0,30 |
| SULF2 | 1,27E-239 | 0,42 |  | SOD1 | 1,47E-46 | -0,30 |
| CCL3 | 9,89E-92 | 0,42 |  | GLTSCR2 | 4,47E-50 | -0,29 |
| TMEM167A | 2,38E-200 | 0,42 |  | PNISR | 1,20E-33 | -0,29 |
| WNT2 | 1,10E-271 | 0,41 |  | SLC2A3 | 3,43E-09 | -0,29 |
| KRT19 | 4,85E-179 | 0,41 |  | CD81 | 4,33E-42 | -0,29 |
| PERP | 2,91E-159 | 0,41 |  | PPP1R10 | 1,77E-31 | -0,29 |
| MAGED1 | 7,52E-208 | 0,41 |  | HLA-DRB5 | 1,25E-22 | -0,29 |
| PPIC | 8,32E-109 | 0,41 |  | SRSF3 | 1,70E-40 | -0,29 |
| ITGA5 | 3,23E-96 | 0,41 |  | SLC9A3R2 | 1,30E-12 | -0,29 |
| PTK7 | 1,06E-219 | 0,41 |  | CD59 | 2,92E-20 | -0,29 |
| DPYSL3 | 4,26E-186 | 0,41 |  | SPP1 | 4,27E-21 | -0,29 |
| ALDOA | 4,01E-107 | 0,40 |  | N4BP2L2 | 4,99E-38 | -0,29 |
| TIMP3 | 2,46E-03 | 0,40 |  | HLA-DPB1 | 1,96E-20 | -0,28 |
| THBS1 | 2,39E-51 | 0,40 |  | CYGB | 5,33E-01 | -0,28 |
| TUBA1C | 1,12E-153 | 0,40 |  | FAM107A | 4,70E-38 | -0,28 |
| ITGAV | 2,62E-176 | 0,40 |  | F10 | 1,61E-40 | -0,28 |
| GPX7 | 2,93E-260 | 0,40 |  | ANAPC16 | 9,31E-40 | -0,28 |
| HILPDA | 4,50E-98 | 0,40 |  | F3 | 1,65E-04 | -0,28 |
| TUBB | 5,44E-88 | 0,39 |  | FABP1 | 1,55E-52 | -0,27 |
| EFEMP2 | 1,51E-115 | 0,39 |  | UBC | 1,34E-33 | -0,27 |
| IGFBP3 | 4,55E-32 | 0,39 |  | MYC | 2,09E-14 | -0,27 |
| CYP1B1 | 7,97E-94 | 0,39 |  | NFIB | 1,91E-33 | -0,27 |
| DIO2 | 1,95E-170 | 0,39 |  | SAMHD1 | 5,52E-30 | -0,27 |
| COL16A1 | 2,55E-155 | 0,39 |  | MTRNR2L8 | 9,78E-13 | -0,27 |
| CD82 | 3,26E-125 | 0,39 |  | HSP90AB1 | 6,18E-35 | -0,27 |
| ADAMTS12 | 1,27E-272 | 0,39 |  | SRSF7 | 3,59E-20 | -0,27 |
| AP2S1 | 6,35E-123 | 0,39 |  | CAV1 | 2,35E-01 | -0,27 |
| S100A11 | 4,50E-142 | 0,39 |  | SRP14 | 9,05E-45 | -0,27 |
| P3H4 | 5,60E-216 | 0,38 |  | PLPP1 | 4,85E-36 | -0,27 |
| PDGFC | 3,93E-257 | 0,38 |  | ITIH5 | 5,33E-45 | -0,27 |
| TAX1BP3 | 8,84E-122 | 0,38 |  | CRIP2 | 4,71E-24 | -0,27 |
| CD276 | 1,53E-207 | 0,38 |  | CSRP2 | 1,00E+00 | -0,27 |
| C5orf46 | 4,07E-240 | 0,38 |  | KLF2 | 7,94E-15 | -0,27 |
| SUGCT | 2,66E-205 | 0,38 |  | COX7A1 | 5,43E-32 | -0,27 |
| PLXDC2 | 3,66E-155 | 0,38 |  | WISP2 | 1,64E-07 | -0,27 |
| NREP | 1,16E-185 | 0,38 |  | MYLIP | 1,32E-32 | -0,26 |
| ACTG1 | 1,90E-116 | 0,37 |  | AP1S2 | 6,81E-02 | -0,26 |
| RPL28 | 8,55E-199 | 0,37 |  | PHGR1 | 3,17E-18 | -0,26 |
| S100A16 | 1,71E-99 | 0,37 |  | CLEC3B | 7,32E-44 | -0,26 |
| CAPZB | 7,81E-109 | 0,37 |  | ANGPTL1 | 1,60E-39 | -0,26 |
| COL15A1 | 6,06E-45 | 0,36 |  | NDUFA4L2 | 8,05E-32 | -0,26 |
| MYDGF | 1,47E-109 | 0,36 |  | NRN1 | 1,42E-34 | -0,26 |
| SURF4 | 1,25E-149 | 0,36 |  | SCPEP1 | 6,25E-17 | -0,26 |
| ABL2 | 7,85E-74 | 0,36 |  | LMNA | 1,56E-25 | -0,26 |
| PDLIM5 | 2,49E-117 | 0,36 |  | ADD3 | 2,50E-17 | -0,26 |
| TMSB10 | 6,65E-149 | 0,36 |  | MAFF | 4,40E-05 | -0,26 |
| PLOD1 | 1,18E-159 | 0,36 |  | RAMP2 | 1,53E-28 | -0,26 |
| ITGB1 | 6,79E-85 | 0,36 |  | YBX3 | 1,22E-24 | -0,26 |
| LRRC59 | 8,58E-157 | 0,36 |  | TMEM59 | 3,56E-33 | -0,26 |
| TIMP2 | 8,52E-93 | 0,36 |  | EBF1 | 6,78E-36 | -0,26 |
| CCL20 | 4,66E-71 | 0,36 |  | DST | 4,23E-10 | -0,25 |
| GJA1 | 1,19E-98 | 0,35 |  | EPHX1 | 1,10E-17 | -0,25 |
| NME4 | 9,42E-135 | 0,35 |  | TMEM100 | 1,75E-43 | -0,25 |
| P3H1 | 2,53E-183 | 0,35 |  | SKP1 | 4,69E-40 | -0,25 |
| CALB2 | 5,42E-152 | 0,35 |  | OGN | 1,00E+00 | -0,25 |
| MT1X | 2,99E-55 | 0,35 |  |  |  |  |
| MT1F | 5,99E-119 | 0,35 |  |  |  |  |
| TTC3 | 1,22E-103 | 0,35 |  |  |  |  |
| SEPT11 | 1,84E-100 | 0,35 |  |  |  |  |
| PRDX4 | 1,61E-103 | 0,35 |  |  |  |  |
| CD99 | 5,48E-91 | 0,34 |  |  |  |  |
| TPI1 | 1,18E-53 | 0,34 |  |  |  |  |
| MANF | 2,08E-111 | 0,34 |  |  |  |  |
| TGFB1 | 1,18E-125 | 0,34 |  |  |  |  |
| F2R | 1,46E-61 | 0,34 |  |  |  |  |
| KRT18 | 1,13E-85 | 0,34 |  |  |  |  |
| ARPC2 | 7,05E-113 | 0,34 |  |  |  |  |
| FSTL1 | 9,07E-108 | 0,34 |  |  |  |  |
| TMEM158 | 2,01E-214 | 0,34 |  |  |  |  |
| AC090498.1 | 1,15E-106 | 0,33 |  |  |  |  |
| HSPA5 | 3,30E-25 | 0,33 |  |  |  |  |
| PGAM1 | 7,78E-75 | 0,33 |  |  |  |  |
| ANKRD28 | 1,85E-88 | 0,33 |  |  |  |  |
| GLIPR1 | 5,47E-149 | 0,33 |  |  |  |  |
| RCN1 | 1,21E-93 | 0,33 |  |  |  |  |
| TFF1 | 4,54E-120 | 0,33 |  |  |  |  |
| UNC5B | 1,03E-156 | 0,33 |  |  |  |  |
| GOLM1 | 2,63E-182 | 0,33 |  |  |  |  |
| SEZ6L2 | 1,22E-282 | 0,33 |  |  |  |  |
| PDLIM4 | 1,69E-101 | 0,33 |  |  |  |  |
| SH3PXD2A | 5,03E-118 | 0,33 |  |  |  |  |
| NTM | 3,71E-152 | 0,32 |  |  |  |  |
| OLFML2B | 1,02E-136 | 0,32 |  |  |  |  |
| VMP1 | 4,85E-44 | 0,32 |  |  |  |  |
| ANKH | 3,75E-169 | 0,32 |  |  |  |  |
| ENC1 | 7,53E-146 | 0,32 |  |  |  |  |
| TWIST1 | 6,67E-227 | 0,32 |  |  |  |  |
| ENAH | 1,37E-91 | 0,32 |  |  |  |  |
| LY6E | 1,38E-58 | 0,32 |  |  |  |  |
| LDHA | 6,38E-46 | 0,32 |  |  |  |  |
| LOXL1 | 1,83E-123 | 0,32 |  |  |  |  |
| LMO7 | 6,72E-227 | 0,32 |  |  |  |  |
| ITGB5 | 1,22E-124 | 0,32 |  |  |  |  |
| SLC39A14 | 7,04E-164 | 0,32 |  |  |  |  |
| RPLP1 | 3,41E-174 | 0,31 |  |  |  |  |
| GLT8D2 | 1,19E-110 | 0,31 |  |  |  |  |
| PLIN3 | 6,87E-130 | 0,31 |  |  |  |  |
| C1QTNF6 | 8,40E-219 | 0,31 |  |  |  |  |
| RPL22L1 | 4,76E-79 | 0,31 |  |  |  |  |
| ATP6V0B | 3,85E-111 | 0,31 |  |  |  |  |
| WNT5A | 1,47E-115 | 0,31 |  |  |  |  |
| HCFC1R1 | 2,34E-65 | 0,31 |  |  |  |  |
| FTL | 1,27E-58 | 0,31 |  |  |  |  |
| PFN1 | 1,82E-83 | 0,31 |  |  |  |  |
| CAPG | 6,74E-164 | 0,31 |  |  |  |  |
| PTTG1IP | 1,71E-106 | 0,31 |  |  |  |  |
| CREB3L1 | 2,18E-146 | 0,31 |  |  |  |  |
| AGR2 | 1,23E-117 | 0,30 |  |  |  |  |
| EPSTI1 | 1,54E-124 | 0,30 |  |  |  |  |
| SELM | 6,88E-63 | 0,30 |  |  |  |  |
| OST4 | 1,55E-93 | 0,30 |  |  |  |  |
| TGFB3 | 5,75E-126 | 0,30 |  |  |  |  |
| B4GALT1 | 2,01E-67 | 0,30 |  |  |  |  |
| SEC13 | 1,14E-124 | 0,30 |  |  |  |  |
| FMOD | 1,90E-120 | 0,30 |  |  |  |  |
| MYH9 | 2,41E-62 | 0,30 |  |  |  |  |
| BCAT1 | 9,93E-178 | 0,30 |  |  |  |  |
| TMEM208 | 1,29E-117 | 0,30 |  |  |  |  |
| EVA1A | 9,11E-169 | 0,30 |  |  |  |  |
| NME1 | 6,12E-101 | 0,30 |  |  |  |  |
| IGFBP4 | 1,77E-40 | 0,30 |  |  |  |  |
| SPINK4 | 2,06E-75 | 0,30 |  |  |  |  |
| MDFI | 4,73E-208 | 0,30 |  |  |  |  |
| TNFAIP3 | 1,55E-24 | 0,30 |  |  |  |  |
| SH3BGRL3 | 1,15E-75 | 0,30 |  |  |  |  |
| MMP23B | 1,01E-33 | 0,30 |  |  |  |  |
| PRAF2 | 2,87E-120 | 0,29 |  |  |  |  |
| CNIH1 | 4,80E-108 | 0,29 |  |  |  |  |
| RUNX2 | 8,19E-218 | 0,29 |  |  |  |  |
| SLC20A1 | 1,41E-101 | 0,29 |  |  |  |  |
| CHN1 | 1,04E-81 | 0,29 |  |  |  |  |
| PXDN | 4,50E-93 | 0,29 |  |  |  |  |
| BICC1 | 4,99E-125 | 0,29 |  |  |  |  |
| TDO2 | 6,52E-57 | 0,29 |  |  |  |  |
| ITGA11 | 3,52E-124 | 0,29 |  |  |  |  |
| FIBIN | 2,37E-81 | 0,29 |  |  |  |  |
| RAI14 | 6,06E-136 | 0,29 |  |  |  |  |
| REG4 | 5,57E-152 | 0,29 |  |  |  |  |
| FKBP7 | 7,53E-115 | 0,29 |  |  |  |  |
| APOD | 2,19E-01 | 0,29 |  |  |  |  |
| VGLL4 | 8,65E-102 | 0,28 |  |  |  |  |
| COPZ2 | 9,27E-125 | 0,28 |  |  |  |  |
| LMAN1 | 1,09E-96 | 0,28 |  |  |  |  |
| SEC61A1 | 8,96E-128 | 0,28 |  |  |  |  |
| SLC6A6 | 1,65E-124 | 0,28 |  |  |  |  |
| COTL1 | 5,36E-71 | 0,28 |  |  |  |  |
| INAFM1 | 1,50E-96 | 0,28 |  |  |  |  |
| CA12 | 3,11E-95 | 0,28 |  |  |  |  |
| SPON1 | 3,28E-110 | 0,28 |  |  |  |  |
| PDGFRL | 6,92E-99 | 0,28 |  |  |  |  |
| TMEM98 | 1,19E-102 | 0,28 |  |  |  |  |
| GSTO1 | 9,26E-41 | 0,28 |  |  |  |  |
| CFI | 8,89E-121 | 0,28 |  |  |  |  |
| ALDH1A3 | 1,44E-42 | 0,28 |  |  |  |  |
| SOX4 | 5,79E-57 | 0,28 |  |  |  |  |
| IGLC7 | 1,41E-48 | 0,28 |  |  |  |  |
| FSCN1 | 5,67E-95 | 0,28 |  |  |  |  |
| DES | 9,53E-18 | 0,28 |  |  |  |  |
| MPZL1 | 3,12E-122 | 0,28 |  |  |  |  |
| ID3 | 7,15E-04 | 0,28 |  |  |  |  |
| IL7R | 7,06E-85 | 0,28 |  |  |  |  |
| LTBP2 | 5,36E-112 | 0,28 |  |  |  |  |
| KIAA1217 | 1,10E-154 | 0,27 |  |  |  |  |
| LINC00657 | 6,84E-91 | 0,27 |  |  |  |  |
| PHLDA3 | 8,06E-74 | 0,27 |  |  |  |  |
| NINJ1 | 1,21E-72 | 0,27 |  |  |  |  |
| WISP1 | 1,63E-256 | 0,27 |  |  |  |  |
| MARCKSL1 | 4,56E-68 | 0,27 |  |  |  |  |
| C1orf122 | 2,58E-95 | 0,27 |  |  |  |  |
| EGFL6 | 1,63E-79 | 0,27 |  |  |  |  |
| TYMP | 1,54E-42 | 0,27 |  |  |  |  |
| SRPX2 | 3,34E-135 | 0,27 |  |  |  |  |
| PRSS23 | 1,47E-24 | 0,27 |  |  |  |  |
| PLIN2 | 1,91E-41 | 0,27 |  |  |  |  |
| MYH10 | 2,00E-131 | 0,27 |  |  |  |  |
| ZFAND2A | 9,24E-25 | 0,27 |  |  |  |  |
| BHLHE41 | 3,88E-187 | 0,27 |  |  |  |  |
| FGF7 | 4,44E-38 | 0,27 |  |  |  |  |
| ACTR3 | 5,83E-81 | 0,27 |  |  |  |  |
| PGK1 | 2,00E-24 | 0,27 |  |  |  |  |
| MICAL2 | 1,05E-187 | 0,27 |  |  |  |  |
| EIF4E2 | 4,67E-115 | 0,27 |  |  |  |  |
| FHL2 | 2,18E-83 | 0,27 |  |  |  |  |
| RGS3 | 6,55E-72 | 0,27 |  |  |  |  |
| RIN2 | 9,95E-81 | 0,27 |  |  |  |  |
| UQCC2 | 4,32E-103 | 0,27 |  |  |  |  |
| KDELR1 | 2,84E-82 | 0,27 |  |  |  |  |
| TPBG | 6,92E-110 | 0,27 |  |  |  |  |
| MEST | 2,43E-82 | 0,27 |  |  |  |  |
| P4HA1 | 1,42E-100 | 0,27 |  |  |  |  |
| CCDC34 | 4,62E-119 | 0,26 |  |  |  |  |
| KLF6 | 1,75E-13 | 0,26 |  |  |  |  |
| MATN3 | 1,72E-111 | 0,26 |  |  |  |  |
| TMED9 | 2,38E-63 | 0,26 |  |  |  |  |
| ZYX | 1,41E-94 | 0,26 |  |  |  |  |
| CNN1 | 3,87E-57 | 0,26 |  |  |  |  |
| NRP2 | 3,85E-108 | 0,26 |  |  |  |  |
| CFL1 | 1,11E-60 | 0,26 |  |  |  |  |
| FAM3C | 3,74E-81 | 0,26 |  |  |  |  |
| SVIL | 1,14E-88 | 0,26 |  |  |  |  |
| DCBLD1 | 3,14E-131 | 0,26 |  |  |  |  |
| FAM20C | 4,82E-109 | 0,26 |  |  |  |  |
| IKBIP | 7,86E-104 | 0,26 |  |  |  |  |
| SLC44A1 | 6,39E-84 | 0,26 |  |  |  |  |
| PDGFRB | 1,91E-77 | 0,26 |  |  |  |  |
| SEC31A | 4,13E-98 | 0,26 |  |  |  |  |
| WTAP | 1,52E-38 | 0,26 |  |  |  |  |
| MIR4435-2HG | 6,28E-73 | 0,26 |  |  |  |  |
| PRELID1 | 2,35E-66 | 0,26 |  |  |  |  |
| SEC24D | 2,28E-125 | 0,26 |  |  |  |  |
| STEAP2 | 4,04E-116 | 0,26 |  |  |  |  |
| SAR1A | 1,49E-79 | 0,26 |  |  |  |  |
| WIPI1 | 1,78E-118 | 0,25 |  |  |  |  |
| SERPINE2 | 8,44E-04 | 0,25 |  |  |  |  |
| GGT5 | 1,90E-62 | 0,25 |  |  |  |  |
| KIAA0930 | 3,04E-124 | 0,25 |  |  |  |  |
| ANGPTL4 | 7,47E-06 | 0,25 |  |  |  |  |
| CDK4 | 1,12E-87 | 0,25 |  |  |  |  |

**Table S2 – Differential gene expression analysis of FAP-positive and FAP-negative stromal cells.** Single cell RNA expression data from colon tumors (cohorts GSE144735 and GSE132465) were used for differential gene expression analysis between FAP–expressing and non-expressing stromal cells defined by non-zero or zero-value FAP counts respectively, using the FindMarkers function with default parameters (wilcox test, logfc.threshold = 0.25).
