# Supplementary material for: Fibroblast activation protein identifies Consensus Molecular Subtype 4 in colorectal cancer and allows its detection by 68Ga-FAPI-PET imaging
Source: Br J Cancer. 2022 Mar 16;127(1):145–55. doi: 10.1038/s41416-022-01748-z (PMC9276750; doi:10.1038/s41416-022-01748-z)
Supplement: Supplementary file 4 — Table S3 [file 41416_2022_1748_MOESM4_ESM.docx]

| **Genes sign. UP in FAP+ tumor cells** | | |  | **Genes sign. DOWN in FAP+ tumor cells** | | |
| --- | --- | --- | --- | --- | --- | --- |
| **hugo** | **corrected P** | **Log2 fold-**  **change** | | **hugo** | **corrected P** | **Log2 fold-change** |
| FAP | 2,31E-77 | 1,16 |  | PLCG2 | 1,00E+00 | -2,18 |
| CLCA1 | 6,13E+08 | 1,09 |  | MALAT1 | 1,46E+03 | -1,60 |
| IGFBP2 | 1,82E-02 | 1,05 |  | C15orf48 | 1,00E+00 | -1,40 |
| PRSS2 | 2,17E-02 | 1,03 |  | SOX4 | 1,64E-04 | -1,36 |
| ITLN1 | 9,92E+00 | 0,96 |  | LCN2 | 1,00E+00 | -1,13 |
| HEPACAM2 | 7,06E-12 | 0,88 |  | ANXA1 | 1,14E-03 | -1,13 |
| FRZB | 9,89E-10 | 0,85 |  | PSCA | 2,54E-02 | -1,08 |
| HMGN2 | 5,99E+01 | 0,81 |  | TXNIP | 1,10E+09 | -1,04 |
| AQP3 | 8,34E-08 | 0,81 |  | CXCL1 | 1,00E+00 | -1,03 |
| KLK12 | 6,55E-10 | 0,81 |  | SLPI | 1,00E+00 | -1,00 |
| SPINK4 | 1,23E-01 | 0,77 |  | SLC6A8 | 1,00E+00 | -0,99 |
| RPS2 | 1,31E-14 | 0,76 |  | C19orf33 | 7,69E-03 | -0,98 |
| AQP5 | 2,25E+01 | 0,74 |  | S100A2 | 1,00E+00 | -0,96 |
| SERPINA1 | 1,05E+05 | 0,73 |  | KLK10 | 6,74E-01 | -0,95 |
| PLA2G2A | 1,00E+00 | 0,73 |  | PRSS3 | 1,99E-04 | -0,95 |
| YBX1 | 9,56E-09 | 0,72 |  | S100A4 | 1,82E-03 | -0,94 |
| HES6 | 4,27E-03 | 0,71 |  | CDKN2B | 3,86E+08 | -0,93 |
| PHLDA1 | 4,84E+03 | 0,70 |  | TSPAN8 | 1,00E+00 | -0,91 |
| REG4 | 2,11E+06 | 0,69 |  | TSPAN1 | 1,28E-04 | -0,91 |
| RPL5 | 7,88E-07 | 0,68 |  | SAT1 | 7,32E+05 | -0,90 |
| GADD45A | 1,59E-02 | 0,67 |  | CXCL2 | 1,00E+00 | -0,88 |
| ST6GALNAC1 | 2,14E-03 | 0,67 |  | MMP7 | 1,00E+00 | -0,87 |
| LRRC26 | 6,36E-04 | 0,67 |  | TFF2 | 1,00E+00 | -0,86 |
| RPL3 | 2,61E-10 | 0,67 |  | CLIC3 | 4,05E-02 | -0,86 |
| ODC1 | 6,00E-01 | 0,66 |  | EPS8L1 | 1,00E+00 | -0,86 |
| GPX2 | 2,58E-01 | 0,64 |  | GPRC5A | 1,63E-03 | -0,85 |
| GTF3A | 1,18E-04 | 0,64 |  | LAMB3 | 1,34E-03 | -0,84 |
| RPLP0 | 6,47E-18 | 0,64 |  | ERO1A | 1,00E+00 | -0,82 |
| RPSA | 1,55E-03 | 0,63 |  | CEACAM1 | 1,00E+00 | -0,82 |
| MPC2 | 4,56E-04 | 0,61 |  | NEAT1 | 1,33E-02 | -0,82 |
| KIAA1324 | 2,33E-06 | 0,60 |  | IL32 | 1,00E+00 | -0,81 |
| SELM | 1,05E+02 | 0,59 |  | CXCL3 | 1,00E+00 | -0,81 |
| SEC11C | 3,11E-01 | 0,58 |  | CDKN2A | 3,18E-01 | -0,81 |
| HNRNPA1 | 4,12E+04 | 0,58 |  | HSPA1A | 1,00E+00 | -0,80 |
| SLC25A6 | 2,20E+00 | 0,57 |  | ANKRD36C | 1,00E+00 | -0,79 |
| RPL4 | 1,47E-03 | 0,57 |  | CD24 | 1,00E+00 | -0,79 |
| SPINK1 | 5,40E+06 | 0,57 |  | RHOC | 2,71E-03 | -0,78 |
| TMED2 | 1,86E+01 | 0,57 |  | MT1X | 1,00E+00 | -0,76 |
| TSPAN13 | 2,71E+01 | 0,57 |  | CSTB | 2,97E-01 | -0,74 |
| CYCS | 2,69E+00 | 0,56 |  | PLAT | 1,00E+00 | -0,74 |
| LINC00261 | 1,72E-03 | 0,56 |  | NTHL1 | 1,00E+00 | -0,73 |
| HPCAL1 | 6,07E-02 | 0,56 |  | C4orf48 | 5,34E-02 | -0,72 |
| RPS6 | 4,31E-04 | 0,56 |  | JUN | 1,00E+00 | -0,72 |
| RPS4X | 3,00E-03 | 0,56 |  | AGRN | 1,00E+00 | -0,72 |
| RPL7 | 6,61E-02 | 0,55 |  | ASS1 | 1,00E+00 | -0,72 |
| NACA | 1,86E+00 | 0,54 |  | ELF3 | 6,08E-04 | -0,71 |
| MRPS35 | 6,84E-02 | 0,53 |  | CTSD | 1,57E-02 | -0,71 |
| MDH2 | 1,73E-01 | 0,53 |  | ITGB1 | 4,77E-02 | -0,70 |
| PCSK1 | 3,74E+02 | 0,53 |  | HSPA1B | 1,00E+00 | -0,70 |
| IFI6 | 5,48E+03 | 0,53 |  | C8orf4 | 1,00E+00 | -0,68 |
| STOML2 | 9,67E-02 | 0,53 |  | PLEC | 7,35E-03 | -0,68 |
| RPS3A | 8,65E-04 | 0,52 |  | NDRG1 | 1,00E+00 | -0,68 |
| RPL21 | 1,30E-03 | 0,52 |  | SDCBP2 | 1,00E+00 | -0,67 |
| FABP5 | 1,44E+02 | 0,51 |  | HSPG2 | 8,36E-02 | -0,67 |
| ATP5G1 | 2,92E+01 | 0,50 |  | TP53INP2 | 1,00E+00 | -0,67 |
| TMEM98 | 6,00E-05 | 0,49 |  | ACHE | 3,98E-02 | -0,67 |
| RPS8 | 9,38E-05 | 0,49 |  | MUC1 | 1,00E+00 | -0,66 |
| RPLP1 | 6,52E-05 | 0,49 |  | RND3 | 1,00E+00 | -0,66 |
| RPN2 | 4,43E+02 | 0,49 |  | CD59 | 1,00E+00 | -0,66 |
| RPL10A | 5,63E+01 | 0,49 |  | TMBIM1 | 5,23E-01 | -0,66 |
| PPP1R1B | 5,02E-01 | 0,48 |  | S100A6 | 3,65E+05 | -0,64 |
| JTB | 7,09E+03 | 0,48 |  | PRSS8 | 1,00E+00 | -0,64 |
| HMGN3 | 2,47E+00 | 0,48 |  | PRSS22 | 1,00E+00 | -0,63 |
| LAMA1 | 7,29E-03 | 0,48 |  | CTSE | 7,68E-01 | -0,62 |
| HINT1 | 6,95E+04 | 0,48 |  | NFKBIZ | 1,00E+00 | -0,62 |
| STMN1 | 2,58E-02 | 0,48 |  | SEMA3B | 4,39E-04 | -0,62 |
| TSTA3 | 7,89E+02 | 0,47 |  | MYH14 | 2,78E-01 | -0,62 |
| PRDX4 | 1,72E+01 | 0,47 |  | EFNA1 | 1,00E+00 | -0,61 |
| KDELR2 | 5,38E+05 | 0,47 |  | ECM1 | 1,00E+00 | -0,60 |
| TMEM106C | 4,31E+04 | 0,47 |  | PDZK1IP1 | 1,00E+00 | -0,60 |
| SERBP1 | 1,07E+03 | 0,47 |  | ABHD11-AS1 | 1,00E+00 | -0,60 |
| NME1 | 9,05E+00 | 0,47 |  | MT-ND4L | 1,00E+00 | -0,60 |
| RAP1GAP | 3,06E+00 | 0,47 |  | KRT23 | 1,00E+00 | -0,60 |
| RPS24 | 5,11E-02 | 0,47 |  | HES1 | 1,00E+00 | -0,59 |
| RPL6 | 1,49E+04 | 0,46 |  | APOL1 | 4,97E-02 | -0,59 |
| RPS10 | 4,26E+00 | 0,46 |  | CEACAM6 | 1,64E-02 | -0,59 |
| SLC1A5 | 1,14E+05 | 0,46 |  | PLXNB2 | 1,00E+00 | -0,59 |
| AGR2 | 3,33E-04 | 0,46 |  | HSPB1 | 5,87E-01 | -0,58 |
| RPL12 | 1,31E+01 | 0,46 |  | HSPA6 | 1,00E+00 | -0,58 |
| LRRC59 | 4,49E+03 | 0,46 |  | RRBP1 | 1,00E+00 | -0,58 |
| HMGB1 | 2,21E-04 | 0,46 |  | DUSP5 | 1,00E+00 | -0,58 |
| BTF3 | 1,41E+02 | 0,46 |  | G0S2 | 1,00E+00 | -0,57 |
| RPL23A | 3,92E-03 | 0,46 |  | DHRS9 | 4,49E-01 | -0,57 |
| RPS20 | 1,43E+02 | 0,45 |  | SPINT1 | 8,66E-01 | -0,56 |
| SSR2 | 7,90E+05 | 0,45 |  | ANXA3 | 1,00E+00 | -0,56 |
| SRP9 | 1,73E+03 | 0,45 |  | ABL2 | 1,00E+00 | -0,56 |
| LYZ | 4,69E-04 | 0,45 |  | FLNB | 1,00E+00 | -0,56 |
| MCL1 | 4,47E+05 | 0,45 |  | IER5L | 1,00E+00 | -0,56 |
| ETS2 | 8,82E+06 | 0,45 |  | TIMP2 | 1,71E-01 | -0,55 |
| DNPEP | 2,27E+01 | 0,44 |  | MT1G | 1,00E+00 | -0,55 |
| SNRPD1 | 7,41E+04 | 0,44 |  | ID1 | 1,00E+00 | -0,54 |
| RPL27 | 1,91E-01 | 0,44 |  | KRT6B | 1,00E+00 | -0,54 |
| RPL15 | 2,04E+03 | 0,44 |  | WDR60 | 1,00E+00 | -0,53 |
| RPS13 | 3,80E+02 | 0,44 |  | TNNC2 | 1,00E+00 | -0,53 |
| CREB3L1 | 1,18E+03 | 0,44 |  | CEBPB | 1,00E+00 | -0,53 |
| RPS7 | 7,54E+00 | 0,43 |  | LMO7 | 1,00E+00 | -0,53 |
| GMDS | 1,17E+03 | 0,43 |  | CCND1 | 1,00E+00 | -0,53 |
| RPS5 | 4,21E+01 | 0,43 |  | RNASET2 | 1,00E+00 | -0,53 |
| EEF1B2 | 2,43E+06 | 0,43 |  | CKB | 1,00E+00 | -0,52 |
| B3GNT7 | 6,63E-01 | 0,43 |  | PTGS2 | 1,00E+00 | -0,51 |
| RPL30 | 9,22E-01 | 0,43 |  | GPX4 | 4,71E-01 | -0,51 |
| HNRNPA3 | 1,24E+04 | 0,43 |  | MYO6 | 1,00E+00 | -0,51 |
| ATP5G2 | 4,71E+07 | 0,43 |  | VEGFA | 1,00E+00 | -0,51 |
| ICA1 | 3,93E+00 | 0,43 |  | APLP2 | 1,00E+00 | -0,51 |
| RAN | 1,44E+05 | 0,43 |  | OVOS2 | 1,00E+00 | -0,50 |
| RPL14 | 4,54E+01 | 0,42 |  | TIMP1 | 1,00E+00 | -0,50 |
| REXO2 | 3,88E+02 | 0,42 |  | TLE4 | 1,00E+00 | -0,50 |
| RPL19 | 3,10E-02 | 0,42 |  | CEACAM5 | 1,00E+00 | -0,50 |
| CDK4 | 3,01E+00 | 0,42 |  | CD55 | 1,00E+00 | -0,50 |
| CHCHD2 | 4,05E+03 | 0,42 |  | CST6 | 1,00E+00 | -0,49 |
| SSR3 | 5,72E+06 | 0,42 |  | MSLN | 1,00E+00 | -0,49 |
| TCEA3 | 1,54E+02 | 0,41 |  | FAM3C | 1,00E+00 | -0,48 |
| PARK7 | 1,50E+06 | 0,41 |  | DUOXA2 | 1,00E+00 | -0,48 |
| TPD52 | 3,66E+04 | 0,41 |  | FOS | 1,00E+00 | -0,48 |
| DUSP23 | 1,77E+04 | 0,41 |  | TCF25 | 1,00E+00 | -0,48 |
| CMIP | 1,43E+03 | 0,41 |  | CCNL1 | 1,00E+00 | -0,48 |
| DDOST | 7,79E+04 | 0,41 |  | S100P | 1,00E+00 | -0,48 |
| PEBP1 | 4,79E+04 | 0,41 |  | EPS8L2 | 1,00E+00 | -0,47 |
| SNRPF | 2,25E+06 | 0,41 |  | ADAM9 | 1,00E+00 | -0,47 |
| ZCRB1 | 5,94E+03 | 0,41 |  | CXCL5 | 1,00E+00 | -0,47 |
| C1QBP | 1,50E+02 | 0,41 |  | KRT7 | 1,00E+00 | -0,47 |
| SERP1 | 3,93E+06 | 0,40 |  | C6orf15 | 1,00E+00 | -0,46 |
| RP11-234B24.2 | 2,90E-01 | 0,40 |  | S100A10 | 1,00E+00 | -0,46 |
| IFITM3 | 1,62E+05 | 0,40 |  | MUC12 | 1,00E+00 | -0,46 |
| PPP1CC | 3,68E+05 | 0,40 |  | CIB1 | 1,00E+00 | -0,46 |
| RPL8 | 3,03E+00 | 0,40 |  | BCL2L1 | 1,00E+00 | -0,46 |
| RPL9 | 8,52E+02 | 0,40 |  | PKM | 1,00E+00 | -0,46 |
| MINOS1 | 1,35E+05 | 0,40 |  | MALL | 1,00E+00 | -0,46 |
| UAP1 | 4,73E+03 | 0,40 |  | ITGB8 | 1,00E+00 | -0,46 |
| CCND2 | 2,61E+02 | 0,40 |  | ALDOA | 1,00E+00 | -0,46 |
| RPS3 | 5,11E+04 | 0,40 |  | JUND | 1,00E+00 | -0,45 |
| RPL17 | 7,74E+05 | 0,40 |  | PTTG1IP | 1,00E+00 | -0,45 |
| NDUFB9 | 2,61E+06 | 0,39 |  | RP1-313I6.12 | 1,00E+00 | -0,45 |
| TGFBI | 1,68E+04 | 0,39 |  | SULT2B1 | 1,00E+00 | -0,45 |
| TESC | 4,86E+06 | 0,39 |  | SLC16A3 | 1,00E+00 | -0,45 |
| RPL11 | 1,08E+03 | 0,39 |  | UPP1 | 1,00E+00 | -0,45 |
| TMEM183A | 2,82E+04 | 0,39 |  | FLNA | 1,00E+00 | -0,45 |
| MRPL24 | 1,61E+03 | 0,39 |  | QSOX1 | 5,68E-02 | -0,44 |
| NDUFA4 | 3,18E+07 | 0,39 |  | RP1-122P22.4 | 1,00E+00 | -0,44 |
| INPP1 | 6,02E+03 | 0,39 |  | MAP1LC3A | 1,00E+00 | -0,44 |
| SRM | 4,78E+02 | 0,38 |  | SUN1 | 1,00E+00 | -0,44 |
| RPS23 | 9,24E+03 | 0,38 |  | DYNLRB1 | 1,00E+00 | -0,44 |
| PTTG1 | 3,22E-04 | 0,38 |  | TRIM31 | 1,00E+00 | -0,44 |
| RPL22 | 9,16E+05 | 0,38 |  | EPS8 | 1,00E+00 | -0,44 |
| RPS17 | 2,25E+03 | 0,38 |  | RHBDF1 | 1,00E+00 | -0,44 |
| SNRPB | 1,66E+07 | 0,38 |  | ITGB4 | 1,00E+00 | -0,43 |
| MEST | 4,69E-01 | 0,38 |  | RHOF | 1,00E+00 | -0,43 |
| MRPL3 | 6,31E+04 | 0,38 |  | RAMP1 | 1,00E+00 | -0,43 |
| AGR3 | 1,45E+07 | 0,38 |  | SPTAN1 | 1,00E+00 | -0,43 |
| RPS12 | 6,08E-01 | 0,38 |  | DUSP1 | 1,00E+00 | -0,43 |
| ATP5B | 8,61E+06 | 0,38 |  | HEXIM1 | 1,00E+00 | -0,43 |
| SRSF9 | 5,40E+06 | 0,38 |  | EMP3 | 1,00E+00 | -0,43 |
| EIF3E | 3,78E+06 | 0,37 |  | HIST1H2BG | 1,00E+00 | -0,42 |
| H2AFZ | 2,42E-01 | 0,37 |  | TAGLN | 1,00E+00 | -0,42 |
| RPL32 | 7,52E+01 | 0,37 |  | MAL2 | 1,00E+00 | -0,42 |
| EIF4EBP1 | 2,70E+06 | 0,37 |  | TMSB4X | 1,00E+00 | -0,42 |
| ARL5A | 3,45E+03 | 0,37 |  | RP11-467L13.7 | 1,00E+00 | -0,42 |
| TSTD1 | 1,38E+09 | 0,37 |  | LPIN2 | 1,00E+00 | -0,42 |
| NCOA7 | 6,32E+06 | 0,37 |  | PI3 | 1,00E+00 | -0,42 |
| NDUFC2 | 7,73E+05 | 0,37 |  | TFF1 | 1,00E+00 | -0,41 |
| HBEGF | 8,29E+07 | 0,37 |  | KRT17 | 1,00E+00 | -0,41 |
| C1orf43 | 2,05E+04 | 0,37 |  | SQSTM1 | 1,00E+00 | -0,41 |
| MZT2B | 2,23E+07 | 0,37 |  | S100A11 | 1,00E+00 | -0,41 |
| RPL18A | 2,60E+00 | 0,37 |  | MYO7B | 1,00E+00 | -0,41 |
| EI24 | 5,87E+03 | 0,37 |  | MTRNR2L12 | 1,00E+00 | -0,41 |
| MRPL20 | 1,68E+07 | 0,37 |  | MUC3A | 1,00E+00 | -0,41 |
| PARP1 | 2,00E+02 | 0,36 |  | PGK1 | 1,00E+00 | -0,41 |
| HNRNPA2B1 | 8,63E-04 | 0,36 |  | VWA1 | 1,00E+00 | -0,41 |
| RBP4 | 1,95E+06 | 0,36 |  | GRN | 1,00E+00 | -0,41 |
| RSL1D1 | 9,11E+05 | 0,36 |  | TSPYL2 | 1,00E+00 | -0,41 |
| ARF5 | 7,02E+07 | 0,36 |  | GOLM1 | 5,90E-01 | -0,41 |
| RPS27A | 3,67E+04 | 0,36 |  | TAX1BP1 | 1,00E+00 | -0,41 |
| FABP2 | 2,49E+03 | 0,36 |  | MTRNR2L8 | 1,00E+00 | -0,41 |
| RPL7A | 1,42E+05 | 0,36 |  | ISG20 | 1,00E+00 | -0,40 |
| RBM3 | 3,18E+05 | 0,36 |  | H2AFJ | 1,00E+00 | -0,40 |
| HIST1H4C | 7,86E-02 | 0,36 |  | C4BPB | 1,00E+00 | -0,40 |
| LAPTM4B | 2,02E+07 | 0,36 |  | PSAP | 1,00E+00 | -0,40 |
| ZNF511 | 7,77E+02 | 0,36 |  | ARHGEF16 | 1,00E+00 | -0,40 |
| RPS9 | 2,72E+05 | 0,35 |  | LINC00152 | 1,00E+00 | -0,40 |
| UQCRH | 3,48E+08 | 0,35 |  | COL6A1 | 1,00E+00 | -0,40 |
| GDF15 | 4,81E-01 | 0,35 |  | ISG15 | 1,00E+00 | -0,39 |
| EBNA1BP2 | 7,79E+04 | 0,35 |  | RASSF7 | 1,00E+00 | -0,39 |
| CNPY2 | 2,56E+06 | 0,35 |  | EMP1 | 1,00E+00 | -0,39 |
| FRMD4B | 1,29E+05 | 0,35 |  | GADD45B | 1,00E+00 | -0,39 |
| NDUFB1 | 2,10E+05 | 0,35 |  | JUP | 1,00E+00 | -0,39 |
| IGFBP5 | 5,37E-02 | 0,35 |  | MYL12B | 1,00E+00 | -0,39 |
| SET | 2,86E+09 | 0,35 |  | SERPINE2 | 1,00E+00 | -0,39 |
| ATP5G3 | 1,29E+08 | 0,35 |  | KLF2 | 1,00E+00 | -0,38 |
| ATP1A1 | 8,27E+07 | 0,35 |  | LAMC2 | 1,00E+00 | -0,38 |
| SEC61B | 3,60E-04 | 0,35 |  | TRIM29 | 1,00E+00 | -0,38 |
| RPL26 | 1,51E+07 | 0,35 |  | COX6B2 | 1,00E+00 | -0,38 |
| PDIA3 | 3,61E+09 | 0,35 |  | MET | 1,00E+00 | -0,38 |
| RPS28 | 6,04E+01 | 0,34 |  | DDIT4 | 1,00E+00 | -0,38 |
| RAD23A | 2,35E+07 | 0,34 |  | MVP | 1,00E+00 | -0,38 |
| XBP1 | 3,89E+07 | 0,34 |  | MIR4435-2HG | 1,00E+00 | -0,38 |
| HNRNPUL1 | 5,91E+07 | 0,34 |  | CAPG | 1,00E+00 | -0,38 |
| DMBT1 | 1,20E+08 | 0,34 |  | TRPM4 | 1,00E+00 | -0,38 |
| SOD1 | 1,04E+09 | 0,34 |  | TM4SF1 | 1,00E+00 | -0,38 |
| OSTC | 3,03E-04 | 0,34 |  | TAX1BP3 | 1,00E+00 | -0,37 |
| PHB | 5,62E+05 | 0,34 |  | GTF2I | 1,00E+00 | -0,37 |
| WBSCR22 | 1,36E+06 | 0,34 |  | LINC01133 | 1,00E+00 | -0,37 |
| HMGN1 | 1,72E-03 | 0,34 |  | S100A13 | 1,00E+00 | -0,37 |
| STXBP1 | 1,08E+02 | 0,34 |  | TMSB10 | 8,00E-03 | -0,37 |
| ATF4 | 6,00E+08 | 0,34 |  | SLC38A2 | 1,00E+00 | -0,37 |
| SFPQ | 5,39E+08 | 0,34 |  | SLC6A14 | 2,09E-01 | -0,36 |
| TRAF4 | 4,18E+09 | 0,34 |  | PERP | 1,00E+00 | -0,36 |
| EIF3I | 9,80E+06 | 0,34 |  | MT-ND5 | 1,00E+00 | -0,36 |
| DNPH1 | 6,39E+04 | 0,34 |  | GUCA2A | 1,00E+00 | -0,36 |
| SLC25A5 | 3,15E+08 | 0,33 |  | CCL20 | 1,00E+00 | -0,36 |
| P4HB | 1,56E-02 | 0,33 |  | FDPS | 1,00E+00 | -0,36 |
| RPL41 | 7,75E+05 | 0,33 |  | ID2 | 1,00E+00 | -0,36 |
| PSMB2 | 1,77E+05 | 0,33 |  | TMPRSS2 | 1,00E+00 | -0,35 |
| NENF | 3,23E+09 | 0,33 |  | PHLDA3 | 2,31E-01 | -0,35 |
| FOXA3 | 5,46E+05 | 0,33 |  | YPEL5 | 1,00E+00 | -0,35 |
| NBEAL1 | 2,55E+08 | 0,33 |  | ZFP36L1 | 1,00E+00 | -0,35 |
| PSMA7 | 1,54E+08 | 0,33 |  | MYL12A | 1,00E+00 | -0,35 |
| TMEM263 | 4,53E+06 | 0,33 |  | TCEB2 | 9,82E-02 | -0,35 |
| STRAP | 1,22E+08 | 0,33 |  | PPARD | 1,00E+00 | -0,35 |
| PRDX1 | 2,02E-03 | 0,33 |  | SDC4 | 1,00E+00 | -0,35 |
| RPS14 | 5,23E+03 | 0,32 |  | CAMK2N1 | 1,00E+00 | -0,34 |
| LSM5 | 8,40E-04 | 0,32 |  | PRDX5 | 1,00E+00 | -0,34 |
| SNRPE | 1,27E-04 | 0,32 |  | CTSA | 1,00E+00 | -0,34 |
| MANF | 3,15E-03 | 0,32 |  | YPEL3 | 1,00E+00 | -0,34 |
| C14orf166 | 1,85E+07 | 0,32 |  | CDH1 | 1,00E+00 | -0,34 |
| UBA2 | 1,01E+05 | 0,32 |  | PLAU | 1,00E+00 | -0,34 |
| MTDH | 5,03E+07 | 0,32 |  | CTD-3252C9.4 | 1,00E+00 | -0,34 |
| SLC12A2 | 7,79E+06 | 0,32 |  | SDC1 | 1,00E+00 | -0,34 |
| GNB2L1 | 1,02E+08 | 0,32 |  | SUN2 | 1,00E+00 | -0,34 |
| POLD2 | 1,08E+09 | 0,32 |  | MUC4 | 1,00E+00 | -0,34 |
| FOSL1 | 9,61E+08 | 0,32 |  | SLC44A4 | 1,00E+00 | -0,34 |
| METTL5 | 3,68E+03 | 0,32 |  | DUSP10 | 1,00E+00 | -0,34 |
| NUCB2 | 6,78E+03 | 0,32 |  | CAV2 | 1,00E+00 | -0,34 |
| EFNB2 | 3,99E+09 | 0,32 |  | KRT19 | 1,00E+00 | -0,34 |
| SUB1 | 2,26E-03 | 0,32 |  | PIM1 | 1,00E+00 | -0,33 |
| EIF5A | 9,23E+05 | 0,32 |  | C12orf75 | 1,00E+00 | -0,33 |
| RPL35A | 9,38E+04 | 0,32 |  | ADAM8 | 1,00E+00 | -0,33 |
| CNBP | 1,46E+07 | 0,32 |  | FUS | 1,00E+00 | -0,33 |
| ZFP36 | 4,41E-01 | 0,32 |  | MXD1 | 1,00E+00 | -0,33 |
| HMGB2 | 4,56E-04 | 0,31 |  | POLR2J3 | 1,00E+00 | -0,33 |
| RAB4A | 2,01E+06 | 0,31 |  | CAPN2 | 1,00E+00 | -0,33 |
| TPSG1 | 3,41E+06 | 0,31 |  | KLK8 | 1,39E-03 | -0,33 |
| RANBP1 | 2,18E+08 | 0,31 |  | ABCA7 | 1,00E+00 | -0,33 |
| ETHE1 | 1,08E-04 | 0,31 |  | DNAJC5 | 1,00E+00 | -0,33 |
| RBMX | 7,30E+04 | 0,31 |  | KCNN4 | 1,00E+00 | -0,33 |
| RPL36A | 1,83E+09 | 0,31 |  | ARRDC4 | 1,00E+00 | -0,33 |
| MYDGF | 5,31E-04 | 0,31 |  | BAIAP2L1 | 1,00E+00 | -0,33 |
| DSTN | 5,16E-04 | 0,31 |  | MACC1 | 1,00E+00 | -0,33 |
| LMAN1 | 8,04E+07 | 0,31 |  | IER5 | 1,00E+00 | -0,32 |
| SIVA1 | 4,85E-04 | 0,31 |  | CYSRT1 | 1,00E+00 | -0,32 |
| NDUFB8 | 9,92E+06 | 0,31 |  | ADAMTS9 | 1,00E+00 | -0,32 |
| FAM3D | 1,10E-03 | 0,31 |  | PRKCDBP | 1,00E+00 | -0,32 |
| FOXP4 | 1,14E+07 | 0,31 |  | ZMYND8 | 1,00E+00 | -0,32 |
| NDUFC1 | 8,26E+07 | 0,31 |  | CARHSP1 | 1,00E+00 | -0,32 |
| RPS16 | 7,44E+03 | 0,31 |  | HN1 | 1,00E+00 | -0,32 |
| LINC00668 | 2,52E+03 | 0,31 |  | IFRD1 | 1,00E+00 | -0,32 |
| RPL24 | 1,54E+06 | 0,30 |  | DSG2 | 1,00E+00 | -0,32 |
| H2AFV | 1,49E-02 | 0,30 |  | TAF7 | 1,00E+00 | -0,32 |
| C16orf54 | 1,32E-01 | 0,30 |  | AES | 1,00E+00 | -0,32 |
| HSP90B1 | 1,09E-04 | 0,30 |  | ECE1 | 1,00E+00 | -0,32 |
| EEF1A1 | 1,07E-04 | 0,30 |  | TMEM160 | 1,00E+00 | -0,32 |
| SPCS1 | 2,56E+08 | 0,30 |  | WSB1 | 1,00E+00 | -0,32 |
| SNRPC | 2,46E+07 | 0,30 |  | TMC5 | 1,00E+00 | -0,31 |
| APRT | 4,35E+08 | 0,30 |  | FUT3 | 1,00E+00 | -0,31 |
| PABPC1 | 1,95E+09 | 0,30 |  | HP1BP3 | 1,00E+00 | -0,31 |
| BANF1 | 4,38E+09 | 0,30 |  | SLC25A29 | 1,00E+00 | -0,31 |
| MORF4L2 | 3,04E-04 | 0,30 |  | CLTB | 1,00E+00 | -0,31 |
| EIF4H | 4,47E+08 | 0,30 |  | CXADR | 1,00E+00 | -0,31 |
| SLC25A33 | 9,52E+05 | 0,30 |  | UBE2H | 1,00E+00 | -0,31 |
| NDUFAB1 | 7,18E+09 | 0,30 |  | PLOD2 | 1,00E+00 | -0,31 |
| MAPK13 | 1,34E+06 | 0,30 |  | SPON2 | 1,00E+00 | -0,31 |
| DDX21 | 1,25E+09 | 0,30 |  | EFHD2 | 1,00E+00 | -0,31 |
| NDUFB2 | 1,03E+08 | 0,30 |  | HSBP1L1 | 1,00E+00 | -0,31 |
| RAP2B | 2,19E-03 | 0,29 |  | ERBB2IP | 1,00E+00 | -0,31 |
| HSD17B10 | 3,23E+02 | 0,29 |  | UNC93B1 | 1,00E+00 | -0,31 |
| UBE2S | 2,21E-03 | 0,29 |  | RNMT | 1,00E+00 | -0,31 |
| CLDN3 | 1,29E-01 | 0,29 |  | SOD2 | 1,00E+00 | -0,30 |
| SEPP1 | 3,29E-04 | 0,29 |  | MLPH | 1,00E+00 | -0,30 |
| SSU72 | 4,61E-04 | 0,29 |  | MLLT4 | 1,00E+00 | -0,30 |
| MRPL23 | 6,56E+08 | 0,29 |  | ABHD11 | 1,00E+00 | -0,30 |
| RND1 | 1,31E+07 | 0,29 |  | RRAS | 1,00E+00 | -0,30 |
| RPS15A | 1,99E+07 | 0,29 |  | TMC6 | 1,00E+00 | -0,30 |
| COMMD6 | 1,64E-03 | 0,29 |  | RAB4B | 1,00E+00 | -0,30 |
| EEF2 | 7,84E-03 | 0,29 |  | GNAI2 | 1,00E+00 | -0,30 |
| KIAA0040 | 1,03E+09 | 0,29 |  | ANGPTL4 | 1,00E+00 | -0,30 |
| FAM136A | 1,58E+07 | 0,29 |  | CLDN23 | 1,00E+00 | -0,30 |
| TTC39A | 3,21E+07 | 0,29 |  | TMEM165 | 1,00E+00 | -0,30 |
| ENSA | 1,81E-04 | 0,29 |  | MAP1LC3B | 1,00E+00 | -0,30 |
| PTGES3 | 9,56E-04 | 0,29 |  | SLC17A9 | 1,00E+00 | -0,30 |
| MLEC | 1,22E+09 | 0,29 |  | LMTK3 | 1,00E+00 | -0,30 |
| CDX1 | 1,54E+05 | 0,29 |  | EHBP1L1 | 1,00E+00 | -0,30 |
| ECHS1 | 2,32E+07 | 0,29 |  | SCAND1 | 1,00E+00 | -0,30 |
| ATP5C1 | 7,52E+09 | 0,29 |  | RHOB | 1,00E+00 | -0,30 |
| MESDC1 | 2,18E+06 | 0,28 |  | LITAF | 1,00E+00 | -0,30 |
| CHPT1 | 3,09E+07 | 0,28 |  | PMEPA1 | 1,00E+00 | -0,30 |
| UBALD1 | 5,20E+05 | 0,28 |  | CDKN1C | 1,00E+00 | -0,30 |
| PLP2 | 4,30E-03 | 0,28 |  | TSC22D3 | 1,00E+00 | -0,30 |
| LGALS4 | 5,78E+07 | 0,28 |  | AMIGO2 | 1,00E+00 | -0,30 |
| ARL6IP4 | 3,23E+08 | 0,28 |  | LDHA | 1,00E+00 | -0,29 |
| CKAP4 | 5,00E+09 | 0,28 |  | AVPI1 | 1,00E+00 | -0,29 |
| RPL37 | 3,15E+06 | 0,28 |  | PLK3 | 1,00E+00 | -0,29 |
| NHP2 | 1,11E+06 | 0,28 |  | ST14 | 1,00E+00 | -0,29 |
| RPL10 | 3,12E+07 | 0,28 |  | ARL6IP1 | 1,00E+00 | -0,29 |
| RPL29 | 2,47E+06 | 0,28 |  | UBB | 1,00E+00 | -0,29 |
| COX7A2L | 3,71E-04 | 0,28 |  | NR1D1 | 1,00E+00 | -0,29 |
| GHITM | 2,11E+08 | 0,28 |  | CDA | 1,00E+00 | -0,29 |
| LSM4 | 4,55E+07 | 0,28 |  | IRF7 | 1,00E+00 | -0,29 |
| GGCT | 5,20E+07 | 0,28 |  | MAFB | 1,00E+00 | -0,29 |
| ATP5J | 1,49E-02 | 0,28 |  | SMIM5 | 1,00E+00 | -0,29 |
| PYURF | 3,04E+08 | 0,28 |  | ITGA3 | 1,00E+00 | -0,29 |
| ARMC10 | 2,77E+09 | 0,28 |  | RHOD | 1,00E+00 | -0,29 |
| KCTD12 | 1,05E+07 | 0,28 |  | VAMP8 | 1,00E+00 | -0,29 |
| DNAJC19 | 9,31E+05 | 0,28 |  | GCNT3 | 1,00E+00 | -0,28 |
| COPS6 | 1,85E+08 | 0,28 |  | DUOX2 | 1,00E+00 | -0,28 |
| GGH | 4,07E+05 | 0,28 |  | MFI2 | 1,00E+00 | -0,28 |
| RPS26 | 4,06E-04 | 0,27 |  | AHNAK2 | 1,00E+00 | -0,28 |
| EIF3F | 9,35E+09 | 0,27 |  | PELI1 | 1,00E+00 | -0,28 |
| COX8A | 7,50E-04 | 0,27 |  | FAM129B | 1,00E+00 | -0,28 |
| ARF6 | 2,10E-03 | 0,27 |  | RNF103 | 1,00E+00 | -0,28 |
| SMCO4 | 7,23E+07 | 0,27 |  | DSP | 1,00E+00 | -0,28 |
| GUSB | 2,09E+08 | 0,27 |  | TNFAIP3 | 1,00E+00 | -0,28 |
| MRPL11 | 2,57E+03 | 0,27 |  | HOOK3 | 1,00E+00 | -0,28 |
| SMIM19 | 1,04E+06 | 0,27 |  | RSRC2 | 1,00E+00 | -0,28 |
| ERI3 | 6,18E+04 | 0,27 |  | WFDC3 | 1,00E+00 | -0,28 |
| ACP1 | 1,37E+09 | 0,27 |  | DOCK6 | 1,00E+00 | -0,28 |
| CUTA | 2,04E-04 | 0,27 |  | HK2 | 1,00E+00 | -0,28 |
| MRPL37 | 1,06E+08 | 0,27 |  | NOSIP | 1,00E+00 | -0,28 |
| PPP1R14B | 4,54E+09 | 0,27 |  | CTSS | 1,00E+00 | -0,27 |
| ST6GALNAC4 | 1,16E+06 | 0,27 |  | EGLN3 | 1,00E+00 | -0,27 |
| MATN2 | 2,48E+02 | 0,27 |  | CTNNA1 | 1,00E+00 | -0,27 |
| PCBD1 | 2,46E+08 | 0,27 |  | CLOCK | 1,00E+00 | -0,27 |
| PCBP2 | 6,77E-02 | 0,27 |  | TMEM120B | 1,00E+00 | -0,27 |
| FAM195A | 1,30E+08 | 0,27 |  | IFI27 | 1,00E+00 | -0,27 |
| TOMM7 | 1,95E-02 | 0,27 |  | N4BP2L2 | 1,00E+00 | -0,27 |
| CYC1 | 6,43E+06 | 0,27 |  | PKN2 | 1,00E+00 | -0,27 |
| KRT18 | 3,07E-03 | 0,27 |  | SEPW1 | 1,00E+00 | -0,27 |
| SLC44A3 | 5,87E+06 | 0,27 |  | GBP2 | 1,00E+00 | -0,27 |
| EIF3L | 3,88E+07 | 0,27 |  | SRD5A3 | 1,00E+00 | -0,27 |
| CD74 | 4,96E+09 | 0,27 |  | TMEM63A | 1,00E+00 | -0,27 |
| SF3B5 | 7,38E+09 | 0,27 |  | PKP3 | 1,00E+00 | -0,27 |
| TMA7 | 2,21E-04 | 0,27 |  | RDH10 | 1,00E+00 | -0,27 |
| MARCKSL1 | 6,96E-02 | 0,27 |  | CD44 | 1,00E+00 | -0,27 |
| CCL15 | 9,63E+05 | 0,27 |  | HIST1H2BD | 1,00E+00 | -0,27 |
| PHB2 | 2,35E+08 | 0,27 |  | RSRP1 | 1,00E+00 | -0,27 |
| SRSF3 | 2,67E-03 | 0,27 |  | PPP1R16A | 1,00E+00 | -0,26 |
| RPA3 | 3,74E-03 | 0,26 |  | PIEZO1 | 1,00E+00 | -0,26 |
| BCL7B | 5,43E+08 | 0,26 |  | ARRDC2 | 1,00E+00 | -0,26 |
| TMEM14B | 6,76E+09 | 0,26 |  | CXCL6 | 1,00E+00 | -0,26 |
| ATPIF1 | 4,15E+07 | 0,26 |  | IL23A | 1,00E+00 | -0,26 |
| C2 | 6,43E+04 | 0,26 |  | DTX2 | 1,00E+00 | -0,26 |
| ATP5A1 | 1,00E-04 | 0,26 |  | MMP14 | 1,00E+00 | -0,26 |
| NQO1 | 3,63E-04 | 0,26 |  | ALS2CL | 1,00E+00 | -0,26 |
| ALDH1A1 | 1,01E+06 | 0,26 |  | SPATS2L | 1,00E+00 | -0,26 |
| TCEA1 | 1,66E+08 | 0,26 |  | EGR1 | 1,00E+00 | -0,26 |
| EBPL | 2,11E+03 | 0,26 |  | HMGA1 | 1,00E+00 | -0,26 |
| BIRC5 | 1,38E-03 | 0,26 |  | TTF1 | 1,00E+00 | -0,26 |
| MANBAL | 1,02E+08 | 0,26 |  | XIST | 1,00E+00 | -0,26 |
| RPL37A | 1,05E+07 | 0,26 |  | MST1R | 1,00E+00 | -0,26 |
| JAGN1 | 1,11E+07 | 0,26 |  | DST | 1,00E+00 | -0,26 |
| ANKH | 5,91E+04 | 0,26 |  | OSR2 | 1,00E+00 | -0,26 |
| EMD | 2,86E+07 | 0,26 |  | PLS1 | 1,00E+00 | -0,26 |
| LAGE3 | 2,61E+09 | 0,26 |  | PTBP3 | 1,00E+00 | -0,26 |
| COX5A | 3,89E-03 | 0,26 |  | C10orf10 | 1,00E+00 | -0,26 |
| RPL39 | 4,89E+07 | 0,26 |  | CFLAR | 1,00E+00 | -0,26 |
| NUCKS1 | 7,68E-04 | 0,26 |  | CTSZ | 1,00E+00 | -0,25 |
| IFT20 | 8,71E+06 | 0,26 |  | HIP1R | 1,00E+00 | -0,25 |
| MGST1 | 8,52E+09 | 0,26 |  | MXRA5 | 1,00E+00 | -0,25 |
| SMDT1 | 2,26E+08 | 0,26 |  | ABTB1 | 1,00E+00 | -0,25 |
| PDXDC1 | 3,91E+06 | 0,26 |  | PRPF4B | 1,00E+00 | -0,25 |
| C17orf89 | 1,74E+09 | 0,26 |  | AMN | 1,00E+00 | -0,25 |
| PLPP5 | 1,33E+07 | 0,26 |  | ARHGAP27 | 1,00E+00 | -0,25 |
| PFN1 | 8,07E-03 | 0,25 |  | USP53 | 1,00E+00 | -0,25 |
| DAP | 5,53E-04 | 0,25 |  | MYL9 | 1,00E+00 | -0,25 |
| TMEM14A | 3,72E+09 | 0,25 |  | VPS4B | 1,00E+00 | -0,25 |
| FHL2 | 1,81E-02 | 0,25 |  | DYNC1I2 | 1,00E+00 | -0,25 |
| KLK1 | 3,13E+09 | 0,25 |  | PLCXD1 | 1,00E+00 | -0,25 |
| RPL23 | 2,09E-03 | 0,25 |  | AIM1L | 1,00E+00 | -0,25 |
| HNRNPA0 | 1,23E-04 | 0,25 |  | RNF223 | 1,00E+00 | -0,25 |
| TM9SF3 | 4,37E-03 | 0,25 |  | HERPUD1 | 1,00E+00 | -0,25 |
| LINC01207 | 1,45E-04 | 0,25 |  | KMT2E | 1,00E+00 | -0,25 |
| UBE2L6 | 8,64E+03 | 0,25 |  | PLA2G16 | 1,00E+00 | -0,25 |
| NDUFAF4 | 1,91E+04 | 0,25 |  |  |  |  |
| MMAB | 6,85E+07 | 0,25 |  |  |  |  |
| ZNF706 | 1,93E-01 | 0,25 |  |  |  |  |
| AKR7A2 | 2,70E+07 | 0,25 |  |  |  |  |
| SPDEF | 8,68E+08 | 0,25 |  |  |  |  |
| MRPS15 | 1,02E+06 | 0,25 |  |  |  |  |

**Table S3 – Differential gene expression analysis of FAP-positive and FAP-negative tumor cells.** Single cell RNA expression data from colon tumors (cohorts GSE144735 and GSE132465) were used for differential gene expression analysis between FAP–expressing and non-expressing tumor cells defined by non-zero or zero-value FAP counts respectively, using the FindMarkers function with default parameters (wilcox test, logfc.threshold = 0.25).
